# Risk of Intestinal Parasitic Infections in People with Different Exposures to Wastewater and Fecal Sludge in Kampala, Uganda: A Cross-Sectional Study

**DOI:** 10.1371/journal.pntd.0004469

**Published:** 2016-03-03

**Authors:** Samuel Fuhrimann, Mirko S. Winkler, Narcis B. Kabatereine, Edridah M. Tukahebwa, Abdulla A. Halage, Elizeus Rutebemberwa, Kate Medlicott, Christian Schindler, Jürg Utzinger, Guéladio Cissé

**Affiliations:** 1 Department of Epidemiology and Public Health, Swiss Tropical and Public Health Institute, Basel, Switzerland; 2 University of Basel, Basel, Switzerland; 3 Schistosomiasis Control Initiative, Imperial College London, London, United Kingdom; 4 Vector Control Division, Ministry of Health, Kampala, Uganda; 5 School of Public Health, Makerere University, Kampala, Uganda; 6 Department of Public Health and Environment, World Health Organization, Geneva, Switzerland; George Washington University, UNITED STATES

## Abstract

**Background:**

There are health risks associated with wastewater and fecal sludge management and use, but little is known about the magnitude, particularly in rapidly growing urban settings of low- and middle-income countries. We assessed the point-prevalence and risk factors of intestinal parasite infections in people with different exposures to wastewater and fecal sludge in Kampala, Uganda.

**Methodology:**

A cross-sectional survey was carried out in September and October 2013, enrolling 915 adults from five distinct population groups: workers maintaining wastewater facilities; workers managing fecal sludge; urban farmers; slum dwellers at risk of flooding; and slum dwellers without risk of flooding. Stool samples were subjected to the Kato-Katz method and a formalin-ether concentration technique for the diagnosis of helminth and intestinal protozoa infections. A questionnaire was administered to determine self-reported signs and symptoms, and risk factors for intestinal parasite infections. Univariate and multivariate analyses, adjusted for sex, age, education, socioeconomic status, water, sanitation, and hygiene behaviors, were conducted to estimate the risk of infection with intestinal parasites and self-reported health outcomes, stratified by population group.

**Principal Findings:**

The highest point-prevalence of intestinal parasite infections was found in urban farmers (75.9%), whereas lowest point-prevalence was found in workers managing fecal sludge (35.8%). Hookworm was the predominant helminth species (27.8%). In urban farmers, the prevalence of *Trichuris trichiura*, *Schistosoma mansoni*, *Ascaris lumbricoides*, and *Entamoeba histolytica*/*E*. *dispar* was 15% and above. For all investigated parasites, we found significantly higher odds of infection among urban farmers compared to the other groups (adjusted odds ratios ranging between 1.6 and 12.9). In general, female participants had significantly lower odds of infection with soil-transmitted helminths and *S*. *mansoni* compared to males. Higher educational attainment was negatively associated with the risk of intestinal protozoa infections, while socioeconomic status did not emerge as a significant risk factor for any tested health outcome.

**Conclusions/Significance:**

Urban farmers are particularly vulnerable to infections with soil-transmitted helminths, *S*. *mansoni*, and intestinal protozoa. Hence, our findings call for public health protection measures for urban farmers and marginalized communities, going hand-in-hand with integrated sanitation safety planning at city level.

## Author Summary

Urban wastewater and fecal sludge use is of growing importance all over the world. However, unsafe management and inappropriate use might exacerbate the transmission of infectious diseases, including those caused by intestinal protozoa (e.g., amebiasis and giardiasis) and parasitic worms (e.g., soil-transmitted helminthiasis and schistosomiasis). People living and working in densely populated and rapidly transforming cities in Africa and Asia are especially vulnerable. We conducted a cross-sectional survey and assessed people’s risk of intestinal parasitic infections due to exposure to wastewater and fecal sludge management and use in Kampala, the capital of Uganda. We collected data on the prevalence, intensity, and risk factors of infections with parasitic worms and intestinal protozoa among slum dwellers, urban farmers, and workers maintaining the sanitation system. We found high infection prevalence of *Schistosoma mansoni* and soil-transmitted helminths in urban farmers and slum dwellers after adjusting for age, sex, and educational attainment. Our data suggest that urban farmers are especially vulnerable to infections with intestinal parasites, which may play an important role in the transmission through contamination of their living and working environments. In view of our results, the control of schistosomiasis and soil-transmitted helminthiasis should be accelerated in Kampala.

## Introduction

Africa and Asia are urbanizing faster than any other region of the world and an increase of 16% of the urban population is predicted for 2050 [1]. With such a demographic expansion, safe wastewater and fecal sludge management and use strategies are of pivotal importance for a healthy life in urban settings [2,3]. In the surroundings of densely populated urban centers of low- and middle-income countries (LMIC), inappropriate wastewater management is common [4,5]. Sanitation infrastructures often struggle to keep abreast of rapid population growth and increasing discharge of wastewater flows, including industrial effluents [6]. Consequently, people living and working in close proximity to wastewater management chains in urban settings of LMIC are frequently exposed to a broad range of pathogenic organisms and toxic chemicals [7,8]. Water-borne, water-related, water-washed, and water-based diseases (e.g., intestinal parasitic infections, diarrheal diseases, skin, and eye infections) are associated with a lack of safe sanitation practices [9–11]. Moreover, occupational exposure to wastewater and fecal sludge was reported to be associated to intestinal parasite infections [12]. For example, an association between infection with hookworm and *Schistosoma mansoni* was found with specific farming activities in a medium-sized town in Côte d’Ivoire [13]. Additionally, increased risk of intestinal nematode infection and hookworm infection, in particular, could be shown among farmers using wastewater in Pakistan and Vietnam [14,15].

For prevention and control of these infectious diseases, the provision of basic sanitation infrastructure, coupled with education and promotion in hygiene practices, and targeted drug administration proofed effective [9,16,17]. To design interventions, one needs to understand disease transmission in the public domain (under control of a household) and domestic domain (such as public places of work and recreational sites) [18]. Indeed, measures to prevent and control infections that give rise to diarrheal diseases need to be tailored to specific urban risks factors and exposure groups [19,20].

In Kampala, the capital of Uganda, more than 90% of the 1.8 million inhabitants rely on onsite sanitation facilities, such as pit latrines and septic tanks. A small portion of wastewater is conveyed to treatment plants, while most of the generated wastewater and fecal sludge is discharged, without treatment, in open storm water channels [21,22]. Along the channel from the wastewater treatment plant to the Lake Victoria, there are three major categories of workers exposed to wastewater along this system: (i) those maintaining the sanitation systems; (ii) those at the wastewater treatment plants; and (iii) farmers using the wastewater downstream in the Nakivubo wetland. Furthermore, flooding events are spreading the wastewater flows in the poor low-laying settlements, putting the concerned communities under risk of contaminations [23]. In these marginalized settlements, seasonal flooding events might exacerbate the unfavorable conditions of existing sanitation systems and water-related health risks [24,25]. Hence, direct contact to wastewater and contamination of food crops grown in the wetlands, fish, and drinking water in Lake Victoria are putting thousands of slum dwellers, urban farmers, and workers maintaining the system at risk of ill-health [26].

Infections with soil-transmitted helminths and *Schistosoma* spp. are of particular concern, as Kampala is endemic for both soil-transmitted helminthiasis and schistosomiasis [27,28]. Recent studies from rural and peri-urban areas around Kampala revealed *S*. *mansoni* and hookworm prevalence of 89% and 43%, respectively [29]. The prevalence of two of the most important intestinal protozoa *Giardia intestinalis* and *Entamoeba histolytica*/*E*. *dispar* was 12% and 10%, respectively [30]. At the onset of our study, the city authorities did not consider intestinal parasitic infections as an issue in these urban areas. However, there is a paucity of recent epidemiologic data [31,32]. Hence, there is a need for epidemiologic studies conducted in these heterogeneous urban communities to develop an evidence-base of risk factors related to wastewater use in different population groups in order to guide preventive measures [20,33]. Population groups to be targeted include marginalized slum dwellers, urban farmers, and workers managing the sanitation system [19].

We report findings from a cross-sectional parasitologic survey in selected population groups (farmers, workers, and local communities) exposed to wastewater and fecal sludge management and use activities in the Nakivubo area in Kampala. We aimed to determine the prevalence rates of intestinal parasitic infections and to assess the associations of disease risks with socioeconomic, environmental, and lifestyle factors in the different population groups. Our investigation is part of a larger study, comprising of an environmental assessment, a quantitative microbial risk assessment to determine the health risks related to microbial contamination, and the development and validation of a sanitation safety planning manual [20,21,26].

## Methods

### Ethics Statement

This manuscript has been developed according to the consolidated standards of reporting trials (CONSORT). The study protocol was approved by the institutional research commission of the Swiss Tropical and Public Health Institute (Swiss TPH; Basel, Switzerland; reference no. FK 106) and the Uganda National Council for Science and Technology (UNCST; Kampala, Uganda; reference no. HS 1487). Ethical approval was obtained from the ethics committee of the cantons of Basel-Stadt and Basel-Landschaft (EKBB; reference no. 137/13) and the Higher Degrees Research and Ethics Committee of Makerere University School of Public Health (Kampala, Uganda; reference no. IRBOOO11353). This study is registered with the clinical trial registry ISRCTN (identifier: ISRCTN13601686).

The following enrolment procedures were approved by the ethical committees: all participants were informed about the purpose and procedures of the study and they were invited to sign a written informed consent. In case of illiteracy, thumb-print and signature of a witness was requested. Those with informed consent were assigned a unique identifier. Results were communicated to participants and those found infected with any kind of helminth were treated according to national guidelines (e.g., a single 400 mg oral dose of albendazole against soil-transmitted helminth infection and a single 40 mg/kg oral dose of praziquantel against schistosomiasis).

### Study Design and Participants

**Fig 1:**
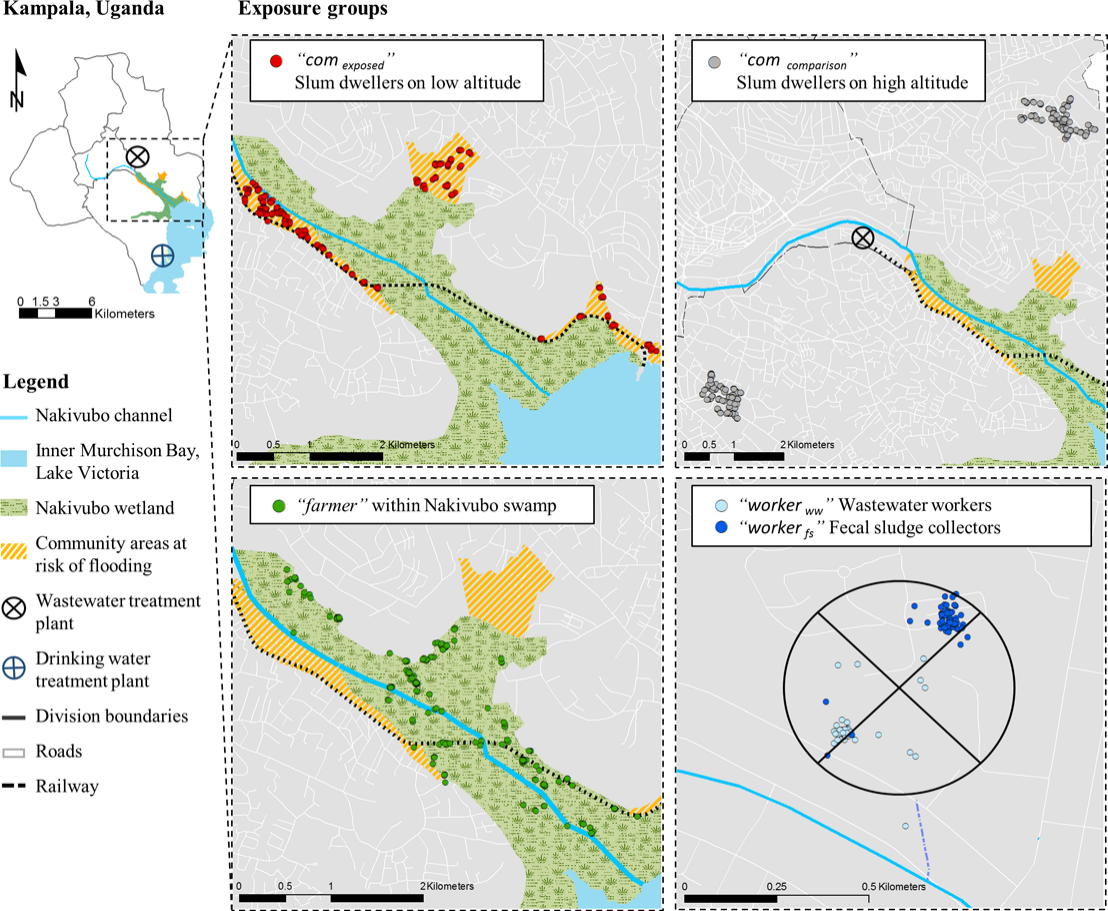
Map of Kampala showing the study area in the Nakivubo area. The exact geographic locations of all participants in the five exposure groups are indicated as follow: (i) red dots: “*com*_*exposed*_”, slum dwellers at risk of flooding along the Nakivubo wetland between altitudes 1,140 m and 1,160 m above mean sea level (AMSL); (ii) grey dots: “*com*_*comparison*_”, slum dwellers without risk of flooding at least 2 km away from the Nakivubo wetland and located between 1,160 and 1,201 m AMSL; (iii) green dots: “*farmer*”, urban farmers using wastewater within the Nakivubo wetland; (vi) dark blue dots: “*worker*_*ww*_”, workers maintaining drainage channels and operating the Bugolobi Sewage Treatment Works; and (v) light blue: “*worker*_*fs*_”, workers collecting fecal sludge at household level by means of vacuum trucks.

We conducted a cross-sectional survey in September and October 2013 in Kampala. The study was undertaken in the Nakivubo area (Nakawa and Makindye divisions), which receives most of Kampala’s wastewater. The area is located at an altitude of 1,140 m above the mean sea level at latitude 0° 18’ 48.1” N and longitude 32° 36’ 43.86” E. Domestic and industrial wastewater is derived from the central division, while the treated effluent of the Bugolobi Sewage Treatment Works (BSTW) is collected in the Nakivubo channel, a 12.3 km long open storm water channel. This channel enters into the Nakivubo wetland (5.3 km^2^), where approximately 600 farmers pursue urban farming for their livelihood. Informal slum communities are at highest risk of flooding events, as they live along both sides of the wetland (approximate population size is 12,000 people). The water from the Nakivubo wetland is ultimately discharged into the Inner Murchison Bay at the shores of Lake Victoria, some 4 km ahead of where the water is pumped and treated to supply Kampala city with drinking water (Fig 1). The study area has been described in detail elsewhere, including a short video that provides additional contextual features [21].

### Exposure Groups

We focused on adults (aged ≥18 years) living and working in the Nakivubo area. According to the level of exposure to wastewater, the study participants were stratified into five groups, as follows:

1. “*com*_*exposed*_”, slum dwellers at risk of flooding living along the Nakivubo wetland at altitudes ranging between 1,140 m and 1,160 m above mean sea level (AMSL). The communities are characterized by poor housing and unimproved sanitation and unsafe water supply;
2. “*com*_*comparison*_”, slum dwellers living in similar communities as *com*_*exposed*_ without risk of flooding (comparison group) living at least 2 km away from the Nakivubo wetland at altitudes between 1,160 and 1,201 m AMSL;
3. “*farmer*”, urban farmers using informally and indirectly wastewater to grow sugar cane, yams, and maize within the Nakivubo wetland and living in the same communities as *com*_*exposed*_;
4. “*worker*_*ww*_”, workers employed by NWSC who maintain the drainage channels and operate the BSTW; and
5. “*worker*_*fs*_”, workers organised under the pit emptier association managing fecal sludge (e.g., collection at households by means of vacuum trucks).

### Sample Size and Inclusion Criteria

Our intended sample size was 1,000 participants (“*com*_*comparison*_” = 350, “*com*_*exposed*_” = 250, “*farmer*” = 275, “*worker*_*ww*_” = 50, and “*worker*_*fs*_” = 100). We aimed at a power of 95%, to ensure that a reduction in effective exposure variance by 35% following confounder adjustment would still leave 80% power. Our assumptions were that the prevalence rate of intestinal parasitic infections is at least 20% in “*com*_*comparison*_” and the difference in odds ratio (OR) to “*farmer*”, “*worker*_*ww*_”, and “*worker*_*fs*_” is at least 2.5. We also assumed that the final sample size would be reduced by 15% due to non-response and missing data.

The following inclusion and exclusion criteria were applied. “*com*_*comparison*_” and “*com*_*exposed*_” were selected proportionally to the projected number of individuals living in each village in 2013 [34]. Briefly, we applied a grid of 25 x 25 m over each village and randomly selected coordinates. At each cross point of the grid, we selected the closest four households. We used a Kish Grid to choose the participant at the unit of the household [35]. To select “*farmer*”, we mapped the on-going farming activities and estimated the number of farmers with the help of farmer chair persons. Our research team enrolled all farmers they encountered while visiting the farms between 7 a.m. and 6 p.m. over a 10-day period. To select “*worker*_*ww*_” and “*worker*_*fs*_”, we mobilized and informed the workers via the chair persons. All workers who showed up at their specific work sites over a period of 2 weeks were registered and invited to participate.

### Procedures

We used a questionnaire to determine exposure pathways to wastewater and fecal sludge, potential confounding factors (e.g., demographic and socioeconomic), risk variables (e.g., water, sanitation, hygiene, and occupation) and self-reported health outcomes. Study participants were asked about signs and symptoms experienced over the past 2 weeks before the interview took place, using a pre-tested questionnaire [36]. Diarrheal episodes were defined according to WHO as ‘the passage of three or more loose or liquid stools per day and assessed if the participant experienced an episode within the past 1, 7, or 14 days [37]. The questionnaire was developed in English, translated into the local language Luganda, and pre-tested with five farmers and five workers. Research assistants entered data directly into tablet computers (Samsung Galaxy note 10.1 N8010) via a data entry mask using Open Data Kit (http://opendatakit.org).

Participants were invited to provide a fresh morning stool that was subject to the Kato-Katz technique (duplicate thick smears, using standard 41.7 mg templates) [38] and a formalin-ether concentration technique (FECT) [39] for the diagnosis of helminths (*Ascaris lumbricoides*, hookworm, *Trichuris trichiura*, *S*. *mansoni*, and other helminths) and intestinal protozoa (*Blastocystis hominis*, *Chilomastix mesnili*, *Endolimax nana*, *Entamoeba coli*, *E*. *histolytica*/*E*. *dispar*, *Entamoeba hartmanni*, *G*. *intestinalis*, and *Iodamoeba bütschlii*).

### Statistical Analysis

Helminth- and intestinal protozoa-specific proportions between the five exposure groups were compared with Pearson’s χ^2^ test. Univariate logistic regression was applied to investigate the potential association between dependent (namely, infections with (i) any intestinal parasite, (ii) intestinal helminth, (iii) soil-transmitted helminth, (iv) intestinal protozoa, (v) *A*. *lumbricoides*, (vi) hookworm, (vii) *T*. *trichiura*, (viii) *S*. *mansoni*, (ix) 14-day diarrhoea, (x) skin problems, and (xi) eye problems) and 49 independent variables (e.g., sex and age). People’s socioeconomic status was determined using principal component analysis and participants were grouped into three categories, as indicated in Table 1 (most poor, poor, and less poor) [40]. Our multivariate core model included the categorical exposure variable, sex, age, level of education, and socioeconomic status [9,10]. We then added risk factors that had a p-value below 0.2 (using likelihood ratio test) in the univariate analyses.

**Table 1:** Demographic and socioeconomic characteristics of the participants enrolled in a cross-sectional survey conducted in late 2013 in Kampala, stratified by exposure group.

Demographic and socio-economic characteristics	*com *_comparison_^*^		*com* _exposed_^*^		farmer^*^		*worker *_*fs*_ ^*^		*worker* _ww_^*^	
	n = 331		n = 229		n = 245		n = 67		n = 43	
	n	%	n	%	n	%	n	%	n	%
**Sex**										
Female	233	70.4	171	74.7	110	44.9	0	0	4	9.3
Male	98	29.6	58	25.3	135	55.1	67	100	39	90.7
**Age range (years)**										
18–24	94	28.4	63	27.5	32	13.1	13	19.4	5	11.6
25–39	176	53.2	131	57.2	126	51.4	32	47.8	18	41.9
≥40	61	18.4	35	15.3	87	35.5	22	32.8	20	46.5
**Level of education**										
Never went to school	33	10.0	37	16.2	45	18.4	5	7.5	0	0
Primary school	103	31.1	102	44.5	144	58.8	15	22.4	4	9.3
'O' level	143	43.2	62	27.1	49	20.0	32	47.8	11	25.6
'A' level	29	8.8	21	9.2	4	1.6	11	16.4	7	16.3
Tertiary	20	6.0	7	3.1	2	0.8	4	6.0	13	30.2
University degree	3	0.9	0	0	1	0.4	0	0	8	18.6
**Socioeconomic status** (**principal component analysis (PCA))**^1^										
Most poor	99	29.9	87	38.0	112	45.7	0	0	1	2.3
Poor	127	38.4	73	31.9	88	35.9	17	25.4	6	14.0
Less poor	105	31.7	69	30.1	45	18.4	50	74.6	36	83.7
**Residential area (division)**										
Central	0	0	0	0	1	0.4	1	1.5	4	9.3
Nakawa	140	42.3	79	34.5	112	45.7	9	13.4	11	25.6
Kawempe	0	0	0	0	0	0	9	13.4	4	9.3
Rubaga	0	0	0	0	0	0	6	9.0	2	4.7
Makindye	191	57.7	150	65.5	132	53.9	16	23.9	13	30.2
of Kampala	0	0	0	0	0	0	26	38.8	9	20.9

^1^Principal component analysis (PCA) based on the possession of the following 11 items: radio, TV, mobile phone, fridge, computer, bicycle, motorbike, car, electricity, running water, and latrine. Categories of socioeconomic status were obtained by dividing the first principal component into tertiles.

^*^“com_*exposed*_*”*, slum dwellers at risk of flooding along the Nakivubo wetland; *“com*_*comparison*_*”*, slum dwellers without risk of flooding at least 2 km away from the Nakivubo wetland; *“farmer”*, urban farmers reusing wastewater within the Nakivubo wetland; *“worker*_*ww*_*”*, workers maintaining drainage channels and operate the Bugolobi Sewage Treatment Works; *“worker*_*fs*_*”*, workers managing fecal sludge (e.g., collection at households by means of vacuum trucks).

Odds ratios are reported to compare risks, while differences and associations are considered as statistically significant if p-values are below 0.05, and indicating a trend if p-values are between 0.05 and 0.1. Statistical analyses were done using STATA version 12.0 (Stata Corporation; College Station, United States of America). Maps, including geographic coordinates of the interviews, were established in ArcMap version 10 (Environmental System Research Institute; Redlands, United States of America). Kato-Katz thick smear and FECT readings were double-entered and validated.

## Results

### Participant Enrolment

**Fig 2:**
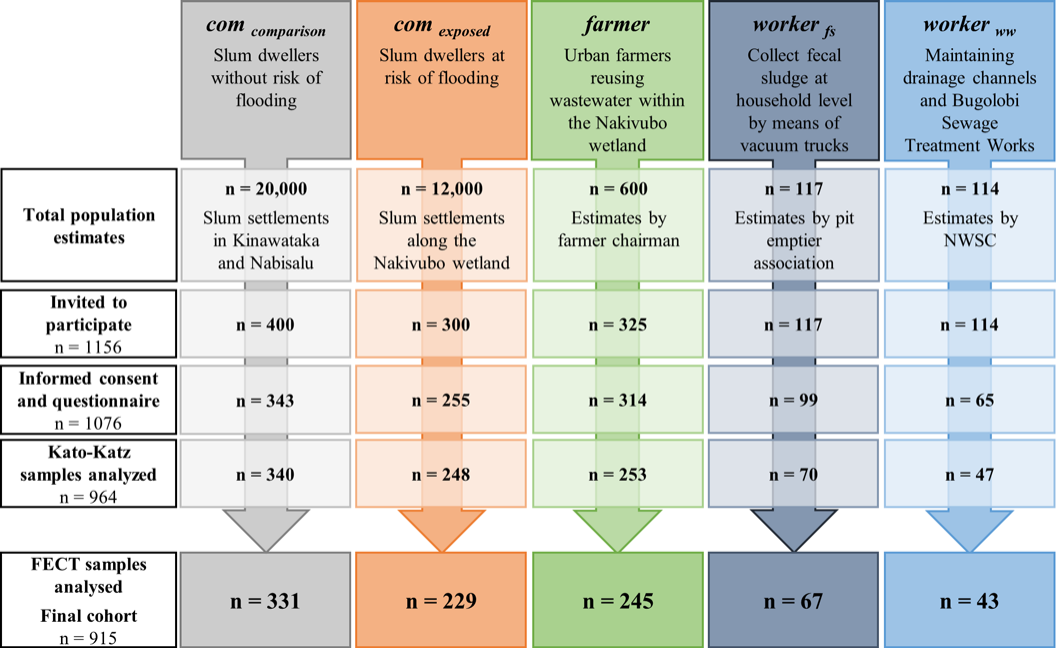
CONSORT flowchart showing study participation and compliance of the five specific exposure groups from October-November 2013. Flowchart shows the number of people who were invited, those who participated, and those with complete data records included in the final statistical analyses.

Among the 1,156 people invited, 1,076 fulfilled inclusion criteria, had written informed consent, and completed the questionnaire interview (Fig 2). Stool samples were provided by 964 individuals and subjected to Kato-Katz. Due to insufficient volumes of stool provided, only 915 of the samples were subjected to FECT, thus defining the final study cohort. As shown in Fig 2, the final cohort consisted of 229 “*com*_*exposed*_”, 331 “*com*_*comparison*_”, 245 “*farmer*”, 43, “*worker*_*ww*_”, and 67 “*worker*_*fs*_”.

### Study Population Characteristic

Table 1 shows the demographic (sex, age, educational attainment, religion, ethnicity, and division of living) and socioeconomic characteristics of the participants. Women accounted for 74%, 70%, 45% and 9% in “*com*_*exposed*_”, “*com*_*comparison*_”, “*farmer*”, and “*worker*_*ww*_”, respectively, whereas no woman was in the “*worker*_*fs*_” group. Socioeconomic status was highest in “*worker*_*fs*_” and “*worker*_*ww*_” with 83% and 74%, respectively, classified as less poor. The lowest socioeconomic status was observed in “*com*_*comparison*_”, “*com*_*exposed*_”, and “*farmer*” with 30%, 38%, and 45% classified as most poor, respectively.

Parameters for exposure to wastewater, access to drinking water, sanitation, and hygienic behaviors are summarized in S1A Table. Flooding events of the household occurred most often in households of “*farmer*”, and “*com*_*exposed*_” (64% and 47%, respectively). 65%, 49%, and 56% of the participants from “*com*_*exposed*_”, “*farmer*”, and “*com*_*comparison*_” had a toilet at home. Overall, 29% of all participants reported having taken a deworming drug within the past 6 months with the highest proportions reported by “*worker*_*fs*_” (61%).

S1B Table shows occupational conditions and risk factors for “*farmer*”, “*worker*_*ww*_”, and “*worker*_*fs*_”. While most of the workers are officially contracted (97%), the opposite is seen among “*farmer*”, as most lack an official employment status (95%). 81%, 63%, and 49% of “*worker*_*ww*_”, “*worker*_*fs*_”, and “*farmer*” wear boots, respectively. Only 4% of “*farmer*” use gloves, whilst over 80% of the workers use them.

### Prevalence and Intensity of Intestinal Parasitic Infections, Self-Reported Signs and Symptoms

**Fig 3:**
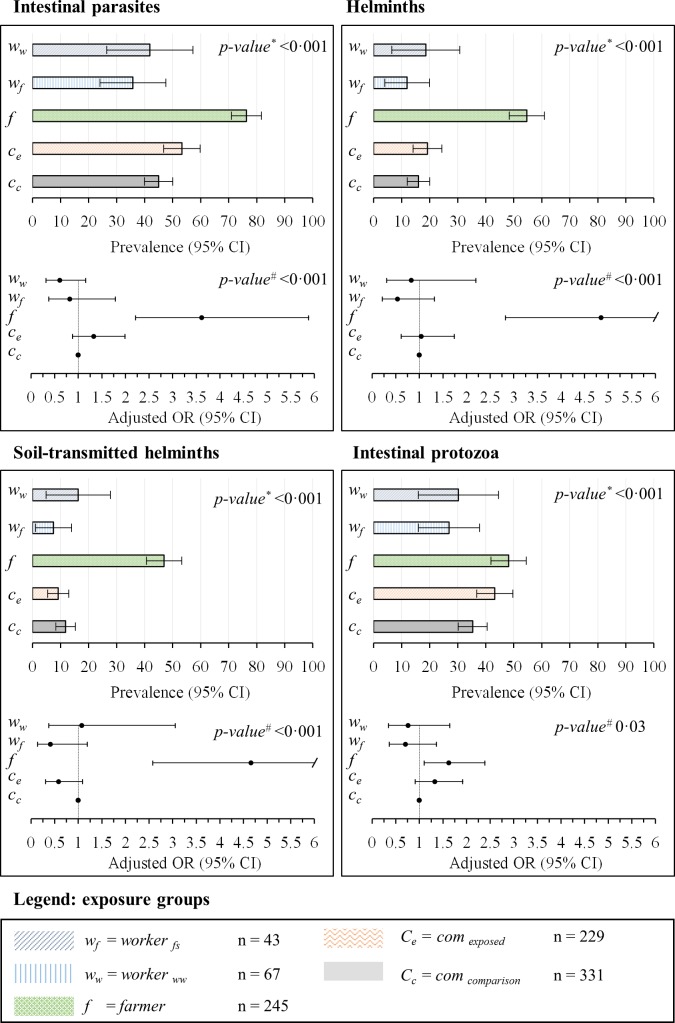
Prevalence rates and adjusted odds ratio (OR) and 95% confidence intervals (CIs). Values are indicated for “*com*_*exposed*_”, “*com*_*comparison*_”, “*farmer*”, “*worker*_*ww*_”, and “*worker*_*fs*_”for intestinal parasitic infections, intestinal helminth infections, soil-transmitted helminth infections, and intestinal protozoa infection. ^*^p-value based on χ^2^ test. ^#^p-value based on multivariate regression using likelihood ratio test.

**Fig 4:**
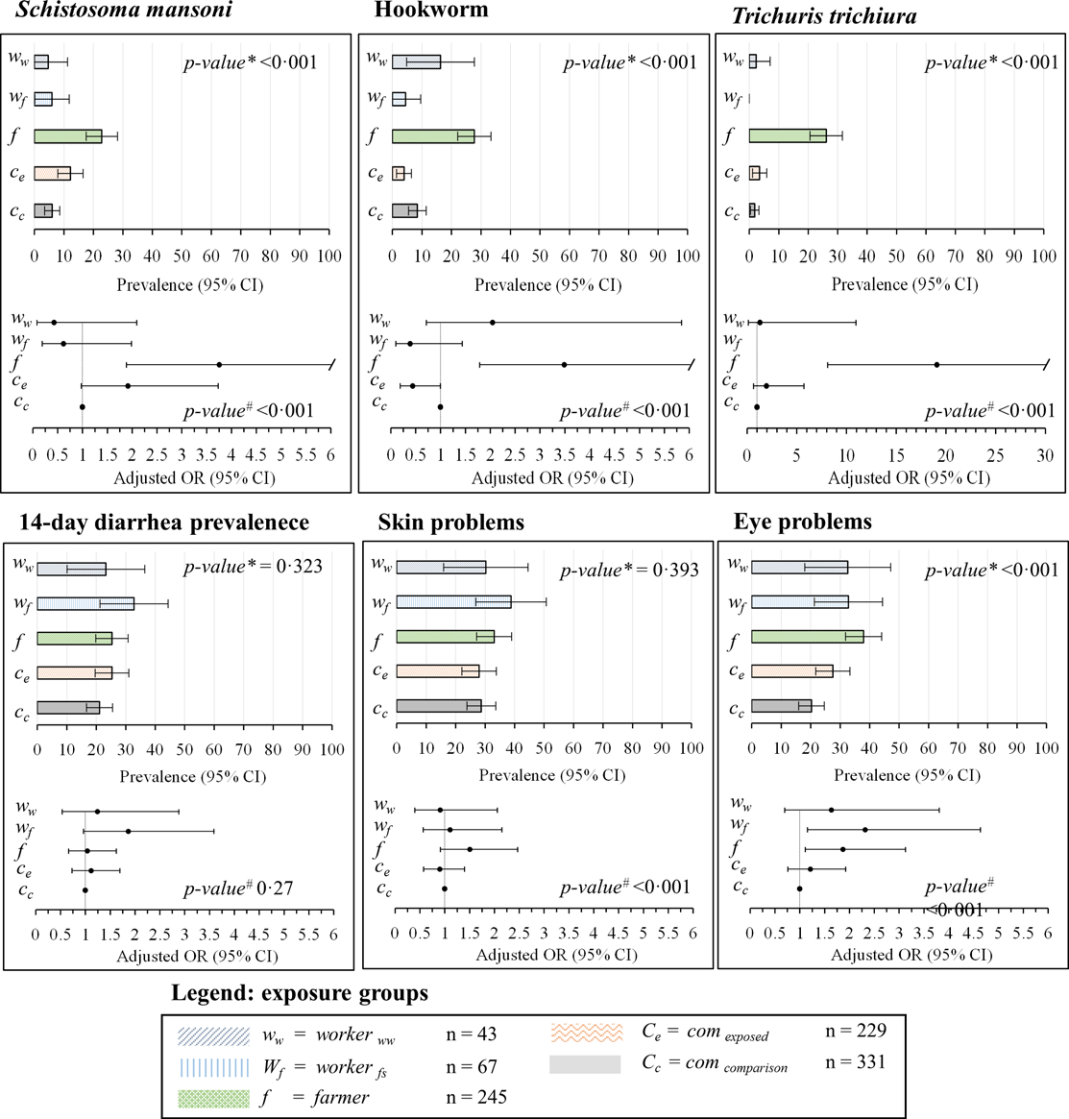
Prevalence rates and adjusted odds ratio (OR) and 95% confidence intervals (CIs). Values are indicated for “*com*_*exposed*_”, “*com*_*comparison*_”, “*farmer*”, “*worker*_*ww*_”, and “*worker*_*fs*_”for *Schistosoma mansoni*, hookworm, *Trichuris trichiura*, self-reported diarrhea, skin problems, and eye problems. ^*^p-value based on χ^2^ test. ^#^p-value based on multivariate regression using likelihood ratio test.

The prevalence and intensity of parasitic infections, stratified by exposure group, are summarized in Figs 3 and 4 and Table 2. The overall prevalence of infection with any intestinal parasite was (from highest to lowest) 76%, 53%, 44%, 42%, and 35% in “*farmer*”, “*com*_*exposed*_”, “*com*_*comparison*_”, “*worker*_*ww*_”, and “*worker*_*fs*_”, respectively. One quarter (25%) of all participants was found infected with at least two species of intestinal parasites. The highest prevalence of soil-transmitted helminth infection was found in “*farmer*” (hookworm, *T*. *trichiura*, and *A*. *lumbricoides* prevalence of 28%, 26%, and 18%, respectively). *S*. *mansoni* was detected in all exposure groups with prevalences of 5% and above; while the highest prevalence was found in “*farmer*” (23%). Nine participants were infected with *Hymenolepis nana* (six cases occurred in “*farmer*”), two with *Taenia* spp., and one with *Strongyloides* spp. Overall, 11 participants were found with heavy *S*. *mansoni* infection (≥400 eggs per gram of stool). Forty percent of all participants were infected with intestinal protozoa; the highest prevalence rates occurred in “*farmer*” and “*com*_*exposed*_” (48% and 43%, respectively). We found a prevalence of *E*. *histolytica/E*. *dispar* of 15%, 12%, and 7% in “*farmer*”, “*worker*_*ww*_”, and “*worker*_*fs*_”, respectively. Nine people had an infection with *G*. *intestinalis*, five of them in “*com*_*comparison*_”.

**Table 2:** Prevalence and intensity of parasitic infections of the participants enrolled in a cross-sectional survey conducted in late 2013 in Kampala, stratified by exposure groups.

Prevalence of infection^#^	*com *_comparison_^*^		*com* _exposed_^*^		farmer^*^		*worker *_*fs*_ ^*^		*worker* _ww_^*^		Difference (χ^2^)
	n = 331		n = 229		n = 245		n = 67		n = 43		
	n	%	n	%	n	%	n	%	n	%	p-value
**Intestinal parasite**	148	44.7	122	53.3	186	75.9	24	35.8	18	41.9	<0.001
**Helminth**	52	15.7	44	19.2	134	54.7	8	12.0	8	18.6	<0.001
**Soil-transmitted helminth**	39	11.8	21	9.2	115	46.9	5	7.5	7	16.3	<0.001
**Intestinal protozoa**	117	35.4	99	43.2	118	48.2	18	26.9	13	30.2	<0.001
**Hookworm**	28	8.5	9	3.9	68	27.8	3	4.5	7	16.3	<0.001
Light infection	28	8.5	8	3.5	64	26.1	2	3.0	7	16.3	
Moderate infection	0	0	1	0.4	4	1.6	1	1.5	0	0	<0.001
***Trichuris trichiura***	6	1.8	8	3.5	64	26.1	0	0	1	2.3	<0.001
Light infection	6	1.8	8	3.5	61	24.9	0	0	1	2.3	
Moderate infection	0	0	2	0.9	10	4.1	0	0	0	0	<0.001
***Ascaris lumbricoides***	0	0	7	3.1	45	18.4	0	0	1	2.3	<0.001
Light infection	0	0	5	2.2	35	14.3	0	0	1	2.3	
Moderate infection	0	0	2	0.9	10	4.1	0	0	0	0	<0.001
***Schistosoma mansoni***	20	6.0	28	12.2	56	22.9	4	6.0	2	4.7	<0.001
Light infection	11	3.3	21	9.2	37	15.1	2	3.0	1	2.3	
Moderate infection	7	2.1	3	1.3	15	6.1	1	1.5	1	2.3	
Heavy infection	2	0.6	4	1.8	4	1.6	1	1.5	0	0	<0.001
***Taenia* spp.**	2	0.6	0	0	0	0	0	0	0	0	0.472
***Hymenolepis nana***	2	0.6	0	0	6	2.5	1	1.5	0	0	0.067
***Strongyloides* spp.**	0	0	0	0	0	0	1	1.5	0	0	0.013
***Entamoeba histolytica/ E*. *dispar***	20	6.0	9	3.9	37	15.1	5	7.5	5	11.6	<0.001
***Entamoeba coli***	92	27.8	83	36.2	94	38.4	13	19.4	8	18.6	<0.001
***Giardia intestinalis***	5	1.5	1	0.4	2	0.8	1	1.5	0	0	0.677
***Balantidium coli***	1	0.3	0		1	0.4	0	0	0	0	0.869
***Chilomastix mesnili***	1	0.3	1	0.4	1	0.4	0	0	1	2.3	0.411
***Entamoeba hartmanni***	12	3.6	16	7.0	1	0.4	0	0	0	0	<0.001
***Iodamoeba bütschlii***	13	3.9	10	4.4	11	4.5	1	1.5	0	0	0.527

^*^“com_*exposed*_*”*, slum dwellers at risk of flooding along the Nakivubo wetland; *“com*_*comparison*_*”*, slum dwellers without risk of flooding at least 2 km away from the Nakivubo wetland; *“farmer”*, urban farmers reusing wastewater within the Nakivubo wetland; *“worker*_*ww*_*”*, workers maintaining drainage channels and operating the Bugolobi Sewage Treatment Works; *“worker*_*fs*_*”*, workers managing fecal sludge (e.g., collection at households by means of vacuum trucks).

^#^Prevalence is calculated out of the results of the examination of a single stool sample by means of duplicate Kato-Katz thick smears and the formalin-ether concentration method. Infection intensity is based on the examination of duplicate Kato-Katz thick smears.

Self-reported signs and symptoms are summarized in Fig 4 and Table 3. The prevalence of diarrhea (recall period: 2 weeks) was not significantly different between study groups; we found prevalence of 21% in “*com*_*comparison*_”, 25% in “*farmer*”, and 33% in “*worker*_*fs*_”. General skin problems were reported by between 28% (“*com*_*exposed*_” and “*com*_*comparison*_”) and 39% (*worker*_*fs*_). More specific skin irritation was reported by 19%, 16%, 13%, and 12% in “*worker*_*fs*_”, “*worker*_*ww*_”, “*com*_*exposed*_”, and “*farmer*”, whereas a considerable lower rate of 4% was found in “*com*_*comparison*_”. Eye problems were most frequently reported by “*farmer*” (37%), followed by “*worker*_*ww*_” and “*worker*_*fs*_” (33% each).

**Table 3:** Self-reported health outcomes experienced in the last 2 weeks before the interview among participants enrolled in a cross-sectional survey carried out in late 2013 in Kampala, stratified by exposure groups.

Self-reported health problems over the past 2 weeks	*com *_comparison_^*^		*com* _exposed_^*^		farmer^*^		*worker *_fs_^*^		*worker* _ww_^*^		Difference (χ^2^)
	n = 331		n = 229		n = 245		n = 67		n = 43		
	n	%	n	%	n	%	n	%	n	%	p-value
**Diarrhea**											
14-day prevalence	70	21.2	58	25.3	62	25.3	22	32.8	10	23.3	0.323
Blood in stool	8	2.4	5	2.2	8	3.3	3	4.5	1	2.3	0.837
Number of episodes											
1	50	15.1	49	21.4	40	16.3	10	14.9	4	9.3	
2	15	4.5	4	1.8	14	5.7	9	13.4	3	7.0	
3	2	0.6	4	1.8	5	2.0	2	3.0	0	0	
4	3	0.9	0	0	2	0.8	0	0	3	7.0	<0.001
**Eye issues**											
Eye problems	67	20.2	63	27.5	93	38.0	22	32.8	14	32.6	<0.001
Eye irritation	29	8.8	38	16.6	42	17.1	12	17.9	11	25.6	<0.001
Sensitivity to light	35	10.6	24	10.5	54	22.0	10	14.9	8	18.6	0.001
Other eye problems	7	2.1	6	2.6	13	5.3	6	9.0	0	0.0	0.016
**Skin issues**											
Skin problems	95	28.7	64	28.0	81	33.1	26	38.8	13	30.2	0.393
Skin irritation	14	4.2	29	12.7	29	11.8	13	19.4	7	16.3	<0.001
Itching	65	19.6	46	20.1	52	21.2	16	23.9	11	25.6	0.853
Sores on skin	0	0.0	2	0.9	14	5.7	4	6.0	3	7.0	0.020
Ulcer on skin	4	1.2	3	1.3	5	2.0	4	6.0	0	0.0	0.070
Other skin problems	9	2.7	4	1.8	8	3.3	1	1.5	0	0.0	0.621

*“*com*_*exposed*_”, slum dwellers at risk of flooding along the Nakivubo wetland; “*com*_*comparison*_”, slum dwellers without risk of flooding at least 2 km away from the Nakivubo wetland; “*farmer*”, urban farmers reusing wastewater within the Nakivubo wetland; “*worker*_*ww*_”, workers maintaining drainage channels and operating the Bugolobi Sewage Treatment Works; “*worker*_*fs*_”, workers managing fecal sludge (e.g., collection at households by means of vacuum trucks).

### Associations between Risk/Confounding Factors and Health Outcomes of Interest

Figs 3 and 4 provide an overview of adjusted associations of all measured helminth and intestinal protozoa infections, 14-day diarrhea prevalence, and skin and eye problems among different exposure groups, as revealed by the multivariate regression analyses. “*farmer*” had a higher odds of all measured helminth and intestinal protozoa infections, compared to the other groups (adjusted OR between 1.6 and 12.9). Workers (both “*worker*_*ww*_”, and “*worker*_*fs*_”) had lower adjusted odds compared to “*com*_*comparison*_” for intestinal parasite infections, except for hookworm, where “*worker*_*fs*_” had increased risk (OR 2.8, 95% CI 0.9 to 1.9). However, for 14-day diarrhea prevalence, skin and eye problems, workers had similar or higher risks. For soil-transmitted helminth “*com*_*exposed*_” showed lower infection risks compared to “*com*_*comparison*_” (OR 0.6, 95% CI 0.3 to 1.1), while the risk of intestinal protozoa infection was elevated (OR 1.3, 95% CI 0.9 to 1.9). Compared to “*com*_*comparison*_”, “*com*_*exposed*_” were at higher risk of *S*. *mansoni* infection (OR 1.9, 95% CI 1.0 to 3.7), but at lower risk of hookworm infection (OR 0.4, 95% CI 0.2 and 1.0). Moreover, 14-day prevalence rates of diarrhea and skin symptoms according to self-reports were higher among “*worker*_*fs*_” and “*farmer*” compared to the other groups.

Table 4 summarizes associations of “any parasitic infection” and S2A–S2I Table of all measured helminth and intestinal protozoa infections, 14-day diarrhea prevalence, skin, and eye problems with risk and confounding factors observed in univariate and multivariate regression analyses. Significantly increased risks were observed among male participants for total intestinal parasite, helminth, soil-transmitted helminth, and *S*. *mansoni* infections. Relying on pit latrines or having no toilet facility was associated with significantly increased risk of “any parasitic infection”, soil-transmitted helminth, and *T*. *trichiura* infections. Moreover, hand washing after defecation and work was negatively associated with *T*. *trichiura* infections (OR 0.4, 95% CI 0.2 to 0.8 and OR 0.6, 95% CI 0.3 to 1.1, respectively). Higher level of education was negatively associated with intestinal protozoa infections (OR 0.6, 95% CI 0.4 to 1.0). On the other hand, 14-day prevalence rates of diarrhea and skin symptoms according to self-reports were higher among participants with higher socioeconomic status.

**Table 4:** Results of univariate and the multivariate logistic regression analysis for parasitic infection in a cross-sectional survey done in late 2013 in Kampala^§^.

Intestinal parasitic infection			Univariate logistic regression*				Multivariate logistic regression**			
N = 915 / N(cases) = 530			OR	95% CI		p-value	aOR	95% CI		p-value
Exposure group***		*com *_*comparison*_	1.00			**<0.001**				**<0.001**
		*com* _*exposed*_	1.39	0.99	1.96	0.061	1.33	0.88	2.01	0.173
		*farmer*	3.88	2.69	5.62	<0.001	3.61	2.22	5.88	<0.001
		*worker *_*fs*_	0.68	0.39	1.16	0.163	0.61	0.32	1.16	0.131
		*worker* _*ww*_	0.87	0.46	1.66	0.672	0.82	0.38	1.79	0.624
Sex		Male	1.00							
		Female	0.79	0.61	1.03	**0.081**	0.75	0.53	1.06	0.101
Age			1.01	1.00	1.02	**0.126**	1.00	0.98	1.01	0.741
Education		Never went to school	1.00			**0.023**				
		Primary	0.19	1.33		0.576	0.76	0.48	1.22	0.262
		Higher education	0.64	0.42	0.96	0.031	0.87	0.54	1.41	0.587
Socioeconomic status		Most poor	1.00			**<0.001**				
		Poor	0.61	0.44	0.84	<0.001	0.76	0.53	1.09	0.139
		Less poor	0.59	0.43	0.82	<0.001	0.95	0.63	1.43	0.813
Number of people per household		Single	1.00			**0.097**				
		2–4	1.28	0.85	1.93	0.245	1.33	0.83	2.05	0.252
		> 4	1.55	1.01	2.39	0.051	1.43	0.87	2.35	0.161
Toilet facility		Flush toilet	1.00			**0.010**				
		Pit latrine	1.61	0.91	2.85	0.112	1.79	0.93	3.43	0.082
		No facility	2.61	1.32	5.18	<0.001	1.43	0.63	3.23	0.394
Toilet sharing		Private toilet	1.00			**0.012**				
		2 and 3 households	0.77	0.55	1.07	0.121	0.87	0.64	1.27	0.481
		≥ 4 households	1.19	0.85	1.67	0.314	1.14	0.75	1.74	0.542
Flooding of living area		No	1.00							
		Yes	1.99	1.49	2.66	**<0.001**	1.02	0.68	1.53	0.911
Source of drinking water		Bottle, tap, rain water	1.00			**0.101**				
		Spring	1.33	1.00	1.76	0.055	0.25	0.12	1.61	0.611
		Other	1.30	0.77	2.23	0.322	0.95	0.48	1.86	0.884
Source of bath water		Tap, rain water	1.00			**0.053**				
		Spring	1.35	1.02	1.82	0.041	1.06	0.65	1.73	0.817
		Unprotected	1.44	0.91	2.29	0.121	1.16	0.64	2.09	0.632
Bathing per week		**<** 7	1.00			**0.062**				
		7–13	0.86	0.48	1.56	0.626	1.22	0.62	2.31	0.613
		≥ 14	0.65	0.36	1.16	0.151	1.05	0.53	2.09	0.892
Hand washing	After defecation	No	1.00							
		Yes	0.92	0.69	1.22	**0.562**				
	After work	No	1.00							
		Yes	1.22	0.94	1.59	**0.133**	1.02	0.75	1.38	0.921
	Before eating	No	1.00							
		Yes	1.29	0.92	1.83	**0.141**	1.25	0.86	1.82	0.253
Hand washing per week		< 4	1.00			**0.045**				
		4–7	0.87	0.63	1.19	0.383	0.91	0.64	1.28	0.582
		≥ 8	0.62	0.42	0.93	0.010	0.75	0.49	1.16	0.212
Use soap to wash your hand		No	1.00							
		Yes	0.96	0.70	1.31	**0.715**	0.91	0.65	1.33	0.711
Deworming (month)		< 6	1.00			**0.991**				
		6–12	1.05	0.70	1.59	0.832				
		> 12	1.00	0.74	1.34	0.982				

^*§*^Parasitic infection include: *Ascaris lumbricoides*, *Trichuris trichiura*, hookworm, *Schistosoma mansoni*, and any intestinal protozoa.

*p-value and odds ratio (OR) based on likelihood ratio test of univariate logistic regression, overall p-value of the models are indicated in bold letters.

** p-value and adjusted

(a) OR based on likelihood ratio test of the multivariate regression model. The multivariate model was defined including exposure groups, sex, age, educational attainment, socioeconomic status, and number of people per household. In addition, all risk factors that had a p-value lower than 0.2 in the univariate analyses were included into the multivariate regression analysis (as indicated in the table).

*** exposure groups: *com*_*exposed*_, slum dwellers at risk of flooding along the Nakivubo wetland; *com*_*comparison*_, slum dwellers without risk of flooding at least 2 km away from the Nakivubo wetland; *farmer*, urban farmers reusing wastewater within the Nakivubo wetland; *worker*_*ww*_, workers maintaining drainage channels and operating the Bugolobi Sewage Treatment Works; *worker*_*fs*_, workers managing fecal sludge (e.g., collection at households by means of vacuum trucks).

## Discussion

We report data on prevalence and risk factors for intestinal parasitic infections in exposed adult population groups along the major wastewater and fecal sludge use system in Kampala, Uganda. Urban farmers had the highest prevalence rate and higher ORs compared to the other exposure groups for the majority of measured health outcomes. Indeed, urban farmers had a point-prevalence of intestinal parasites of 76%. Hookworm and *S*. *mansoni* were the predominant infections; 28% and 23%, respectively. We found significantly higher odds of infection, regardless of the parasite species for urban farmers compared to other population groups (adjusted OR between 1.6 and 12.9).

The high hookworm and *S*. *mansoni* prevalence in urban farmers are in line with previous reports from other studies in Africa and elsewhere in the tropics [12,13,15,41]. In addition to this already established relationship, we could show that there are considerable differences in prevalence of specific intestinal parasites between the five exposure groups. Hence, the comparison between farming and non-farming household, which has been done in previous studies, may not be sufficient to understand the occurrence of intestinal parasite infection in an urban context. Our results suggest that it is important to also take into account different occupational and non-occupational exposure groups along wastewater and fecal sludge management and use systems [19,20]. The high prevalence of hookworm in urban farmers can be explained, at least partially, with concentration of hookworm eggs (2.0 eggs/l) found in water around the Nakivubo wetland. However, low concentration of *A*. *lumbricoides* (0.2 eggs/l) and the absence of *T*. *trichiura* eggs do not correlate with the respective prevalence in the exposure groups [26]. Comparing our results with model-based prevalence predictions, we found significantly lower prevalence of soil-transmitted helminths and *S*. *mansoni* in slum dwellers. However, prevalence obtained in farmers match the model-based predictions for soil-transmitted helminths and *S*. *mansoni* and are even exceeding predicted values up to a factor five for *T*. *trichiura* and *A*. *lumbricoides* [27,28]. Hence, our findings corroborate the concept that model-based prediction for urban areas should account for environmental factors (e.g., altitude), occupation, and socioeconomic status [3]. The overall prevalence of *E*. *histolytica/E*. *dispar* and *G*. *intestinalis* among all study participants was considerably lower than in a study of rural communities along Lake Victoria[30]. Multivariate regression analyses revealed lower odds for participants who went to school and attained at least primary level. Altogether, and in contrast to recent risk assessments by means of quantitative microbial risk assessment [33], our results showed that helminth and intestinal protozoa infections are relevant and important factors to consider for further risk assessments and burden estimates.

Our study has five main limitations. First, due to its cross-sectional design, this study only reflects one point in time, i.e., the rainy season, and thus, we may underestimate seasonal patterns of intestinal parasite infections and other diseases that may give rise to diarrhea such as seasonal outbreaks of cholera and typhoid [42–44]. Second, a single stool sample was examined. The reported point-prevalence of helminth and intestinal protozoa infections are thus underestimated [45]. In order to increase the sensitivity and deepen our understanding of the diversity of pathogenic organisms, other methods (e.g., polymerase chain reaction or metagenomics) need to be considered in future investigations [46]. Third, due to the relatively low number of workers included, the observed OR between intestinal parasitic infection and exposure variables for workers have to be interpreted with caution. Fourth, it has been shown that self-reported disease outcomes are prone to reporting bias. Hence, longitudinal monitoring of diarrhea incidence is warranted to get a more comprehensive understanding [37]. Fifth, it is widely acknowledged that school-aged children are at highest risk of soil-transmitted helminths and intestinal protozoa infection, hence, there is a need to further investigate school-aged children in this settings [47].

Despite these limitations, our findings raise a number of important issues. First, urban farmers, living within marginalized slum communities, appear to be most exposed and vulnerable for intestinal parasites and might contribute to their transmission in urban environments. Second, we did not find any significant positive association between current deworming practices and intestinal parasitic infection. However, our findings with hookworm and *S*. *mansoni* prevalence rates in excess of 20% in urban farmers call for preventive chemotherapy, at least in this population group. Third, we observed differences between self-reported signs and symptoms and actual prevalence of intestinal parasitic infections measured in the different exposure groups, and hence other factors (e.g., toxic chemicals, pantothenic viruses, and bacteria) might have considerable implications in these exposure groups.

Taken together, our results show that urban farmers are especially vulnerable and may play an important role in the transmission of soil-transmitted helminths and *S*. *mansoni*, most likely through contamination of their living and working environment. We recommend longitudinal monitoring of parasitic infections and diarrhea alongside with targeted interventions in exposed population groups. Altogether, this calls for increased public health protection measures for urban farmers and marginalized communities and integrated sanitation safety planning at city level.

## Supporting Information

S1 Checklist CONSORT checklist. (DOCX)

S2 Checklist STROBE checklist. (DOC)

S1 Table Water, sanitation, and hygiene (WASH) specific risk factors and risk factors related to the occupation of workers and farmers. (DOCX)

S2 Table Univariate and multivariate regression models determining association between health outcomes of interest and risk/confounding factors. (DOCX)

S1 Protocol Study protocol. (DOCX)
