# Supplementary material for: Risk of Intestinal Parasitic Infections in People with Different Exposures to Wastewater and Fecal Sludge in Kampala, Uganda: A Cross-Sectional Study
Source: PLoS Negl Trop Dis. 2016 Mar 3;10(3):e0004469. doi: 10.1371/journal.pntd.0004469 (PMC4777287; doi:10.1371/journal.pntd.0004469)
Supplement: S1 Checklist — (DOCX) [file pntd.0004469.s001.docx]

**S1 Checklist CONSORT statement** for: Risk of Intestinal Parasitic Infections in People with Different Exposures to Wastewater and Fecal Sludge in Kampala, Uganda: A Cross-Sectional Study

| **Item** | **Description** | **Reported on line number** |
| --- | --- | --- |
| Title | Identification of the study as randomized | **1** |
| Authors | Contact details for the corresponding author | **1** |
| Trial design | Description of the trial design (e.g. parallel, cluster, non-inferiority) | **6** |
| Methods |  | **6 and 8** |
| Participants | Eligibility criteria for participants and the settings where the data were collected | **6 and 8** |
| Interventions | Interventions intended for each group | **6** |
| Objective | Specific objective or hypothesis | **5** |
| Outcome | Clearly defined primary outcome for this report | **8** |
| Randomization | How participants were allocated to interventions | **7** |
| Blinding (masking) | Whether or not participants, care givers, and those assessing the outcomes were blinded to group assignment | **7** |
| Results |  |  |
| Numbers randomized | Number of participants randomized to each group | **9** |
| Recruitment | Trial status | **9** |
| Numbers analysed | Number of participants analysed in each group | **9** |
| Outcome | For the primary outcome, a result for each group and the estimated effect size and its precision | **9 to 16** |
| Harms | Important adverse events or side effects | **No side effects** |
| Conclusions | General interpretation of the results | **18** |
| Trial registration | Registration number and name of trial register | **5** |
| Funding | Source of funding | **Provided in the submission** |
