# Supplementary material for: Risk of Intestinal Parasitic Infections in People with Different Exposures to Wastewater and Fecal Sludge in Kampala, Uganda: A Cross-Sectional Study
Source: PLoS Negl Trop Dis. 2016 Mar 3;10(3):e0004469. doi: 10.1371/journal.pntd.0004469 (PMC4777287; doi:10.1371/journal.pntd.0004469)
Supplement: S2 Checklist — (DOC) [file pntd.0004469.s002.doc]

**S2 Checklist STROBE Statement** for: Risk of Intestinal Parasitic Infections in People with Different Exposures to Wastewater and Fecal Sludge in Kampala, Uganda: A Cross-Sectional Study

|  | Item No | Recommendation |
| --- | --- | --- |
| **Title and abstract** | 1 | (*a*) Indicate the study’s design with a commonly used term in the title or the abstract  **Page 1 and 2** |
|  |  | (*b*) Provide in the abstract an informative and balanced summary of what was done and what was found  **Page 1 and 4** |
| Introduction | | |
| Background/rationale | 2 | Explain the scientific background and rationale for the investigation being reported  **Page 6** |
| Objectives | 3 | State specific objectives, including any prespecified hypotheses  **Page 6** |
| Methods | | |
| Study design | 4 | Present key elements of study design early in the paper  **Page 2 and 7** |
| Setting | 5 | Describe the setting, locations, and relevant dates, including periods of recruitment, exposure, follow-up, and data collection  **Page 7 and 8** |
| Participants | 6 | (*a*) Give the eligibility criteria, and the sources and methods of selection of participants **Page 8** |
| Variables | 7 | Clearly define all outcomes, exposures, predictors, potential confounders, and effect modifiers. Give diagnostic criteria, if applicable **Page 9 and 10** |
| Data sources/ measurement | 8* | For each variable of interest, give sources of data and details of methods of assessment (measurement). Describe comparability of assessment methods if there is more than one group **Page 9 and 10** |
| Bias | 9 | Describe any efforts to address potential sources of bias **Page 9 and 10** |
| Study size | 10 | Explain how the study size was arrived at **Page 8** |
| Quantitative variables | 11 | Explain how quantitative variables were handled in the analyses. If applicable, describe which groupings were chosen and why **Page 9 and 10** |
| Statistical methods | 12 | (*a*) Describe all statistical methods, including those used to control for confounding  **Page 9 and 10** |
|  |  | (*b*) Describe any methods used to examine subgroups and interactions  **Page 9 and 10** |
|  |  | (*c*) Explain how missing data were addressed  **Page 9 and 10** |
|  |  | (*d*) If applicable, describe analytical methods taking account of sampling strategy  **Page 8** |
|  |  | (*e*) Describe any sensitivity analyses  **Not done** |
| Results | | |
| Participants | 13* | (a) Report numbers of individuals at each stage of study—eg numbers potentially eligible, examined for eligibility, confirmed eligible, included in the study, completing follow-up, and analysed **Page 10** |
|  |  | (b) Give reasons for non-participation at each stage **Page 10** |
|  |  | (c) Consider use of a flow diagram **Page 10** |
| Descriptive data | 14* | (a) Give characteristics of study participants (eg demographic, clinical, social) and information on exposures and potential confounders  **Page 12** |
|  |  | (b) Indicate number of participants with missing data for each variable of interest **Page 10** |
| Outcome data | 15* | Report numbers of outcome events or summary measures **Page 15 and 16** |
| Main results | 16 | (*a*) Give unadjusted estimates and, if applicable, confounder-adjusted estimates and their precision (eg, 95% confidence interval). Make clear which confounders were adjusted for and why they were included **between Page 12 and 18** |
|  |  | (*b*) Report category boundaries when continuous variables were categorized **between Page 12 and 18** |
|  |  | (*c*) If relevant, consider translating estimates of relative risk into absolute risk for a meaningful time period **not relevant for this paper** |
| Other analyses | 17 | Report other analyses done—eg analyses of subgroups and interactions, and sensitivity analyses **Page 9 and 10** |
| Discussion | | |
| Key results | 18 | Summarise key results with reference to study objectives **Page 19** |
| Limitations | 19 | Discuss limitations of the study, taking into account sources of potential bias or imprecision. Discuss both direction and magnitude of any potential bias **Page 19 and 20** |
| Interpretation | 20 | Give a cautious overall interpretation of results considering objectives, limitations, multiplicity of analyses, results from similar studies, and other relevant evidence **Page 19 and 20** |
| Generalisability | 21 | Discuss the generalisability (external validity) of the study results **Page 19 and 20** |
| Other information | | |
| Funding | 22 | Give the source of funding and the role of the funders for the present study and, if applicable, for the original study on which the present article is based **separate document handed in to PLoS NTD** |

*Give information separately for exposed and unexposed groups.

**Note:** An Explanation and Elaboration article discusses each checklist item and gives methodological background and published examples of transparent reporting. The STROBE checklist is best used in conjunction with this article (freely available on the Web sites of PLoS Medicine at http://www.plosmedicine.org/, Annals of Internal Medicine at http://www.annals.org/, and Epidemiology at http://www.epidem.com/). Information on the STROBE Initiative is available at www.strobe-statement.org.
