# Supplementary material for: Risk of Intestinal Parasitic Infections in People with Different Exposures to Wastewater and Fecal Sludge in Kampala, Uganda: A Cross-Sectional Study
Source: PLoS Negl Trop Dis. 2016 Mar 3;10(3):e0004469. doi: 10.1371/journal.pntd.0004469 (PMC4777287; doi:10.1371/journal.pntd.0004469)
Supplement: S1 Table — (DOCX) [file pntd.0004469.s003.docx]

**S1 Table. Water, sanitation and hygiene (WASH) specific risk factors and risk factors related to the occupation of workers and farmers**

**S1A** Water, sanitation and hygiene (WASH) specific risk factors of the participants enrolled in the cross-sectional survey in Kampala, stratified by five exposure groups.

| **Water, sanitation and hygiene risk factors** | ***com _comparison_***^*^ | | ***com* *_exposed_***^*^ | | ***farmer***^*^ | | ***worker _fs_*** ^*^ | | ***worker* *_ww_***^*^ | | **Difference (χ²)** |
| --- | --- | --- | --- | --- | --- | --- | --- | --- | --- | --- | --- |
|  | n=331 | | n=229 | | n=245 | | n=67 | | n=43 | |  |
|  | **n** | **%** | **n** | **%** | **n** | **%** | **n** | **%** | **n** | **%** | **p-value** |
| **Exposure to potentially contaminated water while** | | | |  |  |  |  |  |  |  |  |
| Flooding of living area | 5 | 1.5 | 108 | 47.1 | 157 | 64.1 | 6 | 9.0 | 14 | 32.6 | <0.001 |
| Swimming in  Lake Victoria | 0 | 0 | 0 | 0 | 1 | 0.4 | 1 | 1.5 | 1 | 2.3 | 0.043 |
| **Household with latrine** | 214 | 64.7 | 112 | 48.9 | 137 | 55.9 | 63 | 94.0 | 41 | 95.4 | <0.001 |
| **What kind of your toilet do you use** | | | | |  |  |  |  |  |  |  |
| Flush toilet | 8 | 2.4 | 3 | 1.31 | 19 | 7.8 | 10 | 14.9 | 12 | 27.9 |  |
| VIP latrine | 92 | 27.8 | 110 | 48.0 | 74 | 30.2 | 39 | 58.2 | 23 | 53.5 |  |
| Traditional pit late | 222 | 67.1 | 77 | 33.6 | 100 | 40.8 | 17 | 25.4 | 7 | 16.3 |  |
| No facility | 4 | 1.2 | 2 | 0.9 | 8 | 3.3 | 0 | 0 | 0 | 0 |  |
| Other places | 5 | 1.5 | 37 | 16.1 | 44 | 18.0 | 1 | 1.5 | 1 | 2.3 | <0.001 |
| **With how many households do you share your toilet** | | | | |  |  |  |  |  |  |  |
| Private toilet | 84 | 25.4 | 43 | 18.7 | 76 | 31.0 | 17 | 25.4 | 20 | 46.5 |  |
| 1-5 households | 77 | 23.3 | 34 | 14.8 | 37 | 15.1 | 21 | 31.3 | 10 | 23.3 |  |
| 5-11 households | 79 | 23.9 | 20 | 8.7 | 36 | 14.7 | 19 | 28.4 | 11 | 25.6 |  |
| ≥ 11 public toilet | 91 | 27.5 | 132 | 55.0 | 96 | 39.2 | 10 | 14.9 | 2 | 4.7 | <0.001 |
| **Handwashing** |  |  |  |  |  |  |  |  |  |  |  |
| After defecation | 280 | 84.6 | 182 | 79.5 | 139 | 56.7 | 28 | 41.8 | 22 | 51.2 | <0.001 |
| Before starting work | 32 | 9.7 | 28 | 12.2 | 15 | 6.1 | 7 | 10.5 | 9 | 20.9 | 0.025 |
| After work | 135 | 40.8 | 98 | 42.8 | 178 | 72.7 | 48 | 71.6 | 29 | 67.4 | <0.001 |
| Before eating | 264 | 79.8 | 186 | 81.2 | 207 | 84.5 | 51 | 76.1 | 35 | 81.4 | 0.505 |
| After eating | 266 | 80.4 | 181 | 79.0 | 168 | 68.6 | 37 | 55.2 | 30 | 69.8 | <0.001 |
| **Do you use soap to wash your hand** | 221 | 66.8 | 180 | 78.6 | 204 | 83.3 | 64 | 95.5 | 38 | 88.4 | <0.001 |
| **Household with tap water** | 121 | 36.6 | 93 | 40.6 | 72 | 29.4 | 46 | 68.7 | 24 | 55.8 | <0.001 |
| **Source of drinking water** |  |  |  |  |  |  |  |  |  |  |  |
| Bottled water | 62 | 18.7 | 35 | 15.3 | 16 | 6.5 | 44 | 65.7 | 23 | 53.5 | <0.001 |
| Tap water | 276 | 83.4 | 129 | 56.3 | 144 | 58.8 | 48 | 71.6 | 33 | 76.7 | <0.001 |
| Rain water | 28 | 8.5 | 12 | 5.2 | 19 | 7.8 | 6 | 9.0 | 7 | 16.3 | 0.156 |
| Bore whole water | 4 | 1.2 | 1 | 0.4 | 5 | 2.0 | 1 | 1.5 | 0 | 0 | 0.533 |
| Spring water | 98 | 29.6 | 115 | 50.2 | 92 | 37.6 | 10 | 14.9 | 8 | 18.6 | <0.001 |
| Well water | 6 | 1.8 | 2 | 0.9 | 19 | 7.8 | 2 | 2.9 | 5 | 11.6 | <0.001 |
| Water from  Lake Victoria | 0 | 0 | 0 | 0 | 1 | 0.4 | 1 | 1.5 | 0 | 0 | 0.152 |
| **Eating or potential contaminated food** | | | | | |  |  |  |  |  |  |
| Vegetables | 135 | 40.8 | 103 | 44.9 | 107 | 43.7 | 43 | 64.2 | 37 | 86.1 | <0.001 |
| Root crops | 144 | 43.5 | 173 | 75.6 | 234 | 95.5 | 45 | 67.2 | 35 | 81.4 | <0.001 |
| Sugar cane | 46 | 13.9 | 98 | 42.8 | 134 | 54.7 | 20 | 29.9 | 17 | 39.5 | <0.001 |
| Yam roots | 68 | 20.5 | 115 | 50.2 | 189 | 77.1 | 20 | 29.9 | 16 | 37.2 | <0.001 |
| Cassava roots | 84 | 25.4 | 95 | 41.5 | 129 | 52.7 | 36 | 53.7 | 28 | 65.1 | <0.001 |
| **Preventive chemotherapy taken against soil transmitted helminth infections (month)** | | | | | | | |  |  |  |  |
| < 3 | 55 | 16.6 | 38 | 16.6 | 48 | 19.6 | 31 | 46.3 | 10 | 23.3 | <0.001 |
| < 6 | 93 | 28.1 | 59 | 25.8 | 66 | 26.9 | 40 | 59.7 | 11 | 25.6 | <0.001 |
| < 12 | 135 | 40.8 | 102 | 44.5 | 101 | 41.2 | 47 | 70.2 | 24 | 55.8 | <0.001 |
| ≥ 12 | 331 | 100 | 229 | 100 | 245 | 100 | 62 | 92.5 | 42 | 97.7 | 0.032 |

^*^*“com* *_exposed_”*, slum dwellers at risk of flooding along the Nakivubo wetland; *“com* *_comparison_”*, slum dwellers without risk of flooding at least 2 km away from the Nakivubo wetland; *“farmer”*, urban farmers reusing wastewater within the Nakivubo wetland; *“worker* *_ww_”*, workers maintaining drainage channels and operating the Bugolobi Sewage Treatment Works; *“worker _fs_”*, workers managing fecal sludge (e.g., collection at households by means of vacuum trucks).

**S1B** Risk factors related to the occupation of workers and farmers enrolled in the cross-sectional survey in Kampala.

|  | ***farmer***^*^ | | ***worker _fs_*** ^*^ | | ***worker* *_ww_***^*^ | | **Difference**  **(χ²)** |
| --- | --- | --- | --- | --- | --- | --- | --- |
| **Occupational risk factors** | n=245 | | n=67 | | n=43 | |  |
|  | **n** | **%** | **n** | **%** | **n** | **%** | **p-value** |
| **Formally employed** | 10 | 4.1 | 65 | 97.0 | 42 | 97.7 | <0.001 |
| **Months worked in the current job (month**) | |  |  |  |  |  |  |
| 1-12 | 91 | 37.1 | 38 | 56.7 | 15 | 34.9 |  |
| 12-36 | 62 | 25.3 | 18 | 26.9 | 11 | 25.6 |  |
| ≥ 36 | 91 | 37.1 | 11 | 16.4 | 17 | 39.5 | <0.001 |
| **Hours worked per week (hours)** |  |  |  |  |  |  |  |
| 1-20 | 32 | 13.1 | 1 | 1.5 | 0 | 0.0 |  |
| 20-40 | 132 | 54.7 | 2 | 3.0 | 1 | 2.3 |  |
| 40-60 | 60 | 24.5 | 19 | 28.4 | 24 | 55.8 |  |
| ≥ 60 | 19 | 32.2 | 64 | 67.2 | 18 | 41.9 | <0.001 |
| **Use of personal protective equipment** | | |  |  |  |  |  |
| Gloves | 10 | 4.1 | 59 | 88.1 | 35 | 81.4 | <0.001 |
| Uniform | 8 | 3.3 | 39 | 58.2 | 32 | 74.4 | <0.001 |
| Long sleeves | 61 | 24.9 | 4 | 6.0 | 8 | 18.6 | <0.001 |
| Rubber boots | 121 | 49.4 | 42 | 62.7 | 35 | 81.4 | <0.001 |
| Appropriate shoes | 38 | 15.5 | 24 | 35.8 | 23 | 53.5 | <0.001 |
| Working tools | 59 | 24.1 | 2 | 3.0 | 2 | 3.0 | <0.001 |

^*^*“farmer”*, urban farmers reusing wastewater within the Nakivubo wetland; *“worker* *_ww_”*, workers maintaining drainage channels and operating the Bugolobi Sewage Treatment Works; *“worker _fs_”*, workers managing fecal sludge (e.g., collection at households by means of vacuum trucks).
