# Supplementary material for: Risk of Intestinal Parasitic Infections in People with Different Exposures to Wastewater and Fecal Sludge in Kampala, Uganda: A Cross-Sectional Study
Source: PLoS Negl Trop Dis. 2016 Mar 3;10(3):e0004469. doi: 10.1371/journal.pntd.0004469 (PMC4777287; doi:10.1371/journal.pntd.0004469)
Supplement: S2 Table — (DOCX) [file pntd.0004469.s004.docx]

**S2 Table. Univariate and multivariate regression models determining association between health outcomes of interest and risk/confounding factors**

**S2A** Results of univariate and the multivariate logistic regression analysis for helminth infection infections in a cross-sectional survey done in late 2013 in Kampala^§^

| Any helminth infection  N=915 / N(cases)=247 | | |  | **Univariate logistic regression*** | | | |  | **Multivariate logistic regression**** | | | |
| --- | --- | --- | --- | --- | --- | --- | --- | --- | --- | --- | --- | --- |
|  |  |  |  | OR | 95% CI | | p-value |  | aOR | 95% CI | | p-value |
| Exposure group*** | | *com _comparison_* |  | 1 |  |  | **<0·001** |  | 1 |  |  | **<0·001** |
|  |  | *com* *_exposed_* |  | 1·31 | 0·8 | 2·0 | 0·241 |  | 1·04 | 0·62 | 1·75 | 0·883 |
|  |  | *farmer* |  | 6·48 | 4·4 | 9·6 | <0·001 |  | 4·85 | 2·83 | 8·33 | <0·001 |
|  |  | *worker _fs_* |  | 0·73 | 0·3 | 1·6 | 0·454 |  | 0·54 | 0·22 | 1·32 | 0·184 |
|  |  | *worker* *_ww_* |  | 1·24 | 0·5 | 2·8 | 0·614 |  | 0·83 | 0·31 | 2·20 | 0·711 |
| Sex | | Male |  | 1 |  |  |  |  |  |  |  |  |
|  |  | Female |  | 0·55 | 0·4 | 0·7 | **<0·001** |  | 0·58 | 0·40 | 0·85 | 0·011 |
| Age | |  |  | 1·02 | 1·0 | 1·0 | **0·012** |  | 1·00 | 0·99 | 1·02 | 0·952 |
| Education | | Never went to school |  | 1 |  |  | **<0·001** |  |  |  |  |  |
|  |  | Primary |  | 1·47 | 0·9 | 2·3 | 0·111 |  | 1·34 | 0·79 | 2·27 | 0·281 |
|  |  | Higher education |  | 0·75 | 0·5 | 1·2 | 0·248 |  | 1·15 | 0·65 | 2·03 | 0·632 |
| Socio-economic status | | Most poor |  | 1 |  |  | **0·032** |  |  |  |  |  |
|  |  | Poor |  | 0·72 | 0·5 | 1·0 | 0·072 |  | 0·98 | 0·65 | 1·50 | 0·931 |
|  |  | Less poor |  | 0·61 | 0·4 | 0·9 | 0·011 |  | 1·16 | 0·72 | 1·86 | 0·544 |
| Number of people per household | | Single |  | 1 |  |  | **0·061** |  |  |  |  |  |
|  |  | 2 to 4 |  | 1·19 | 0·7 | 1·9 | 0·482 |  | 1·24 | 0·71 | 2·16 | 0·453 |
|  |  | > 4 |  | 1·59 | 1·0 | 2·6 | 0·071 |  | 1·36 | 0·75 | 2·46 | 0·322 |
| Toilet facility | | Flush toilet |  | 1 |  |  | **0·027** |  |  |  |  |  |
|  |  | Pit latrine |  | 1·14 | 0·6 | 2·2 | 0·705 |  | 1·50 | 0·70 | 3·22 | 0·302 |
|  |  | No facility |  | 2·15 | 1·0 | 4·6 | 0·057 |  | 1·39 | 0·55 | 3·52 | 0·492 |
| Toilet sharing | | Private toilet |  | 1 |  |  | **0·031** |  |  |  |  |  |
|  |  | 2 and 3 households |  | 0·68 | 0·5 | 1·0 | 0·051 |  | 0·82 | 0·53 | 1·27 | 0·376 |
|  |  | ≥ 4 households |  | 1·07 | 0·7 | 1·5 | 0·703 |  | 0·90 | 0·55 | 1·48 | 0·691 |
| Flooding of living area | | No |  | 1 |  |  |  |  |  |  |  |  |
|  |  | Yes |  | 2·69 | 1·99 | 3·66 | **<0·001** |  | 1·38 | 0·88 | 2·15 | 0·162 |
| Source of drinking water | | Bottle, Tab, rain |  | 1 |  |  | **0·023** |  |  |  |  |  |
|  |  | Spring |  | 1·53 | 1·12 | 2·09 | 0·012 |  | 1·12 | 0·62 | 2·01 | 0·714 |
|  |  | Other |  | 1·23 | 0·7 | 2·2 | 0·481 |  | 0·62 | 0·28 | 1·37 | 0·245 |
| Source of bath water | | Tab, rain water |  | 1 |  |  | **<0·001** |  |  |  |  |  |
|  |  | Spring |  | 1·54 | 1·1 | 2·1 | 0·011 |  | 1·19 | 0·66 | 2·17 | 0·561 |
|  |  | Unprotected |  | 1·57 | 1·0 | 2·6 | 0·080 |  | 1·40 | 0·71 | 2·77 | 0·330 |
| Bathing per week | | **<** 7 |  | 1 |  |  | **0·632** |  |  |  |  |  |
|  |  | ≥ 7 to < 14 |  | 0·71 | 0·4 | 1·3 | 0·277 |  | 1·06 | 0·53 | 2·15 | 0·869 |
|  |  | > 14 |  | 0·53 | 0·3 | 1·0 | 0·048 |  | 1·07 | 0·51 | 2·26 | 0·851 |
| Hand washing | After defecation | No |  | 1 |  |  |  |  |  |  |  |  |
|  |  | Yes |  | 0·66 | 0·48 | 0·90 | **0·010** |  | 0·90 | 0·61 | 1·33 | 0.602 |
|  | After work | No |  | 1 |  |  |  |  |  |  |  |  |
|  |  | Yes |  | 1·28 | 1·0 | 1·7 | **0·101** |  | 0·79 | 0·55 | 1·14 | 0.218 |
|  | Before eating | No |  | 1 |  |  |  |  |  |  |  |  |
|  |  | Yes |  | 1·23 | 0·8 | 1·8 | **0·292** |  |  |  |  |  |
| Hand washing per week | | < 4 |  | 1 |  |  | **0·411** |  |  |  |  |  |
|  |  | < 8 |  | 0·97 | 0·7 | 1·4 | 0·853 |  | 1·12 | 0·75 | 1·65 | 0·584 |
|  |  | ≥ 8 |  | 0·62 | 0·4 | 1·0 | 0·030 |  | 0·90 | 0·53 | 1·51 | 0·691 |
| Use soap to wash your hand | | No |  | 1 |  |  |  |  |  |  |  |  |
|  |  | Yes |  | 0·84 | 0·6 | 1·2 | **0·345** |  |  |  |  |  |
| Deworming (month) | | < 6 |  | 1 |  |  | **0·692** |  |  |  |  |  |
|  |  | 6-12 |  | 1·21 | 0·8 | 1·9 | 0·402 |  |  |  |  |  |
|  |  | > 12 |  | 1·08 | 0·8 | 1·5 | 0·641 |  |  |  |  |  |

*^§^*Helminth infection include: *Ascaris lumbricoides, Trichuris trichiura*, hookworm, *Schistosoma mansoni.* *p-value and odds ratio (OR) based on likelihood ratio test of univariate logistic regression, overall p-value of the models are indicated in bold letters. ** p-value and adjusted (a) OR based on likelihood ratio test of the multivariate regression model. The multivariate model was defined including exposure groups, sex, age, educational attainment, socioeconomic status, and number of people per household. In addition, all risk factors that had a p-value lower than 0.2 in the univariate analyses were included into the multivariate regression analysis (as indicated in the table). *** exposure groups: *com _exposed_*, slum dwellers at risk of flooding along the Nakivubo wetland; *com _comparison_*, slum dwellers without risk of flooding at least 2 km away from the Nakivubo wetland; *farmer*, urban farmers reusing wastewater within the Nakivubo wetland; *worker _ww_*, workers maintaining drainage channels and operating the Bugolobi Sewage Treatment Works; *worker _fs_*, workers managing fecal sludge (e.g., collection at households by means of vacuum trucks).

**S2B** Results of univariate and the multivariate logistic regression analysis for soil-transmitted helminth infection in a cross-sectional survey done in late 2013 in Kampala^§^

| Soil-transmitted helminth infection  N(total)=915 / N(cases)= 187 | | |  | Univariate logistic regression* | | | |  | Multivariate logistic regression** | | | |
| --- | --- | --- | --- | --- | --- | --- | --- | --- | --- | --- | --- | --- |
|  |  |  |  | OR | 95% CI | | p-value |  | aOR | 95% CI | | p-value |
| Exposure group*** | | *com _comparison_* |  | 1 |  |  | **<0·001** |  | 1 |  |  | **<0·001** |
|  |  | *com* *_exposed_* |  | 0·78 | 0·4 | 1·4 | 0·381 |  | 0·58 | 0·31 | 1·09 | 0·091 |
|  |  | *farmer* |  | 6·78 | 4·4 | 10·3 | <0·001 |  | 4·66 | 2·58 | 8·41 | <0·001 |
|  |  | *worker _fs_* |  | 0·61 | 0·2 | 1·6 | 0·322 |  | 0·41 | 0·14 | 1·19 | 0·103 |
|  |  | *worker* *_ww_* |  | 1·48 | 0·6 | 3·6 | 0·381 |  | 1·07 | 0·38 | 3·06 | 0·893 |
| Sex | | Male |  |  |  |  |  |  |  |  |  |  |
|  |  | Female |  | 0·57 | 0·4 | 0·8 | **<0·001** |  | 0·67 | 0·44 | 1·02 | 0·064 |
| Age | |  |  | 0·02 | 0·0 | 0·0 | **<0·001** |  | 1·01 | 0·99 | 1·02 | 0·511 |
| Education | | Never went to school |  |  |  |  | **<0·001** |  |  |  |  |  |
|  |  | Primary |  | 1·27 | 0·8 | 2·1 | 0·333 |  | 1·28 | 0·72 | 2·27 | 0·402 |
|  |  | Higher education |  | 0·56 | 0·3 | 0·9 | 0·032 |  | 0·94 | 0·50 | 1·75 | 0·841 |
| Socio-economic status | | Most poor |  |  |  |  | **0·05** |  |  |  |  |  |
|  |  | Poor |  | 0·69 | 0·5 | 1·0 | 0·061 |  | 1·01 | 0·63 | 1·61 | 0·985 |
|  |  | Less poor |  | 0·62 | 0·4 | 0·9 | 0·027 |  | 1·44 | 0·85 | 2·43 | 0·183 |
| Number of people per household | | Single |  |  |  |  | **0·19** |  |  |  |  |  |
|  |  | 2 to 4 |  | 1·18 | 0·7 | 2·0 | 0·540 |  | 1·25 | 0·67 | 2·32 | 0·491 |
|  |  | > 4 |  | 1·54 | 0·9 | 2·7 | 0·138 |  | 1·21 | 0·62 | 2·37 | 0·574 |
| Toilet facility | | Flush toilet |  |  |  |  | **<0·001** |  |  |  |  |  |
|  |  | Pit latrine |  | 1·26 | 0·6 | 2·7 | 0·561 |  | 1·91 | 0·80 | 4·57 | 0·151 |
|  |  | No facility |  | 3·27 | 1·4 | 7·7 | 0·011 |  | 3·14 | 1·11 | 8·88 | 0·032 |
| Toilet sharing | | Private toilet |  |  |  |  | **0·03** |  |  |  |  |  |
|  |  | 2 and 3 households |  | 0·70 | 0·5 | 1·1 | 0·101 |  | 0·91 | 0·56 | 1·49 | 0·721 |
|  |  | ≥ 4 households |  | 1·17 | 0·8 | 1·7 | 0·457 |  | 1·09 | 0·62 | 1·89 | 0·776 |
| Flooding of living area | | No |  |  |  |  |  |  |  |  |  |  |
|  |  | Yes |  | 2·76 | 1·98 | 3·84 | **<0·001** |  | 1·27 | 0·76 | 2·13 | 0·367 |
| Source of drinking water | | Bottle, Tab, rain |  |  |  |  | **0·532** |  |  |  |  |  |
|  |  | Spring |  | 1·20 | 0·85 | 1·69 | 0·302 |  |  |  |  |  |
|  |  | Other |  | 1·15 | 0·6 | 2·2 | 0·673 |  |  |  |  |  |
| Source of bath water | | Tab, rain water |  |  |  |  | **0·081** |  |  |  |  |  |
|  |  | Spring |  | 1·30 | 0·9 | 1·8 | 0·142 |  | 0·97 | 0·63 | 1·48 | 0·887 |
|  |  | Unprotected |  | 1·70 | 1·0 | 2·9 | 0·053 |  | 1·29 | 0·72 | 2·31 | 0·403 |
| Bathing per week | | **<** 7 |  |  |  |  | **0·04** |  |  |  |  |  |
|  |  | ≥ 7 to < 14 |  | 0·73 | 0·4 | 1·4 | 0·331 |  | 1·07 | 0·51 | 2·25 | 0·872 |
|  |  | > 14 |  | 0·51 | 0·3 | 1·0 | 0·050 |  | 1·12 | 0·50 | 2·48 | 0·789 |
| Hand washing | After defecation | No |  |  |  |  |  |  |  |  |  |  |
|  |  | Yes |  | 0·55 | 0·39 | 0·77 | **<0·001** |  | 0·74 | 0·49 | 1·13 | 0.171 |
|  | After work | No |  |  |  |  |  |  |  |  |  |  |
|  |  | Yes |  | 1·28 | 0·9 | 1·8 | **0·143** |  | 0·73 | 0·48 | 1·09 | 0.121 |
|  | Before eating | No |  |  |  |  |  |  |  |  |  |  |
|  |  | Yes |  | 1·30 | 0·84 | 2·02 | **0·231** |  |  |  |  |  |
| Hand washing per week | | < 4 |  |  |  |  | **0·010** |  |  |  |  |  |
|  |  | < 8 |  | 0·95 | 0·7 | 1·4 | 0·802 |  | 1·23 | 0·80 | 1·89 | 0·341 |
|  |  | ≥ 8 |  | 0·53 | 0·3 | 0·9 | 0·011 |  | 0·80 | 0·44 | 1·45 | 0·462 |
| Use soap to wash your hand | | No |  |  |  |  |  |  |  |  |  |  |
|  |  | Yes |  | 0·84 | 0·6 | 1·2 | **0·381** |  |  |  |  |  |
| Deworming (month) | | < 6 |  |  |  |  | **0·321** |  |  |  |  |  |
|  |  | 6-12 |  | 0·99 | 0·6 | 1·7 | 0·982 |  |  |  |  |  |
|  |  | > 12 |  | 1·10 | 0·8 | 1·6 | 0·625 |  |  |  |  |  |

*^§^*Soil-transmitted helminth infection include: *Ascaris lumbricoides, Trichuris trichiura* and hookworm*.* *p-value and odds ratio (OR) based on likelihood ratio test of univariate logistic regression, overall p-value of the models are indicated in bold letters. ** p-value and adjusted (a) OR based on likelihood ratio test of the multivariate regression model. The multivariate model was defined including exposure groups, sex, age, educational attainment, socioeconomic status, and number of people per household. In addition, all risk factors that had a p-value lower than 0.2 in the univariate analyses were included into the multivariate regression analysis (as indicated in the table). *** exposure groups: *com _exposed_*, slum dwellers at risk of flooding along the Nakivubo wetland; *com _comparison_*, slum dwellers without risk of flooding at least 2 km away from the Nakivubo wetland; *farmer*, urban farmers reusing wastewater within the Nakivubo wetland; *worker _ww_*, workers maintaining drainage channels and operating the Bugolobi Sewage Treatment Works; *worker _fs_*, workers managing fecal sludge (e.g., collection at households by means of vacuum trucks).

**S2C** Results of univariate and the multivariate logistic regression analysis for any intestinal protozoa infections in a cross-sectional survey done in late 2013 in Kampala

| Intestinal protozoa infection  N(total)=915 / N(cases)= 365 | | |  | Univariate logistic regression* | | | |  | Multivariate logistic regression** | | | |
| --- | --- | --- | --- | --- | --- | --- | --- | --- | --- | --- | --- | --- |
|  |  |  |  | OR | 95% CI | | p-value |  | aOR | 95% CI | | p-value |
| Exposure group*** | | *com _comparison_* |  | 1 |  |  | **<0·001** |  | 1 |  |  | **0·031** |
|  |  | *com* *_exposed_* |  | 1·39 | 1·0 | 2·0 | 0·061 |  | 1·33 | 0·92 | 1·92 | 0·132 |
|  |  | *farmer* |  | 1·70 | 1·2 | 2·4 | <0·001 |  | 1·63 | 1·10 | 2·39 | 0·013 |
|  |  | *worker _fs_* |  | 0·67 | 0·4 | 1·2 | 0·187 |  | 0·71 | 0·37 | 1·36 | 0·303 |
|  |  | *worker* *_ww_* |  | 0·79 | 0·4 | 1·6 | 0·518 |  | 0·76 | 0·35 | 1·65 | 0·498 |
| Sex | | Male |  | 1 |  |  |  |  |  |  |  |  |
|  |  | Female |  | 1·19 | 0·9 | 1·6 | **0·202** |  | 1·05 | 0·75 | 1·46 | 0·786 |
| Age | |  |  | 0·00 | 0·0 | 0·0 | **0·705** |  | 0·99 | 0·98 | 1·00 | 0·215 |
| Education | | Never went to school |  | 1 |  |  | **0·043** |  |  |  |  |  |
|  |  | Primary |  | 0·66 | 0·4 | 1·0 | 0·052 |  | 0·64 | 0·41 | 0·99 | 0·050 |
|  |  | Higher education |  | 0·59 | 0·4 | 0·9 | 0·010 |  | 0·69 | 0·44 | 1·09 | 0·114 |
| Socio-economic status | | Most poor |  | 1 |  |  | **0·074** |  |  |  |  |  |
|  |  | Poor |  | 0·70 | 0·5 | 1·0 | 0·033 |  | 0·78 | 0·55 | 1·10 | 0·165 |
|  |  | Less poor |  | 0·75 | 0·5 | 1·0 | 0·087 |  | 0·92 | 0·63 | 1·35 | 0·675 |
| Number of people per household | | Single |  | 1 |  |  | **0·165** |  |  |  |  |  |
|  |  | 2 to 4 |  | 1·22 | 0·8 | 1·9 | 0·366 |  | 1·19 | 0·76 | 1·86 | 0·452 |
|  |  | > 4 |  | 1·49 | 1·0 | 2·3 | 0·087 |  | 1·43 | 0·88 | 2·32 | 0·151 |
| Toilet facility | | Flush toilet |  | 1 |  |  | **0·883** |  |  |  |  |  |
|  |  | Pit latrine |  | 1·16 | 0·6 | 2·1 | 0·612 |  |  |  |  |  |
|  |  | No facility |  | 1·17 | 0·6 | 2·3 | 0·667 |  |  |  |  |  |
| Toilet sharing | | Private toilet |  | 1 |  |  | **0·13** |  |  |  |  |  |
|  |  | 2 and 3 households |  | 0·75 | 0·5 | 1·1 | 0·109 |  | 0·82 | 0·58 | 1·18 | 0·296 |
|  |  | ≥ 4 households |  | 0·99 | 0·7 | 1·4 | 0·960 |  | 0·92 | 0·63 | 1·34 | 0·669 |
| Flooding of living area | | No |  | 1 |  |  |  |  |  |  |  |  |
|  |  | Yes |  | 1·17 | 0·88 | 1·55 | **0·298** |  |  |  |  |  |
| Source of drinking water | | Bottle, Tab, rain |  | 1 |  |  | **0·346** |  |  |  |  |  |
|  |  | Spring |  | 1·13 | 0·85 | 1·50 | 0·415 |  | 0·96 | 0·70 | 1·32 | 0·802 |
|  |  | Other |  | 1·43 | 0·9 | 2·4 | 0·174 |  | 1·23 | 0·78 | 1·96 | 0·381 |
| Source of bath water | | Tab, rain water |  | 1 |  |  | **0·323** |  |  |  |  |  |
|  |  | Spring |  | 1·14 | 0·9 | 1·5 | 0·361 |  | 1·02 | 0·55 | 1·87 | 0·961 |
|  |  | Unprotected |  | 1·37 | 0·9 | 2·1 | 0·171 |  | 0·95 | 0·51 | 1·77 | 0·874 |
| Bathing per week | | **<** 7 |  | 1 |  |  | **0·820** |  |  |  |  |  |
|  |  | ≥ 7 to < 14 |  | 0·93 | 0·5 | 1·7 | 0·814 |  |  |  |  |  |
|  |  | > 14 |  | 0·87 | 0·5 | 1·6 | 0·642 |  |  |  |  |  |
| Hand washing | After defecation | No |  | 1 |  |  |  |  |  |  |  |  |
|  |  | Yes |  | 1·15 | 0·86 | 1·55 | **0·358** |  |  |  |  |  |
|  | After work | No |  | 1 |  |  |  |  |  |  |  |  |
|  |  | Yes |  | 1·17 | 0·9 | 1·5 | **0·264** |  |  |  |  |  |
|  | Before eating | No |  | 1 |  |  |  |  |  |  |  |  |
|  |  | Yes |  | 1·11 | 0·79 | 1·57 | **0·533** |  |  |  |  |  |
| Hand washing per week | | < 4 |  | 1 |  |  | **0·634** |  |  |  |  |  |
|  |  | < 8 |  | 0·91 | 0·7 | 1·2 | 0·552 |  |  |  |  |  |
|  |  | ≥ 8 |  | 0·83 | 0·6 | 1·2 | 0·351 |  |  |  |  |  |
| Use soap to wash your hand | | No |  | 1 |  |  |  |  |  |  |  |  |
|  |  | Yes |  | 1·04 | 0·8 | 1·4 | **0·821** |  |  |  |  |  |
| Deworming (month) | | < 6 |  | 1 |  |  | **0·696** |  |  |  |  |  |
|  |  | 6-12 |  | 0·84 | 0·6 | 1·3 | 0·423 |  |  |  |  |  |
|  |  | > 12 |  | 0·90 | 0·7 | 1·2 | 0·522 |  |  |  |  |  |

*p-value and odds ratio (OR) based on likelihood ratio test of univariate logistic regression, overall p-value of the models are indicated in bold letters. ** p-value and adjusted (a) OR based on likelihood ratio test of the multivariate regression model. The multivariate model was defined including exposure groups, sex, age, educational attainment, socioeconomic status, and number of people per household. In addition, all risk factors that had a p-value lower than 0.2 in the univariate analyses were included into the multivariate regression analysis (as indicated in the table). *** exposure groups: *com _exposed_*, slum dwellers at risk of flooding along the Nakivubo wetland; *com _comparison_*, slum dwellers without risk of flooding at least 2 km away from the Nakivubo wetland; *farmer*, urban farmers reusing wastewater within the Nakivubo wetland; *worker _ww_*, workers maintaining drainage channels and operating the Bugolobi Sewage Treatment Works; *worker _fs_*, workers managing fecal sludge (e.g., collection at households by means of vacuum trucks).

**S2D** Results of univariate and the multivariate logistic regression analysis for Schistosoma mansoni infection in a cross-sectional survey done in late 2013 in Kampala

| *Schistosoma mansoni* infection  N(total)=915 / N(cases)= 110 | | |  | Univariate logistic regression* | | | |  | Multivariate logistic regression** | | | |
| --- | --- | --- | --- | --- | --- | --- | --- | --- | --- | --- | --- | --- |
|  |  |  |  | OR | 95% CI | | p-value |  | aOR | 95% CI | | p-value |
| Exposure group*** | | *com _comparison_* |  | 1 |  |  | **<0·001** |  | 1 |  |  | **<0·001** |
|  |  | *com* *_exposed_* |  | 2·17 | 1·2 | 3·9 | 0·018 |  | 1·91 | 0·98 | 3·73 | 0·061 |
|  |  | *farmer* |  | 4·61 | 2·7 | 7·9 | <0·001 |  | 3·75 | 1·89 | 7·45 | <0·001 |
|  |  | *worker _fs_* |  | 0·99 | 0·3 | 3·0 | 0·982 |  | 0·62 | 0·19 | 1·99 | 0·422 |
|  |  | *worker* *_ww_* |  | 0·76 | 0·2 | 3·4 | 0·721 |  | 0·43 | 0·09 | 2·09 | 0·304 |
| Sex | | Male |  | 1 |  |  |  |  |  |  |  |  |
|  |  | Female |  | 0·49 | 0·3 | 0·7 | **<0·001** |  | 0·47 | 0·30 | 0·76 | <0·001 |
| Age | |  |  | 0·01 | 0·0 | 0·0 | **0·46** |  | 1·00 | 0·98 | 1·02 | 0·786 |
| Education | | Never went to school |  | 1 |  |  | **0·033** |  |  |  |  |  |
|  |  | Primary |  | 2·21 | 1·1 | 4·6 | 0·032 |  | 1·77 | 0·82 | 3·84 | 0·155 |
|  |  | Higher education |  | 1·45 | 0·7 | 3·1 | 0·332 |  | 1·93 | 0·85 | 4·40 | 0·120 |
| Socio-economic status | | Most poor |  | 1 |  |  | **0·341** |  |  |  |  |  |
|  |  | Poor |  | 0·83 | 0·5 | 1·3 | 0·431 |  | 1·12 | 0·66 | 1·89 | 0·671 |
|  |  | Less poor |  | 0·69 | 0·4 | 1·1 | 0·156 |  | 1·19 | 0·66 | 2·15 | 0·551 |
| Number of people per household | | Single |  | 1 |  |  | **0·352** |  |  |  |  |  |
|  |  | 2 to 4 |  | 1·38 | 0·7 | 2·8 | 0·371 |  | 1·43 | 0·68 | 3·01 | 0·343 |
|  |  | > 4 |  | 1·65 | 0·8 | 3·4 | 0·188 |  | 1·53 | 0·70 | 3·34 | 0·298 |
| Toilet facility | | Flush toilet |  | 1 |  |  | **0·684** |  |  |  |  |  |
|  |  | Pit latrine |  | 1·02 | 0·4 | 2·4 | 0·973 |  |  |  |  |  |
|  |  | No facility |  | 1·32 | 0·5 | 3·6 | 0·597 |  |  |  |  |  |
| Toilet sharing | | Private toilet |  | 1 |  |  | **0·223** |  |  |  |  |  |
|  |  | 2 and 3 households |  | 0·80 | 0·5 | 1·3 | 0·403 |  |  |  |  |  |
|  |  | ≥ 4 households |  | 1·20 | 0·7 | 2·0 | 0·468 |  |  |  |  |  |
| Flooding of living area | | No |  | 1 |  |  |  |  |  |  |  |  |
|  |  | Yes |  | 2·43 | 1·62 | 3·63 | **<0·001** |  | 1·21 | 0·72 | 2·03 | 0·471 |
| Source of drinking water | | Bottle, Tab, rain |  | 1 |  |  | **0·021** |  |  |  |  |  |
|  |  | Spring |  | 1·71 | 1·13 | 2·58 | 0·010 |  | 1·36 | 0·63 | 2·92 | 0·431 |
|  |  | Other |  | 0·73 | 0·3 | 1·9 | 0·510 |  | 0·56 | 0·18 | 1·78 | 0·332 |
| Source of bath water | | Tab, rain water |  | 1 |  |  | **0·023** |  |  |  |  |  |
|  |  | Spring |  | 1·68 | 1·1 | 2·5 | 0·024 |  | 1·13 | 0·52 | 2·46 | 0·754 |
|  |  | Unprotected |  | 0·85 | 0·4 | 1·9 | 0·682 |  | 0·82 | 0·32 | 2·13 | 0·681 |
| Bathing per week | | **<** 7 |  | 1 |  |  | **0·486** |  |  |  |  |  |
|  |  | ≥ 7 to < 14 |  | 0·61 | 0·3 | 1·3 | 0·214 |  |  |  |  |  |
|  |  | > 14 |  | 0·66 | 0·3 | 1·4 | 0·298 |  |  |  |  |  |
| Hand washing | After defecation | No |  | 1 |  |  |  |  |  |  |  |  |
|  |  | Yes |  | 0·99 | 0·64 | 1·53 | **0·951** |  |  |  |  |  |
|  | After work | No |  | 1 |  |  |  |  |  |  |  |  |
|  |  | Yes |  | 1·10 | 0·7 | 1·6 | **0·641** |  |  |  |  |  |
|  | Before eating | No |  | 1 |  |  |  |  |  |  |  |  |
|  |  | Yes |  | 1·05 | 0·63 | 1·75 | **0·863** |  |  |  |  |  |
| Hand washing per week | | < 4 |  | 1 |  |  | **0·557** |  |  |  |  |  |
|  |  | < 8 |  | 0·88 | 0·6 | 1·4 | 0·601 |  |  |  |  |  |
|  |  | ≥ 8 |  | 0·73 | 0·4 | 1·3 | 0·282 |  |  |  |  |  |
| Use soap to wash your hand | | No |  | 1 |  |  |  |  |  |  |  |  |
|  |  | Yes |  | 0·69 | 0·4 | 1·2 | **0·165** |  |  |  |  |  |
| Deworming | | < 6 month |  | 1 |  |  | **0·575** |  |  |  |  |  |
|  |  | 6 to < 12 month |  | 1·28 | 0·7 | 2·3 | 0·412 |  |  |  |  |  |
|  |  | > 12 month |  | 0·95 | 0·6 | 1·5 | 0·840 |  |  |  |  |  |

*p-value and odds ratio (OR) based on likelihood ratio test of univariate logistic regression, overall p-value of the models are indicated in bold letters. ** p-value and adjusted (a) OR based on likelihood ratio test of the multivariate regression model. The multivariate model was defined including exposure groups, sex, age, educational attainment, socioeconomic status, and number of people per household. In addition, all risk factors that had a p-value lower than 0.2 in the univariate analyses were included into the multivariate regression analysis (as indicated in the table). *** exposure groups: *com _exposed_*, slum dwellers at risk of flooding along the Nakivubo wetland; *com _comparison_*, slum dwellers without risk of flooding at least 2 km away from the Nakivubo wetland; *farmer*, urban farmers reusing wastewater within the Nakivubo wetland; *worker _ww_*, workers maintaining drainage channels and operating the Bugolobi Sewage Treatment Works; *worker _fs_*, workers managing fecal sludge (e.g., collection at households by means of vacuum trucks).

**S2E** Results of univariate and the multivariate logistic regression analysis for hookworm infection in a cross-sectional survey done in late 2013 in Kampala

| Hookworm infection  N(total)=915 / N(cases)= 115 | | |  | Univariate logistic regression* | | | |  | Multivariate logistic regression** | | | |
| --- | --- | --- | --- | --- | --- | --- | --- | --- | --- | --- | --- | --- |
|  |  |  |  | OR | 95% CI | | p-value |  | aOR | 95% CI | | p-value |
| Exposure group*** | | *com _comparison_* |  | 1 |  |  | **<0·001** |  | 1 |  |  | **<0·001** |
|  |  | *com* *_exposed_* |  | 0·44 | 0·2 | 1·0 | 0·042 |  | 0·44 | 0·19 | 1·00 | 0·052 |
|  |  | *farmer* |  | 4·16 | 2·6 | 6·7 | <0·001 |  | 3·49 | 1·79 | 6·79 | <0·001 |
|  |  | *worker _fs_* |  | 0·51 | 0·1 | 1·7 | 0·281 |  | 0·39 | 0·10 | 1·44 | 0·166 |
|  |  | *worker* *_ww_* |  | 2·10 | 0·9 | 5·2 | 0·105 |  | 2·05 | 0·72 | 5·85 | 0·181 |
| Sex | | Male |  |  |  |  |  |  |  |  |  |  |
|  |  | Female |  | 0·52 | 0·4 | 0·8 | **<0·001** |  | 0·67 | 0·42 | 1·08 | 0·101 |
| Age | |  |  | 0·02 | 0·0 | 0·0 | **0·021** |  | 1·00 | 0·98 | 1·02 | 0·817 |
| Education | | Never went to school |  |  |  |  | **<0·001** |  |  |  |  |  |
|  |  | Primary |  | 1·17 | 0·7 | 2·1 | 0·593 |  | 1·07 | 0·57 | 2·02 | 0·833 |
|  |  | Higher education |  | 0·49 | 0·3 | 0·9 | 0·029 |  | 0·57 | 0·28 | 1·17 | 0·123 |
| Socio-economic status | | Most poor |  |  |  |  | **0·291** |  |  |  |  |  |
|  |  | Poor |  | 0·74 | 0·5 | 1·2 | 0·212 |  | 0·94 | 0·54 | 1·62 | 0·819 |
|  |  | Less poor |  | 0·71 | 0·4 | 1·1 | 0·163 |  | 1·20 | 0·65 | 2·20 | 0·561 |
| Number of people per household | | Single |  |  |  |  | **0·943** |  |  |  |  |  |
|  |  | 2 to 4 |  | 1·00 | 0·5 | 1·9 | 0·991 |  |  |  |  |  |
|  |  | > 4 |  | 1·07 | 0·6 | 2·1 | 0·830 |  |  |  |  |  |
| Toilet facility | | Flush toilet |  |  |  |  | **0·055** |  |  |  |  |  |
|  |  | Pit latrine |  | 0·83 | 0·4 | 1·9 | 0·668 |  |  |  |  |  |
|  |  | No facility |  | 1·67 | 0·7 | 4·2 | 0·281 |  |  |  |  |  |
| Toilet sharing | | Private toilet |  |  |  |  | **0·091** |  |  |  |  |  |
|  |  | 2 and 3 households |  | 0·60 | 0·4 | 1·0 | 0·052 |  | 0·79 | 0·45 | 1·41 | 0·438 |
|  |  | ≥ 4 households |  | 0·91 | 0·6 | 1·5 | 0·710 |  | 1·18 | 0·63 | 2·20 | 0·612 |
| Flooding of living area | | No |  |  |  |  |  |  |  |  |  |  |
|  |  | Yes |  | 1·65 | 1·11 | 2·46 | **0·010** |  | 0·73 | 0·40 | 1·35 | 0·315 |
| Source of drinking water | | Bottle, Tab, rain |  |  |  |  | **0·471** |  |  |  |  |  |
|  |  | Spring |  | 1·25 | 0·83 | 1·90 | 0·292 |  |  |  |  |  |
|  |  | Other |  | 1·38 | 0·7 | 2·8 | 0·381 |  |  |  |  |  |
| Source of bath water | | Tab, rain water |  |  |  |  | **<0·016** |  |  |  |  |  |
|  |  | Spring |  | 1·31 | 0·9 | 2·0 | 0·225 |  | 1·09 | 0·67 | 1·79 | 0·722 |
|  |  | Unprotected |  | 2·57 | 1·5 | 4·5 | <0·0018 |  | 1·98 | 1·06 | 3·68 | 0·032 |
| Bathing per week | | **<** 7 |  |  |  |  | **<0·012** |  |  |  |  |  |
|  |  | ≥ 7 to < 14 |  | 0·58 | 0·3 | 1·2 | 0·131 |  | 0·78 | 0·36 | 1·70 | 0·544 |
|  |  | > 14 |  | 0·34 | 0·2 | 0·7 | <0·001 |  | 0·62 | 0·27 | 1·41 | 0·251 |
| Hand washing | After defecation | No |  |  |  |  |  |  |  |  |  |  |
|  |  | Yes |  | 0·47 | 0·32 | 0·71 | <0·001 |  | 0·75 | 0·47 | 1·20 | 0·233 |
|  | After work | No |  |  |  |  |  |  |  |  |  |  |
|  |  | Yes |  | 1·36 | 0·9 | 2·0 | **0·133** |  | 0·83 | 0·52 | 1·32 | 0·442 |
|  | Before eating | No |  |  |  |  |  |  |  |  |  |  |
|  |  | Yes |  | 1·11 | 0·67 | 1·86 | **0·682** |  |  |  |  |  |
| Hand washing per week | | < 4 |  |  |  |  | **0·193** |  |  |  |  |  |
|  |  | < 8 |  | 1·13 | 0·7 | 1·8 | 0·612 |  |  |  |  |  |
|  |  | ≥ 8 |  | 0·70 | 0·4 | 1·3 | 0·244 |  |  |  |  |  |
| Use soap to wash your hand | | No |  |  |  |  |  |  |  |  |  |  |
|  |  | Yes |  | 1·12 | 0·7 | 1·8 | **0·642** |  |  |  |  |  |
| Deworming | | < 6 month |  |  |  |  | **0·801** |  |  |  |  |  |
|  |  | 6 to < 12 month |  | 1·18 | 0·6 | 2·2 | 0·591 |  |  |  |  |  |
|  |  | > 12 month |  | 1·15 | 0·7 | 1·8 | 0·560 |  |  |  |  |  |

*p-value and odds ratio (OR) based on likelihood ratio test of univariate logistic regression, overall p-value of the models are indicated in bold letters. ** p-value and adjusted (a) OR based on likelihood ratio test of the multivariate regression model. The multivariate model was defined including exposure groups, sex, age, educational attainment, socioeconomic status, and number of people per household. In addition, all risk factors that had a p-value lower than 0.2 in the univariate analyses were included into the multivariate regression analysis (as indicated in the table). *** exposure groups: *com _exposed_*, slum dwellers at risk of flooding along the Nakivubo wetland; *com _comparison_*, slum dwellers without risk of flooding at least 2 km away from the Nakivubo wetland; *farmer*, urban farmers reusing wastewater within the Nakivubo wetland; *worker _ww_*, workers maintaining drainage channels and operating the Bugolobi Sewage Treatment Works; *worker _fs_*, workers managing fecal sludge (e.g., collection at households by means of vacuum trucks).

**S2F** Results of univariate and the multivariate logistic regression analysis for Trichuris trichiura infections in a cross-sectional survey done in late 2013 in Kampala

| ***Trichuris trichiura* infection**  **N(total)=915 / N(cases)=79** | | |  | **Univariate logistic regression*** | | | |  | **Multivariate logistic regression**** | | | |
| --- | --- | --- | --- | --- | --- | --- | --- | --- | --- | --- | --- | --- |
|  |  |  |  | OR | 95% CI | | p-value |  | aOR | 95% CI | | p-value |
| Exposure group*** | | *com _comparison_* |  | 1 |  |  | **<0·001** |  | 1 |  |  | **<0·001** |
|  |  | *com* *_exposed_* |  | 1·96 | 0·67 | 5·73 | 0·221 |  | 1·64 | 0·52 | 5·14 | 0·403 |
|  |  | *farmer* |  | 19·1 | 8·13 | 45·10 | <0·001 |  | 12·9 | 4·50 | 37·5 | <0·001 |
|  |  | *worker _fs_* |  | - |  |  |  |  | - |  |  |  |
|  |  | *worker* *_ww_* |  | 1·29 | 0·15 | 10·98 | 0·822 |  | 0·41 | 0·04 | 4·13 | 0·453 |
| Sex | | Male |  | 1 |  |  |  |  |  |  |  |  |
|  |  | Female |  | 0·44 | 0·27 | 0·70 | **<0·001** |  | 0·54 | 0·29 | 0·98 | 0·044 |
| Age | |  |  | 0·03 | 0·01 | 0·05 | **<0·001** |  | 1·01 | 0·99 | 1·04 | 0·317 |
| Education | | Never went to school |  | 1 |  |  | **0·031** |  |  |  |  |  |
|  |  | Primary |  | 1·00 | 0·52 | 1·95 | 0·991 |  | 0·86 | 0·39 | 1·90 | 0·715 |
|  |  | Higher education |  | 0·53 | 0·27 | 1·07 | 0·088 |  | 1·07 | 0·43 | 2·66 | 0·883 |
| Socio-economic status | | Most poor |  | 1 |  |  | **0·022** |  |  |  |  |  |
|  |  | Poor |  | 0·51 | 0·29 | 0·90 | 0·021 |  | 0·86 | 0·44 | 1·67 | 0·654 |
|  |  | Less poor |  | 0·52 | 0·30 | 0·92 | 0·020 |  | 1·80 | 0·87 | 3·76 | 0·125 |
| Number of people per household | | Single |  | 1 |  |  | **0·045** |  |  |  |  |  |
|  |  | 2 to 4 |  | 1·84 | 0·70 | 4·79 | 0·216 |  | 1·95 | 0·67 | 5·63 | 0·227 |
|  |  | > 4 |  | 2·82 | 1·08 | 7·38 | 0·033 |  | 2·44 | 0·82 | 7·26 | 0·119 |
| Toilet facility | | Flush toilet |  | 1 |  |  | **<0·001** |  |  |  |  |  |
|  |  | Pit latrine |  | 2·06 | 0·49 | 8·69 | 0·322 |  | 4·19 | 0·89 | 19·7 | 0·073 |
|  |  | No facility |  | 5·72 | 1·28 | 25·61 | 0·022 |  | 5·60 | 1·04 | 30·0 | 0·042 |
| Toilet sharing | | Private toilet |  | 1 |  |  | **0·09** |  |  |  |  |  |
|  |  | 2 and 3 households |  | 0·75 | 0·40 | 1·41 | 0·375 |  |  |  |  |  |
|  |  | ≥ 4 households |  | 1·38 | 0·78 | 2·45 | 0·274 |  |  |  |  |  |
| Flooding of living area | | No |  | 1 |  |  |  |  |  |  |  |  |
|  |  | Yes |  | 4·84 | 2·97 | 7·89 | **<0·001** |  | 1·29 | 0·63 | 2·66 | 0·494 |
| Source of drinking water | | Bottle, Tab, rain |  | 1 |  |  | **0·604** |  |  |  |  |  |
|  |  | Spring |  | 0·97 | 0·59 | 1·61 | 0·918 |  |  |  |  |  |
|  |  | Other |  | 1·50 | 0·67 | 3·33 | 0·322 |  |  |  |  |  |
| Source of bath water | | Tab, rain water |  | 1 |  |  | **0·500** |  |  |  |  |  |
|  |  | Spring |  | 0·93 | 0·56 | 1·55 | 0·782 |  |  |  |  |  |
|  |  | Unprotected |  | 1·46 | 0·72 | 2·96 | 0·293 |  |  |  |  |  |
| Bathing per week | | **<** 7 |  | 1 |  |  | **0·275** |  |  |  |  |  |
|  |  | ≥ 7 to < 14 |  | 0·67 | 0·28 | 1·57 | 0·362 |  |  |  |  |  |
|  |  | > 14 |  | 0·51 | 0·21 | 1·22 | 0·131 |  |  |  |  |  |
| Hand washing | After defecation | No |  | 1 |  |  |  |  |  |  |  |  |
|  |  | Yes |  | 0·38 | 0·24 | 0·60 | **<0·001** |  | 0·43 | 0·23 | 0·77 | 0.014 |
|  | After work | No |  | 1 |  |  |  |  |  |  |  |  |
|  |  | Yes |  | 1·48 | 0·92 | 2·37 | **0·111** |  | 0·59 | 0·32 | 1·09 | 0.103 |
|  | Before eating | No |  | 1 |  |  |  |  |  |  |  |  |
|  |  | Yes |  | 2·17 | 1·02 | 4·59 | **0·042** |  | 1·50 | 0·62 | 3·61 | 0.377 |
| Hand washing per week | | < 4 |  | 1 |  |  | **0·081** |  |  |  |  |  |
|  |  | < 8 |  | 0·78 | 0·47 | 1·31 | 0·354 |  | 1·06 | 0·58 | 1·92 | 0·863 |
|  |  | ≥ 8 |  | 0·45 | 0·22 | 0·94 | 0·038 |  | 0·83 | 0·35 | 1·95 | 0·666 |
| Use soap to wash your hand | | No |  | 1 |  |  |  |  |  |  |  |  |
|  |  | Yes |  | 0·59 | 0·31 | 1·11 | **0·103** |  | 0·73 | 0·35 | 1·51 | 0·401 |
| Deworming | | < 6 month |  | 1 |  |  | **0·747** |  |  |  |  |  |
|  |  | 6 to < 12 month |  | 0·77 | 0·36 | 1·66 | 0·516 |  |  |  |  |  |
|  |  | > 12 month |  | 1·01 | 0·60 | 1·70 | 0·973 |  |  |  |  |  |

*p-value and odds ratio (OR) based on likelihood ratio test of univariate logistic regression, overall p-value of the models are indicated in bold letters. ** p-value and adjusted (a) OR based on likelihood ratio test of the multivariate regression model. The multivariate model was defined including exposure groups, sex, age, educational attainment, socioeconomic status, and number of people per household. In addition, all risk factors that had a p-value lower than 0.2 in the univariate analyses were included into the multivariate regression analysis (as indicated in the table). *** exposure groups: *com _exposed_*, slum dwellers at risk of flooding along the Nakivubo wetland; *com _comparison_*, slum dwellers without risk of flooding at least 2 km away from the Nakivubo wetland; *farmer*, urban farmers reusing wastewater within the Nakivubo wetland; *worker _ww_*, workers maintaining drainage channels and operating the Bugolobi Sewage Treatment Works; *worker _fs_*, workers managing fecal sludge (e.g., collection at households by means of vacuum trucks).

**S2G** Results of univariate and the multivariate logistic regression analysis for 14-day diarrhea prevalence in a cross-sectional survey done in late 2013 in Kampala

| **14-day diarrhoea prevalence**  N(total)=915 / N(cases)= 222 | | |  | **Univariate logistic regression*** | | | |  | **Multivariate logistic regression**** | | | |
| --- | --- | --- | --- | --- | --- | --- | --- | --- | --- | --- | --- | --- |
|  |  |  |  | OR | 95% CI | | p-value |  | aOR | 95% CI | | p-value |
| Exposure group*** | | *com _comparison_* |  | 1 |  |  | **0·331** |  | 1 |  |  | **0·273** |
|  |  | *com* *_exposed_* |  | 1·26 | 0·8 | 1·9 | 0·252 |  | 1·10 | 0·72 | 1·67 | 0·665 |
|  |  | *farmer* |  | 1·26 | 0·9 | 1·9 | 0·241 |  | 1·01 | 0·65 | 1·58 | 0·962 |
|  |  | *worker _fs_* |  | 1·82 | 1·0 | 3·2 | 0·042 |  | 1·67 | 0·86 | 3·25 | 0·132 |
|  |  | *worker* *_ww_* |  | 1·13 | 0·5 | 2·4 | 0·751 |  | 1·25 | 0·53 | 2·92 | 0·616 |
| Sex | | Male |  | 1 |  |  |  |  |  |  |  |  |
|  |  | Female |  | 0·83 | 0·6 | 1·1 | **0·232** |  | 0·87 | 0·61 | 1·24 | 0·445 |
| Age | |  |  | 0·00 | 0·0 | 0·0 | **0·531** |  | 1·00 | 0·99 | 1·01 | 0·954 |
| Education | | Never went to school |  | 1 |  |  | **0·194** |  |  |  |  |  |
|  |  | Primary |  | 0·94 | 0·6 | 1·5 | 0·815 |  | 0·92 | 0·56 | 1·49 | 0·725 |
|  |  | Higher education |  | 0·72 | 0·5 | 1·1 | 0·178 |  | 0·71 | 0·43 | 1·19 | 0·204 |
| Socio-economic status | | Most poor |  | 1 |  |  | **0·235** |  |  |  |  |  |
|  |  | Poor |  | 1·32 | 0·9 | 1·9 | 0·143 |  | 1·49 | 1·01 | 2·20 | 0·045 |
|  |  | Less poor |  | 1·01 | 0·7 | 1·5 | 0·956 |  | 1·12 | 0·72 | 1·74 | 0·616 |
| Number of people per household | | Single |  | 1 |  |  | **0·906** |  |  |  |  |  |
|  |  | 2 to 4 |  | 1·08 | 0·7 | 1·8 | 0·754 |  |  |  |  |  |
|  |  | > 4 |  | 1·12 | 0·7 | 1·9 | 0·678 |  |  |  |  |  |
| Toilet facility | | Flush toilet |  | 1 |  |  | **0·242** |  |  |  |  |  |
|  |  | Pit latrine |  | 1·31 | 0·6 | 2·7 | 0·461 |  | 1·29 | 0·61 | 2·74 | 0·506 |
|  |  | No facility |  | 1·83 | 0·8 | 4·1 | 0·147 |  | 1·72 | 0·71 | 4·16 | 0·236 |
| Toilet sharing | | Private toilet |  | 1 |  |  | **0·382** |  |  |  |  |  |
|  |  | 2 and 3 households |  | 1·03 | 0·7 | 1·5 | 0·903 |  |  |  |  |  |
|  |  | ≥ 4 households |  | 1·27 | 0·9 | 1·9 | 0·234 |  |  |  |  |  |
| Flooding of living area | | No |  |  |  |  |  |  |  |  |  |  |
|  |  | Yes |  | 1·05 | 0·76 | 1·45 | **0·795** |  |  |  |  |  |
| Source of drinking water | | Bottle, Tab, rain |  | 1 |  |  | **0·114** |  |  |  |  |  |
|  |  | Spring |  | 1·39 | 1·01 | 1·91 | 0·053 |  | 1·33 | 0·77 | 2·28 | 0·312 |
|  |  | Other |  | 1·35 | 0·8 | 2·4 | 0·313 |  | 1·19 | 0·58 | 2·44 | 0·643 |
| Source of bath water | | Tab, rain water |  | 1 |  |  | **0·175** |  |  |  |  |  |
|  |  | Spring |  | 1·33 | 1·0 | 1·8 | 0·088 |  | 1·06 | 0·61 | 1·83 | 0·842 |
|  |  | Unprotected |  | 1·34 | 0·8 | 2·2 | 0·265 |  | 1·24 | 0·66 | 2·34 | 0·511 |
| Bathing per week | | **<** 7 |  | 1 |  |  | **0·454** |  |  |  |  |  |
|  |  | ≥ 7 to < 14 |  | 1·10 | 0·5 | 2·2 | 0·795 |  |  |  |  |  |
|  |  | > 14 |  | 1·32 | 0·7 | 2·6 | 0·448 |  |  |  |  |  |
| Hand washing | After defecation | No |  | 1 |  |  |  |  |  |  |  |  |
|  |  | Yes |  | 0·84 | 0·61 | 1·17 | **0·319** |  |  |  |  |  |
|  | After work | No |  | 1 |  |  |  |  |  |  |  |  |
|  |  | Yes |  | 1·12 | 0·8 | 1·5 | **0·485** |  |  |  |  |  |
|  | Before eating | No |  | 1 |  |  |  |  |  |  |  |  |
|  |  | Yes |  | 0·92 | 0·63 | 1·34 | **0·654** |  |  |  |  |  |
| Hand washing per week | | < 4 |  | 1 |  |  | **0·995** |  |  |  |  |  |
|  |  | < 8 |  | 1·02 | 0·7 | 1·5 | 0·925 |  | 0·85 | 0·53 | 1·37 | 0·502 |
|  |  | ≥ 8 |  | 1·00 | 0·7 | 1·5 | 0·996 |  | 0·58 | 0·40 | 0·83 | 0·001 |
| Use soap to wash your hand | | No |  | 1 |  |  |  |  |  |  |  |  |
|  |  | Yes |  | 0·80 | 0·6 | 1·2 | **0·254** |  |  |  |  |  |
| Deworming (month) | | < 6 |  | 1 |  |  | **0·014** |  |  |  |  |  |
|  |  | 6-12 |  | 0·84 | 0·5 | 1·3 | 0·473 |  |  |  |  |  |
|  |  | > 12 |  | 0·60 | 0·4 | 0·8 | 0·001 |  |  |  |  |  |

*p-value and odds ratio (OR) based on likelihood ratio test of univariate logistic regression, overall p-value of the models are indicated in bold letters. ** p-value and adjusted (a) OR based on likelihood ratio test of the multivariate regression model. The multivariate model was defined including exposure groups, sex, age, educational attainment, socioeconomic status, and number of people per household. In addition, all risk factors that had a p-value lower than 0.2 in the univariate analyses were included into the multivariate regression analysis (as indicated in the table). *** exposure groups: *com _exposed_*, slum dwellers at risk of flooding along the Nakivubo wetland; *com _comparison_*, slum dwellers without risk of flooding at least 2 km away from the Nakivubo wetland; *farmer*, urban farmers reusing wastewater within the Nakivubo wetland; *worker _ww_*, workers maintaining drainage channels and operating the Bugolobi Sewage Treatment Works; *worker _fs_*, workers managing fecal sludge (e.g., collection at households by means of vacuum trucks).

**S2H** Results of univariate and the multivariate logistic regression analysis for skin problems over the past two weeks in a cross-sectional survey done in late 2013 in Kampala

| **Skin problems over the past two weeks** N(total)=915 / N(cases)= 279 | | |  | **Univariate logistic regression*** | | | |  | **Multivariate logistic regression**** | | | |
| --- | --- | --- | --- | --- | --- | --- | --- | --- | --- | --- | --- | --- |
|  |  |  |  | OR | 95% CI | | p-value |  | aOR | 95% CI | | p-value |
| Exposure group*** | | *com _comparison_* |  | 1 |  |  | **<0·001** |  | 1 |  |  | **<0·001** |
|  |  | *com* *_exposed_* |  | 0·96 | 0·7 | 1·4 | 0·85 |  | 0·90 | 0·58 | 1·40 | 0·643 |
|  |  | *farmer* |  | 1·23 | 0·9 | 1·8 | 0·26 |  | 1·50 | 0·91 | 2·47 | 0·114 |
|  |  | *worker _fs_* |  | 1·58 | 0·9 | 2·7 | 0·10 |  | 1·11 | 0·57 | 2·15 | 0·777 |
|  |  | *worker* *_ww_* |  | 1·08 | 0·5 | 2·2 | 0·84 |  | 0·91 | 0·40 | 2·06 | 0·825 |
| Sex | | Male |  |  |  |  |  |  |  |  |  |  |
|  |  | Female |  | 0·63 | 0·5 | 0·8 | **<0·001** |  | 0·60 | 0·42 | 0·86 | 0·011 |
| Age | |  |  | 0·00 | 0·0 | 0·0 | **0·48** |  | 1·00 | 0·99 | 1·01 | 0·901 |
| Education | | Never went to school |  | 1 |  |  | **0·15** |  |  |  |  |  |
|  |  | Primary |  | 0·85 | 0·5 | 1·3 | 0·45 |  | 0·75 | 0·47 | 1·20 | 0·242 |
|  |  | Higher education |  | 0·68 | 0·4 | 1·1 | 0·08 |  | 0·60 | 0·37 | 0·99 | 0·051 |
| Socio-economic status | | Most poor |  | 1 |  |  | **<0·001** |  |  |  |  |  |
|  |  | Poor |  | 0·95 | 0·7 | 1·4 | 0·77 |  | 1·05 | 0·71 | 1·56 | 0·802 |
|  |  | Less poor |  | 1·67 | 1·2 | 2·4 | <0·001 |  | 2·17 | 1·42 | 3·33 | <0·001 |
| Number of people per household | | Single |  | 1 |  |  | **0·25** |  |  |  |  |  |
|  |  | 2 to 4 |  | 0·94 | 0·6 | 1·5 | 0·79 |  |  |  |  |  |
|  |  | > 4 |  | 0·74 | 0·5 | 1·2 | 0·21 |  |  |  |  |  |
| Toilet facility | | Flush toilet |  | 1 |  |  | **0·13** |  |  |  |  |  |
|  |  | Pit latrine |  | 1·03 | 0·6 | 1·9 | 0·93 |  |  |  |  |  |
|  |  | No facility |  | 1·59 | 0·8 | 3·3 | 0·21 |  |  |  |  |  |
| Toilet sharing | | Private toilet |  | 1 |  |  | **0·06** |  |  |  |  |  |
|  |  | 2 and 3 households |  | 0·78 | 0·5 | 1·1 | 0·18 |  | 1·02 | 0·68 | 1·52 | 0·932 |
|  |  | ≥ 4 households |  | 1·16 | 0·8 | 1·6 | 0·42 |  | 1·47 | 0·95 | 2·27 | 0·083 |
| Flooding of living area | | No |  | 1 |  |  |  |  |  |  |  |  |
|  |  | Yes |  | 0·80 | 0·58 | 1·08 | **0·15** |  | 0·68 | 0·45 | 1·04 | 0·084 |
| Source of drinking water | | Bottle, Tab, rain |  | 1 |  |  | **0·14** |  |  |  |  |  |
|  |  | Spring |  | 1·31 | 0·97 | 1·77 | 0·07 |  | 1·08 | 0·64 | 1·81 | 0·783 |
|  |  | Other |  | 1·10 | 0·6 | 1·9 | 0·74 |  | 1·12 | 0·56 | 2·25 | 0·756 |
| Source of bath water | | Tab, rain water |  | 1 |  |  | **0·07** |  |  |  |  |  |
|  |  | Spring |  | 1·40 | 1·0 | 1·9 | 0·03 |  | 1·40 | 0·83 | 2·37 | 0·202 |
|  |  | Unprotected |  | 1·02 | 0·6 | 1·7 | 0·94 |  | 0·87 | 0·46 | 1·64 | 0·661 |
| Bathing per week | | **<** 7 |  | 1 |  |  | **0·08** |  |  |  |  |  |
|  |  | ≥ 7 to < 14 |  | 1·99 | 1·0 | 4·1 | 0·06 |  | 2·12 | 0·98 | 4·58 | 0·067 |
|  |  | > 14 |  | 1·82 | 0·9 | 3·7 | 0·10 |  | 2·21 | 1·00 | 4·90 | 0·054 |
| Hand washing | After defecation | No |  | 1 |  |  |  |  |  |  |  |  |
|  |  | Yes |  | 0·74 | 0·54 | 1·00 | **0·05** |  | 0·73 | 0·52 | 1·04 | 0.092 |
|  | After work | No |  | 1 |  |  |  |  |  |  |  |  |
|  |  | Yes |  | 0·60 | 0·5 | 0·8 | **<0·001** |  | 0·52 | 0·37 | 0·72 | <0.001 |
|  | Before eating | No |  | 1 |  |  |  |  |  |  |  |  |
|  |  | Yes |  | 0·71 | 0·50 | 1·00 | **0·05** |  |  |  |  |  |
| Hand washing per week | | < 4 |  | 1 |  |  | **0·04** |  |  |  |  |  |
|  |  | < 8 |  | 0·74 | 0·5 | 1·0 | 0·07 |  | 0·77 | 0·54 | 1·10 | 0·156 |
|  |  | ≥ 8 |  | 0·60 | 0·4 | 0·9 | 0·02 |  | 0·64 | 0·41 | 1·02 | 0·061 |
| Use soap to wash your hand | | No |  | 1 |  |  |  |  |  |  |  |  |
|  |  | Yes |  | 0·91 | 0·6 | 1·3 | **0·59** |  |  |  |  |  |

*p-value and odds ratio (OR) based on likelihood ratio test of univariate logistic regression, overall p-value of the models are indicated in bold letters. ** p-value and adjusted (a) OR based on likelihood ratio test of the multivariate regression model. The multivariate model was defined including exposure groups, sex, age, educational attainment, socioeconomic status, and number of people per household. In addition, all risk factors that had a p-value lower than 0.2 in the univariate analyses were included into the multivariate regression analysis (as indicated in the table). *** exposure groups: *com _exposed_*, slum dwellers at risk of flooding along the Nakivubo wetland; *com _comparison_*, slum dwellers without risk of flooding at least 2 km away from the Nakivubo wetland; *farmer*, urban farmers reusing wastewater within the Nakivubo wetland; *worker _ww_*, workers maintaining drainage channels and operating the Bugolobi Sewage Treatment Works; *worker _fs_*, workers managing fecal sludge (e.g., collection at households by means of vacuum trucks).

**S2I** Results of univariate and the multivariate logistic regression analysis for eye problems over the past two weeks prevalence in a cross-sectional survey done in late 2013 in Kampala

| **Eye problems over the past two weeks** N(total)=915 / N(cases)= 259 | | |  | **Univariate logistic regression*** | | | |  | **Multivariate logistic regression**** | | | |
| --- | --- | --- | --- | --- | --- | --- | --- | --- | --- | --- | --- | --- |
|  |  |  |  | OR | 95% CI | | p-value |  | aOR | 95% CI | | p-value |
| Exposure group*** | | *com _comparison_* |  | 1 |  |  | **<0·001** |  | 1 |  |  | **<0·001** |
|  |  | *com* *_exposed_* |  | 1·50 | 1·0 | 2·2 | 0·051 |  | 1·21 | 0·76 | 1·93 | 0·413 |
|  |  | *farmer* |  | 2·41 | 1·7 | 3·5 | <0·001 |  | 1·87 | 1·11 | 3·13 | 0·027 |
|  |  | *worker _fs_* |  | 1·93 | 1·1 | 3·4 | 0·033 |  | 2·32 | 1·16 | 4·64 | 0·024 |
|  |  | *worker* *_ww_* |  | 1·90 | 1·0 | 3·8 | 0·071 |  | 1·64 | 0·70 | 3·81 | 0·252 |
| Sex | | Male |  | 1 |  |  |  |  |  |  |  |  |
|  |  | Female |  | 0·87 | 0·6 | 1·2 | **0·332** |  | 1·12 | 0·78 | 1·62 | 0·543 |
| Age | |  |  | 0·04 | 0·0 | 0·1 | **<0·001** |  | 1·04 | 1·02 | 1·05 | 0·002 |
| Education | | Never went to school |  | 1 |  |  | **0·011** |  |  |  |  |  |
|  |  | Primary |  | 1·04 | 0·7 | 1·6 | 0·851 |  | 1·44 | 0·88 | 2·36 | 0·152 |
|  |  | Higher education |  | 0·67 | 0·4 | 1·0 | 0·088 |  | 1·13 | 0·67 | 1·91 | 0·653 |
| Socio-economic status | | Most poor |  | 1 |  |  | **0·332** |  |  |  |  |  |
|  |  | Poor |  | 0·78 | 0·5 | 1·1 | 0·172 |  | 0·90 | 0·61 | 1·33 | 0·593 |
|  |  | Less poor |  | 0·82 | 0·6 | 1·2 | 0·258 |  | 1·04 | 0·67 | 1·61 | 0·878 |
| Number of people per household | | Single |  | 1 |  |  | **<0·001** |  |  |  |  |  |
|  |  | 2 to 4 |  | 1·18 | 0·7 | 1·9 | 0·511 |  | 1·16 | 0·69 | 1·97 | 0·573 |
|  |  | > 4 |  | 2·00 | 1·2 | 3·3 | 0·017 |  | 1·69 | 0·97 | 2·95 | 0·072 |
| Toilet facility | | Flush toilet |  | 1 |  |  | **0·033** |  |  |  |  |  |
|  |  | Pit latrine |  | 1·25 | 0·6 | 2·4 | 0·522 |  | 1·58 | 0·76 | 3·27 | 0·225 |
|  |  | No facility |  | 2·15 | 1·0 | 4·6 | 0·056 |  | 1·52 | 0·63 | 3·66 | 0·353 |
| Toilet sharing | | Private toilet |  | 1 |  |  | **0·171** |  |  |  |  |  |
|  |  | 2 and 3 households |  | 0·96 | 0·7 | 1·4 | 0·831 |  | 1·10 | 0·72 | 1·67 | 0·664 |
|  |  | ≥ 4 households |  | 1·30 | 0·9 | 1·9 | 0·171 |  | 1·32 | 0·83 | 2·11 | 0·243 |
| Flooding of living area | | No |  | 1 |  |  |  |  |  |  |  |  |
|  |  | Yes |  | 1·70 | 1·25 | 2·29 | **<0·001** |  | 1·27 | 0·83 | 1·95 | 0·268 |
| Source of drinking water | | Bottle, Tab, rain |  | 1 |  |  | **0·187** |  |  |  |  |  |
|  |  | Spring |  | 1·33 | 0·98 | 1·81 | 0·073 |  | 1·21 | 0·71 | 2·08 | 0·487 |
|  |  | Other |  | 1·15 | 0·7 | 2·0 | 0·646 |  | 0·83 | 0·40 | 1·73 | 0·626 |
| Source of bath water | | Tab, rain water |  | 1 |  |  | **0·323** |  |  |  |  |  |
|  |  | Spring |  | 1·23 | 0·9 | 1·7 | 0·194 |  | 0·87 | 0·50 | 1·51 | 0·621 |
|  |  | Unprotected |  | 1·29 | 0·8 | 2·1 | 0·308 |  | 1·24 | 0·66 | 2·35 | 0·502 |
| Bathing per week | | **<** 7 |  | 1 |  |  | **0·244** |  |  |  |  |  |
|  |  | ≥ 7 to < 14 |  | 1·06 | 0·5 | 2·1 | 0·873 |  |  |  |  |  |
|  |  | > 14 |  | 1·34 | 0·7 | 2·6 | 0·385 |  |  |  |  |  |
| Hand washing | After defecation | No |  | 1 |  |  |  |  |  |  |  |  |
|  |  | Yes |  | 0·92 | 0·67 | 1·26 | **0·601** |  |  |  |  |  |
|  | After work | No |  | 1 |  |  |  |  |  |  |  |  |
|  |  | Yes |  | 0·77 | 0·6 | 1·0 | **0·081** |  | 0·54 | 0·39 | 0·76 | <0.001 |
|  | Before eating | No |  | 1 |  |  |  |  |  |  |  |  |
|  |  | Yes |  | 1·49 | 1·01 | 2·21 | **0·056** |  | 1·3 | 0·84 | 2·00 | 0.242 |
| Hand washing per week | | < 4 |  | 1 |  |  | **0·544** |  |  |  |  |  |
|  |  | < 8 |  | 0·92 | 0·7 | 1·3 | 0·648 |  |  |  |  |  |
|  |  | ≥ 8 |  | 0·80 | 0·5 | 1·2 | 0·281 |  |  |  |  |  |
| Use soap to wash your hand | | No |  | 1 |  |  |  |  |  |  |  |  |
|  |  | Yes |  | 0·76 | 0·5 | 1·1 | **0·133** |  | 0·94 | 0·63 | 1·37 | 0·721 |

*p-value and odds ratio (OR) based on likelihood ratio test of univariate logistic regression, overall p-value of the models are indicated in bold letters. ** p-value and adjusted (a) OR based on likelihood ratio test of the multivariate regression model. The multivariate model was defined including exposure groups, sex, age, educational attainment, socioeconomic status, and number of people per household. In addition, all risk factors that had a p-value lower than 0.2 in the univariate analyses were included into the multivariate regression analysis (as indicated in the table). *** exposure groups: *com _exposed_*, slum dwellers at risk of flooding along the Nakivubo wetland; *com _comparison_*, slum dwellers without risk of flooding at least 2 km away from the Nakivubo wetland; *farmer*, urban farmers reusing wastewater within the Nakivubo wetland; *worker _ww_*, workers maintaining drainage channels and operating the Bugolobi Sewage Treatment Works; *worker _fs_*, workers managing fecal sludge (e.g., collection at households by means of vacuum trucks).
