# Supplementary material for: Risk of Intestinal Parasitic Infections in People with Different Exposures to Wastewater and Fecal Sludge in Kampala, Uganda: A Cross-Sectional Study
Source: PLoS Negl Trop Dis. 2016 Mar 3;10(3):e0004469. doi: 10.1371/journal.pntd.0004469 (PMC4777287; doi:10.1371/journal.pntd.0004469)
Supplement: S1 Protocol — (DOCX) [file pntd.0004469.s005.docx]

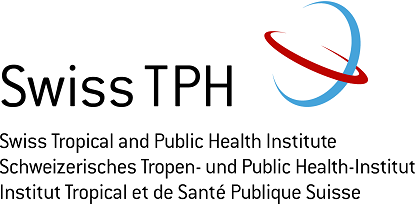


**EKBB 137/13, Version 2**

**Basel, 29.07.2013**

**Health risk assessment along the wastewater and faecal sludge chains: case study in Kampala**

Proposal for a PhD thesis in Epidemiology at the Swiss Tropical and Public Health Institute,

an associated institute of the University of Basel, Basel, Switzerland

Prepared by

**Samuel Fuhrimann**

Gartenstrasse 26

CH-4123 Allschwil, Switzerland

Mobile: +41 79 357-8424

E-Mail: samuel.fuhrimann@unibas.ch

**Supervision**

Prof. Dr. Guéladio Cissé, guealdio.cisse@unibas.ch

Dr. Mirko Winkler, mirko.winkler@unibas.ch

Prof. Dr. Jürg Utzinger, juerg.utzinger@unibas.ch

Department of Epidemiology and Public Health

Swiss Tropical and Public Health Institute, Basel, Switzerland

**Table of Contents**

[Abstract 4](#_Toc362689519)

[Acronyms and Abbreviations 6](#_Toc362689520)

[Operational definitions 6](#_Toc362689521)

[1.1 Background 8](#_Toc362689522)

[1.2 Statement of the problem 9](#_Toc362689523)

[1.3 Justification of proposed research 10](#_Toc362689524)

[1.4 Conceptual framework 10](#_Toc362689525)

[2. Literature review 12](#_Toc362689526)

[2.1 Resource recovery and reuse 12](#_Toc362689527)

[2.2 Waste management chain: from generation to disposal 12](#_Toc362689528)

[2.3 Hazards along the waste chain 13](#_Toc362689529)

[2.4 Control and mitigation measures 13](#_Toc362689530)

[2.5 Health risk assessment along the waste chain 14](#_Toc362689531)

[3. Goals, hypothesis and objectives 16](#_Toc362689532)

[4. Methodology 16](#_Toc362689533)

[4.1 Study site 16](#_Toc362689534)

[4.2 Study designs 17](#_Toc362689535)

[4.2.1 Study design for objective 1 17](#_Toc362689536)

[4.2.1.1 Study population 17](#_Toc362689537)

[4.2.1.2 Sample size calculations 18](#_Toc362689538)

[4.2.1.3 Sampling procedures 18](#_Toc362689539)

[4.2.1.4 Inclusion and exclusion criteria 21](#_Toc362689540)

[4.2.1.5 Sample analysis 21](#_Toc362689541)

[4.2.1.6 Study variables for the cross sectional survey 21](#_Toc362689542)

[*4.2.1.6.1* *Dependent variables* 21](#_Toc362689543)

[*4.2.1.6.2* *Independent variables* 22](#_Toc362689544)

[4.2.1.7 Data collection 22](#_Toc362689545)

[*4.2.1.7.1* *Training of research assistants* 22](#_Toc362689546)

[*4.2.1.7.2* *Tools: study questionnaire* 22](#_Toc362689547)

[*4.2.1.7.3* *Pre-testing* 23](#_Toc362689548)

[*4.2.1.7.4* *Field editing of data* 23](#_Toc362689549)

[*4.2.1.7.5* *Missing data* 23](#_Toc362689550)

[4.2.1.8 Data management and statistical analysis 23](#_Toc362689551)

[*4.2.1.8.1* *Data management* 23](#_Toc362689552)

[*4.2.1.8.2* *Statistical analysis* **Error! Bookmark not defined.**](#_Toc362689553)

[4.2.1.9 Expected outcomes 24](#_Toc362689554)

[4.2.2 Study design for objective 2 24](#_Toc362689555)

[4.2.2.1 Data collection 24](#_Toc362689556)

[4.2.2.2 Laboratory analysis of wastewater and faecal sludge 26](#_Toc362689557)

[4.2.2.3 Expected outcomes 26](#_Toc362689558)

[4.2.3 Study design for objective 3 26](#_Toc362689559)

[4.2.3.1 Model concept 26](#_Toc362689560)

[4.2.3.2 Expected outcomes 27](#_Toc362689561)

[5. Ethical considerations 27](#_Toc362689562)

[5.1 Ethical Committee Review 27](#_Toc362689563)

[5.2 Confidentiality 27](#_Toc362689564)

[5.3 Duties of the investigators 28](#_Toc362689565)

[6. Collaboration and support 28](#_Toc362689566)

[7. Time table 28](#_Toc362689567)

[8. Estimated budget 29](#_Toc362689568)

[9. References 29](#_Toc362689569)

[10. Annex 31](#_Toc362689570)

[10.1 Questionnaire 31](#_Toc362689571)

[10.2 Patient information and Consent Form 31](#_Toc362689572)

Abstract

**Background:** As all around the world, in Kampala, the recovery and reuse of both solid and liquid waste resources is of growing importance to urban lifestyles, although this sector is associated with various economic, social, environmental and health challenges for the approximately 1.5 million people living in the city. Uncollected or wrongly managed waste is a leading cause of soil, air and water pollution and it has potential of spreading infectious diseases. The current waste management and sanitation systems of Kampala struggle to catch up with the rapidly increasing amount of waste generated. There are examples of informal reuse practices around the ‘Bugolobi Sewerage Treatment and Disposal Works’ where wastewater and faecal sludge is treated. The dried sludge is reused as soil adamant and the treated wastewater is reused for unrestricted irrigation downstream of the plant within the Nakivubo swamp, where farmers grow root and leaf crops. Although, these recovered products are beneficial for urban agriculture, such practices might pose risks for human and animal health. Yet, to comply with the ‘WHO Guidelines for the Safe Use of Wastewater, Excreta and Greywater’, the policies and regulations have to be reviewed and adapted for the context of Kampala, and evidence on potential and effective risks have to be generated. Hence, there is a specific need and a scientific gap filling interest to undertake further assessments along the wastewater and faecal sludge chains linked to the Bugolobi area as a sanitation hotspot.

**Research objectives:** The goal of this PhD study is the generation of evidence on the exposure risk along the wastewater and faecal sludge chains in the perspective of potential promotion of the safe recovery and reuse of wastewater and faecal sludge in the context of Kampala city. The approach aims to specifically determine and quantify the health risk among exposed groups (workers and farmers) and to mitigate these health risks among potential affected people (consumers of products grown with wastewater and dried sludge and the general population) in Kampala city. The three specific objectives are: (i) to assess and map the health risk of specific groups exposed to wastewater and faecal sludge; (ii) to generate evidence on pathogenic contamination in wastewater and faecal sludge and establish a pathogen flow analysis along the two waste chains; and (iii) to conduct quantitative microbial risk assessments to simulate exposure scenarios for different groups exposed to wastewater and faecal sludge and to validate them with the data obtained under objective 1.

**Methods**: The study is composed of three parts: Part 1: a cross-sectional study with 1000 participants to assess the existing exposure risks due to wastewater and faecal sludge in directly exposed groups (includes 150 workers and 275 farmers), and partially and non-exposed groups as a control group (includes 575 communities members) with a specific focus on parasitic infections, skin diseases, eye diseases, and diarrhoeal episodes. The survey comprises of two components: (i) a questionnaire study to obtain health risk and health outcomes related to the exposure to wastewater and faecal sludge; and (ii) the collection of stool samples to determine the prevalence and the intensity of helminth (with the Kato-Katz method) and of intestinal protozoa infections (with the formalin-ether concentration technique); Part 2: a pathogen flow analysis to observe the variance of pathogen contamination at critical control points over time, we will assess existing operational monitoring data and analyse over a period of two month wastewater and faecal sludge for helminth eggs (modified Bailenger method) and intestinal protozoa cysts (formalin-ether concentration technique); and Part 3: a quantitative microbial assessment interlinked to the pathogen flow analysis to estimate diarrhoeal and parasitic infection risk and validate the results with the findings obtained under objective 1. Finally, with a subset of the stool and wastewater samples we will perform PCR for species detection and/or meta-genomic analysis in order to provide quantitative insights into microbial population based on sequence data.

**Expected results**: We will generate evidence and fill knowledge gaps on diseases outcomes related to the exposure to wastewater and faecal sludge. Based on these results we will be able to make evidence-based recommendations of the most appropriate combination of health protection measures to achieve context specific health base targets along the wastewater and faecal sludge chains and contribute to the sanitation safety plans processes. Hence, this study can help to promote the safe recovery and reuse of water and nutrients along these two waste chains and will benefit the planning of new wastewater and faecal sludge treatment plants and/or solutions in Kampala city.

Acronyms and Abbreviations

AIW Agro-industrial waste

BSTDW Bugolobi sewerage, treatment and disposal works

CCP Critical control points

Eawag Swiss Federal Institute of Aquatic Science and Technology

EKBB Ethikkommission beider Basel

FS Faecal sludge

FECT Formalin-ether concentration technique

HBT Health based targets

IWMI International Water Management Institute

KCCA Kampala Capital City Authority

MOH Ministry of public health in Kampala

MSW Municipal solid waste

NWSC National water and sewerage corporation

ODK Open data kit

PFA Pathogen flow analysis

QMRA Quantitative microbial risk assessment

RRR Recourse recovery and reuse

SANDEC Department of water and sanitation in developing countries

SDC Swiss agency for development and cooperation

SOPH School of Public Health in Kampala

SSP Sanitation Safety Plan

Swiss TPH Swiss Tropical and Public Health Institute

UNCST Uganda National Council of Science and Technology

WHO World Health Organization

WW Wastewater

Operational definitions

**Agro-industrial waste (AIW):** It includes a variety of waste products which arise at farm level. It can be plant material as well as animal excreta and remains.

**Business Model** **(BM)**: In this context we define it as a model that contributes to cost recovery or profit from reuse, ideally supporting in this way the sanitation service (e.g. wastewater treatment plant, faecal sludge management).

**Critical control points (CCP):** Is a point, step or procedure along a chain at which controls can be applied and hazards can be managed (prevented, eliminated or reduced) to reach tolerable risk levels.

**Cross-sectional study:** Is a descriptive study design to assess frequency and distribution of risk factors and/or disease outcomes. At one point in time a cross-section of the population at risk is sampled and examined.

**Dried Sludge**: Is found on drying beds where wastewater sludge from wastewater treatment plants is exposed to sun.

**Faecal sludge (FS):** Is raw (or partially digested) slurry or solid that results from the storage of black water or excreta. The composition of faecal sludge varies significantly depending on the location, water content and storage conditions. In general, faecal sludge can be distinguished between high strength (from latrines, non sewered public toilets) and low strength (from septic tanks).

**Hazard:** A biological, chemical, physical or radiological agent that has the potential to cause harm

**Health based target:** A defined level of health protection for a given exposure. This can be based on a measure of disease (e.g. 10^-6^ DALY per person per year, WHO standard for wastewater, excreta and grey water reuse), or the absence of a specific disease related to that exposure

**Log reduction:** Organism removal efficiencies: 1 log unit = 90%, 2 log units = 99%; 3 log units = 99.9% and so on.

**Municipal solid waste (MSW):** Definition by OECD (2010): “Municipal waste is collected and treated by, or for municipalities. It covers waste from households, including bulky waste, similar waste from commerce and trade, office buildings, institutions and small businesses, yard and garden, street sweepings, contents of litter containers, and market cleansing. Waste from municipal sewage networks and treatment, as well as municipal construction and demolition is excluded”.

**Pathogen flow analysis (PFA):** Is a mathematical model to analysis pathogen along critical control points, from source to human exposure. Removal (e.g. log reduction due to treatment), inactivation as well as re-growth of pathogen can be simulated over time.

**Quantitative microbial risk assessment (QMRA):** Is an approach that can estimate risks for a variety of different exposures and/or pathogens that would be difficult to measure through epidemiological studies. It comprises following four steps: (i) hazard identification; (ii) dose response assessment; (iii) exposure assessment; and (iv) risk characterization.

**Resource recovery and reuse (RRR):** Is a project which aims to promote the safe recovery and reuse of water, nutrients and organic matter from liquid and solid waste streams with a focus on the assessment of perspectives, opportunities and challenges of business models (BM) and sanitation safety planning.

**Sanitation Safety Plan (SSP):** Is practical step-wise oriented guidance to facilitate the development of locally site specific assessment and management plans to reduce health impact from wastewater, excreta and greywater, while at the same time maximize the benefits of their use in productive agriculture and aquaculture.

**Wastewater (WW):** All types of domestic, commercial and/or industrial effluent as well as storm water runoff, usually mixed and of different qualities, ranging from raw to diluted wastewater. The term does not imply any form of transport or treatment. It should be differentiated between raw wastewater and wastewater which entered natural water bodies (diluted wastewater, polluted stream water)

1. Introduction
   1. Background

This study protocol is part of a PhD thesis and describes the assessment of health risks related to the recovery and reuse of water and nutrients along the faecal sludge (FS) and wastewater (WW) chains in the context of Kampala.

The overall PhD thesis contributes to the larger framework of a project entitled ‘Resource recovery and reuse’ (RRR), which aims to promote the safe recovery and reuse of water, nutrients and organic matter from liquid and solid waste streams with a focus on the development and testing of a sanitation safety plan (SSP) and the assessment of perspectives, opportunities and challenges of business models (BM) (e.g. wastewater treatment plant and compost plant) which recover and reuse municipal solid waste (MSW), agro-industrial waste (AIW), FS and WW in general, and more specifically in the context of four selected cities, namely Kampala (Uganda), Hanoi (Vietnam), Bangalore (India) and Lima (Peru) .

The two key objectives of the RRR project are:

1. To increase the scale and viability of productive reuse of water, nutrients, organic matter and energy from domestic and agro- industrial waste streams through the analysis, promotion and implementation of economically viable BM.
2. To safeguard public health in the context of rapidly expanding use of wastewater, excreta and grey water in agriculture and aquaculture, and to protect vulnerable groups from specific health risks through health risks assessment and mitigation and SSP.

In Kampala the recovery and reuse of waste resources is of growing importance to urban lifestyles, although this sector is associated with various economic, social, environmental and health challenges. For example, uncollected or wrongly managed waste is a leading cause of soil, air and water pollution and it has potential of spreading infectious diseases (Kabatereine et al. 1997; WHO 2006a; Stenström et al. 2011; Hoornweg and Bhada-Tata 2012). In parallel, the waste management and sanitation systems of Kampala struggle to catch up with the rapid increasing rate of urbanisation and the resulting amount of waste generated (Beller consult et al. 2004; KCCA 2004). The sewage system in Kampala city, like in many African cities, is limited and reaches a maximum coverage of 6%. Hence, the large majority of the population relies on onsite sanitation facilities such as pit latrines and septic tanks. These pits are emptied via vacuum trucks (around 45 trucks are currently operating in Kampala) owned by public institutions such as KCCA and private cesspool operators, most of whom are members of the Private Emptiers Association (PEA) (Water and Sanitation Program 2008). The collected FS is disposed and treated together with the sewerage at the cities treatment plant ‘Bugolobi Sewerage Treatment and Disposal Works’ (BSTDW) which is operated by National Water and Sewerage Cooperation (NWSC). Due to the increasing volume of the FS disposed and the lack of improvement of the infrastructure of the treatment plant, the current design to handle the FS and sewerage is questioned and inadequate treatment became a common reality (Beller Consult et al. 2004; Water and Sanitation Program 2008; Kulabako et al. 2010). Due to such practises there are concerns that root crops (e.g. coco yams) and leaf crops (e.g. salads and vegetables) which are grown, for example in the Nakivubo swamp, contain harmful pathogens and chemicals. To underline these concerns, a study undertaken by Kayima et al. (2008) showed a high degree of pollution in Nakivubo channel which is caused by discharge of waste from various sources such as slums, markets and industries. The operational quality control of the WW undertaken by NWSC within the treatment plant and in the Nakivubo channel and swamp showed a significant level of pollution which is far above WHO standards for WW reuse in unrestricted irrigation (<10^3^-10^4^ *E.coli*/100ml) (WHO 2006). Moreover, the natural treatment function of the Nakivubo swamp is questioned in helping to bring down the level of pollution. In reality, the Nakivubo channel has become an open sewer and is steadily extended almost right through the entire Nakivubo swamp. Hence, the wastewater ends ether informally on the fields of the farmers within the Nakivubo swamp or is discharged into the inner Murchison Bay of Lake Victoria, which is one of the major sources of raw water and, therefore, deteriorates the drinking water quality of Kampala city (Fichtner Water & Transportation and M&E Associates 2008).

Furthermore, several studies indicate that soil-transmitted helminths (e.g. A*scaris lumbricoides*, hookworm and *Trichuris trichiura), Schistosoma mansoni* ((Brooker et al. 2009; Schur et al. 2011) and intestinal protozoa like Cryptosporidium (Howard et al. 2006) are highly endemic in the area around Lake Victoria. For instance, a cross-sectional survey undertaken among school children in Kampala estimated prevalences of *T.trichiura*, *A.lumbricoides* and hookworm at 28%, 17% and 12.9%, respectively, and highlighted the importance of dumped waste as a major source of transmission (Kabatereine et al. 1997). In Kampala there is currently only limited data on the presences of helminth eggs in WW and FS, and epidemiological studies about exposure risks for workers or farmers are lacking.

To improve the current situation, the Kampala sanitation master plan recommended to improve conventional WW treatment and anaerobic digestion for the production of biogas from the WW sludge in the Nakivubo area and to build three new decentralised treatment plants in Lubigi, Nalukolongo and Kinawataka. Moreover, the city authorities plan to improve the sewage coverage from 6% to 30% in 2033. This means, that on site sanitation using pit latrines and septic tanks will continue to be relevant until 2033 and probably also beyond and a more appropriate treatment solutions need to be developed (Beller Consult et al. 2004; Niwagaba 2009).

- 1. Statement of the problem

The health issues associated with occupational and environmental risks are of major concern in low and middle income countries where many people live and work in proximity to polluted wastewater streams, waste processing plants and disposal sites. For people whose livelihoods depend on the collection, on sorting and on reusing of waste, measures to improve their daily working conditions and to reduce associated health risks are needed. Various guidance documents in the grey and peer-reviewed literature provide developing country-specific recommendations to reduce the exposure to potential hazards and integrated control measures along the waste chain (Bünger et al. 2000; Hoornweg 2000; Cissé and Tanner 2001; Cissé et al. 2002; Rushton 2003; Cointreau-Levine 2006; WHOa-b 2006; Drechsel et al. 2010; Stenström et al. 2011; Hoornweg and Bhada-Tata 2012). On the other hand formal and save waste recovery and reuse practices are very limited in the low and middle income countries and often done informally and associated with health concerns, especially when the waste is reused in agriculture and aquaculture (WHO 2006a-d). For example, a study conducted on farmers who use wastewater in India had excess prevalence and intensity of infection compared to a control group (egg counts/g faeces) of ascariasis (47% vs. 13% and 69% vs. 31%, repectively) and hookworm infection (69% vs. 31% and 62% vs. 35%, respectively). Moreover, a review of epidemiological evidence on the health effects of wastewater and excreta use in agriculture conducted by Blumenthal and Peasey (2002) highlighted significant risks of gastro-intestinal infections to people working and living along the WW and FS chains.

In Kampala, as under chapter 1.1 briefly described, within the Nakivubo area such recovery and reuse of partially treated WW and FS takes place. For example, the dried wastewater sludge is reused as soil adamant and the nutrients are recovered in flower industries in agriculture. The treated effluent is informally reused for unrestricted irrigation downstream of the plant within the Nakivubo swamp, where farmers grow root (e.g. potatoes, cocoyam) and leaf crops (e.g. salad). Although, this recovered waste products are beneficial for agriculture, such practices might pose risks for human and animal health (Cissé 1997; Keiser and Utzinger 2005; WHO 2006a; Do et al. 2007). Yet, the potential risks have to be fully understood and policies and regulations, which are mainly relying on the ‘WHO Guidelines for the Safe Use of Wastewater, Excreta’ (WHO 2006a-d) have to be adjusted to the local context of Kampala. Therefore, it is important to undertake further assessments along the WW and FS chains and analyse the health risks related to this exposures in more detail. Hence, there is a pressing need to promote adequate risk mitigation measures for exposed groups (workers and farmers) and to give specific recommendations to adjust guidelines and regulation in the sanitation sector for the city of Kampala (WHO 2006a-d).

- 1. Justification of proposed research

The rationales of this investigation are centred around the existing need to fill specific knowledge and data gaps on effective health risks along the WW and FS chain. Hence, we want to establish a causal relationship between different level of exposures and disease outcomes related to WW and FS. The study results will add value to the overall sanitation planning and will inform and help to mitigate and minimize the health risks of the reuse of WW and FS and maximize the safe recovery of high valuable water and nutrient in Kampala city. Furthermore, the results can be incorporated in the SSP manual (Medlicott et al. 2012) and assist the pilot testing of the local SSP to step wise operationalize the WHO guidelines (WHO 2006) around the sanitation hotspot of the BSTDW in Kampala. The SSP pilot testing is conducted by a network of RRR project partners, locally the School of Public Health (SoPH), the Ministry of Public Health (MoH), NWSC, the Kampala Capital City Authority (KCCA) and various partners from Makerere University and internationally the International Water Management Institute (IWMI), Department of Water and Sanitation in Developing Countries (SANDEC) at Swiss Federal Institute of Aquatic Science and Technology (EAWAG) and Swiss TPH.

- 1. Conceptual framework

We will build up on the baseline health risk and impact assessment which will be conducted by SoPH and Swiss TPH along the WW, FS, MSW and AIW chains. This assessment will apply a methodological triangulation which will deploy a set of tools: (i) to collect and review at a large scale (i.e. urban, semi-urban and rural environments of Kampala) health statistic and literature (e.g. disease profiles, information on existing public health programmes and information on health-related national laws, regulations and guidelines); (ii) - at the level of existing waste recovery and reuse cases (e.g. BSTDW) - to assess the health risks by directly observing the working procedures at various critical control points (e.g. occupational health risk assessment matrix); and (iii) to understand the existing and acceptable mitigation measures by administering worker questionnaires, key informant interviews and community focus group discussions .

Hence, we want to add on top of this study a more detailed approach to better understand the related health risks and disease outcomes (e.g. diarrhoea episodes, skin diseases, helminth and intestinal protozoa infections) potentially caused by the exposure to WW and FS. This includes following three approaches (Figure 1):

1. The cross-sectional survey has the aim to assess the existing exposure risks due to WW and FS in directly exposed groups (worker and farmers) and partially and non-exposed groups (communities) with a specific focus on parasitic infections, skin diseases, eye diseases, and diarrhoeal episodes. The cross-sectional survey comprises of two components: (i) a questionnaire study to obtain health risk and health outcomes (e.g. diarrhoeal episodes and skin and eye disease) related to the exposure to WW and FS; (ii) and the collection of stool samples to determine the prevalence and the intensity of parasitic infections. We will analyse the stool samples with the Kato-Katz technique (1 stool sample subjected to duplicate Kato-Katz thick smears) for helminth infections and the formalin-ether concentration technique (FECT) for intestinal protozoa infections. A sub sample of Ascaris-positive specimens will be fixed in ethanol pending polymerase chain reaction (PCR) exanimation for species identification (*A.lumbricoides, A.suum*) (Becker et al. 2012).
2. A pathogen flow analysis (PFA) to observe the variance of certain contamination parameters (e.g. faecal coliforms) at critical control points over time. Therefore, we will analyse existing operational monitoring data of NWSC on WW and FS and sample WW and FS for analysis of helminth eggs and intestinal protozoa cysts.
3. We will interlink the PFA with a QMRA to estimate diarrhoeal and parasitic infection risk and validate the simulated results with the findings of the cross-sectional survey. This will further help us to calculate specific exposure scenarios for different reuse practices for WW and FS and to compare the results with local and international health based targets (HBT).


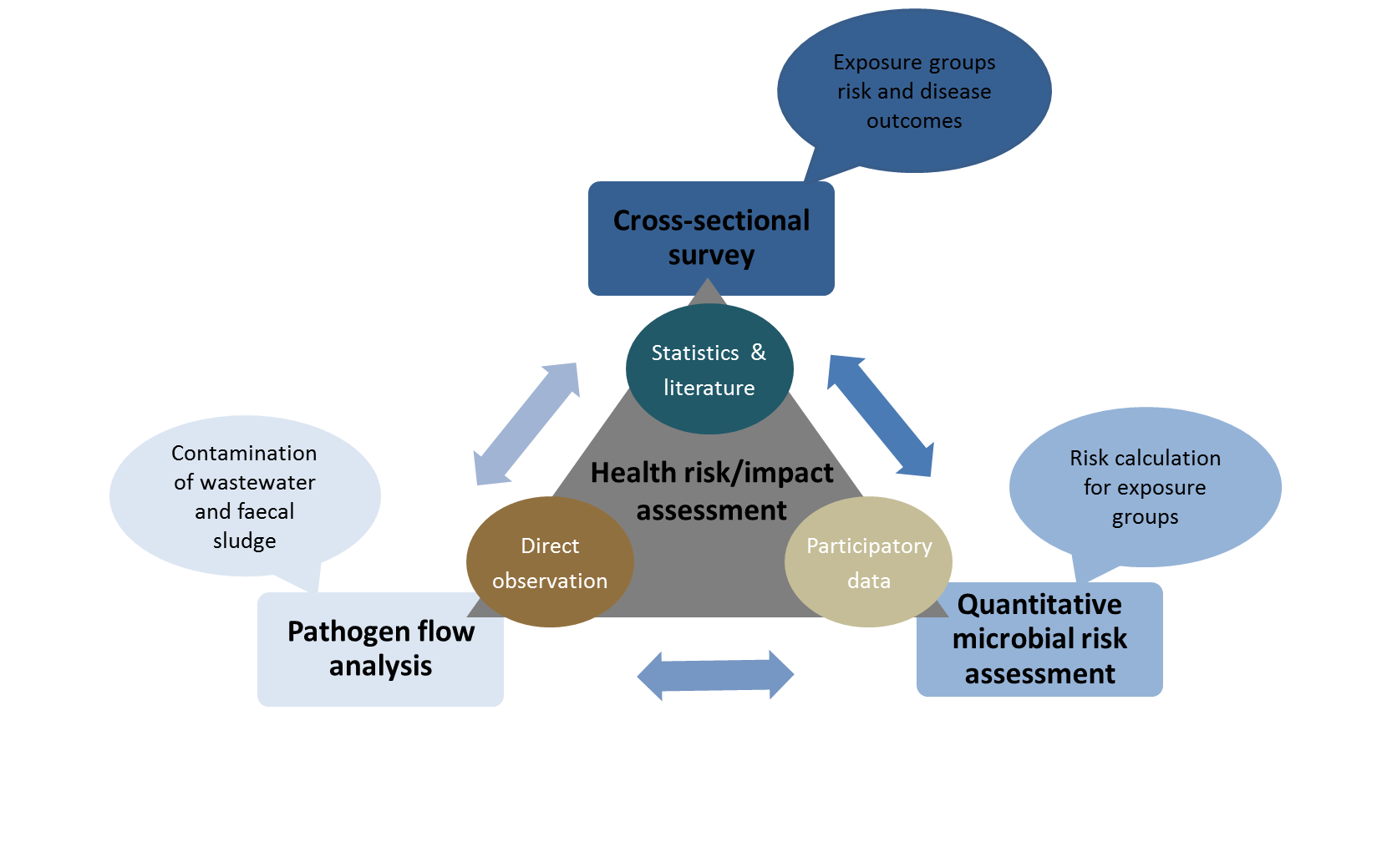


Figure 1 Conceptual framework of the proposed study along the wastewater and faecal sludge reuse chains in Kampala. The study builds on a baseline health risk and impact assessment, which deploys a methodological triangulation: (i) to collect and review health statistics; (ii) to assess the health risks by directly observing the working procedures at various critical control points, and (iii) to understand the existing and acceptable mitigation measures by administering participatory data collection tools. This study applies a more detailed approach to better understand the related health risks and disease outcomes: 1. a cross-sectional epidemiological survey with different exposure groups; 2. a pathogen flow analysis to look at contamination along the chains, and 3. a quantitative microbial risk assessment to simulate the health risks for various exposure groups.

1. Literature review
   1. Resource recovery and reuse

The recovery and reuse of resources classified as waste is of growing importance to urban lifestyles. At the same time, this sector is facing various economic, social, environmental and health challenges. For example, uncollected liquid and solid waste is a leading cause of soil, air and water pollution and acts as a source for the spreading of infectious diseases (Hoornweg and Bhada-Tata 2012). By 2025 half of the world’s population is predicted to live in water-stressed areas, with further enhanced competition for clean water, nutrients and energy (United Nations 2012). Hence, to recover and reuse valuable products from liquid and solid waste is of very high priority. Yet, waste management and sanitation systems of most developing countries struggle to catch up with a rapidly increasing amount of waste generated especially in urban and peri-urban areas (IWMI 2011).

The resulting, mostly inadequate, management of liquid and solid waste in urban and peri-urban contexts of low- and middle-income countries is leading to untreated disposal and subsequent human and environmental health risks (Cissé 1997; Strauss 2000). Reuse of waste is already occurring but is mainly restricted to the informal sector. As developing countries lack functional treatment plants and improved sanitation systems, waste recovery and reuse usually occurs in an unplanned way (Hoornweg 2000). For instance, the use of untreated wastewater in irrigated agriculture and aquaculture is widespread and risks for human and animal health have been documented (Cissé 1997; Keiser and Utzinger 2005; Trang et al. 2007). It is estimated that at least 20 million ha are irrigated with untreated wastewater, which cause considerable health risk to farmers and local communities (WHO 2006a; Drechsel et al. 2010). However, there are promising private innovative business models (BM) emerging the world over, which process waste resources to transform them into valuable goods. Many of the known recovery and reuse projects work on a small-scale and include low technology approaches such as anaerobic biogas production, aquaculture, aerobic composting, sludge fertilization and the utilisation of wastewater for irrigation (Hoornweg 2000; Nguyen 2005; Cointreau-Levine 2006; CIP 2007). The perspectives, opportunities and challenges of such projects have been observed, described and analysed in various studies (Strauss 2000; WHO 2006a; Seidu et al. 2008). There is growing consensus that resource recovery and reuse projects -when implemented at large scale- can fundamentally safeguard human and environment health and promote food security, cost recovery in the sanitation sector, and livelihood opportunities in urban and peri-urban areas of low- and middle-income countries (IWMI 2009)*.*

- 1. Waste management chain: from generation to disposal

Waste management and sanitation systems are previously described, for example in the Compendium of Sanitation Systems and Technologies (Tilley et al. 2008) and in the Microbial Exposure and Health Assessments in Sanitation Technologies and Systems (Stenström et al. 2011). The waste is handled and processed along multiple steps and comprises products (wastes) and functional groups which contain technologies. Tilley and Stenström are mainly concerned about WW and FS, which are processed through sanitation systems. Theoretical waste management templates can be developed by describing for each functional group various technologies which transforms and/or treats the waste to enhance the quality and safety of the products. The selection of the best technology is then context-specific and should consider hazards and hazardous events (e.g. cholera epidemic, trematode occurrence, heavy metal concentration), local health based targets (e.g. WHO guidelines and log reduction of a given technology), environment conditions (e.g. temperature, rainfall, moisture) and culture aspects and available resources (e.g. human behaviour and resources and input material) (WHOa-d 2006).

- 1. Hazards along the waste chain

Waste management bear increased health risk to a broad range of health hazards (e.g. diarrhoea, infectious diseases, injuries, toxic chemicals, etc.) to the exposed workers and the nearby communities (Cissé and Tanner 2001; Cissé et al. 2002; Rushton 2003; Ferrer et al. 2012). By looking at hazards associated with waste management and sanitation systems in low and middle income countries, one of the major health risk is associated with the cross contamination of human and animal excreta (WHO 2006a; Stenström 2011). Excreta include a broad range of pathogenic microbial and parasitic organisms of viral, bacterial, parasitic protozoan and helminth origins. Indeed, most of them are distributed with the faeces and urine of infected humans and animals, which are of the leading cause of gastro-intestinal symptoms such as diarrhoea, vomiting and stomach cramps (Ottosson 2003; Becker et al. 2012). In developing countries, a broad range of these pathogens which are potential excreted via faeces belong to the neglected tropical diseases and the control, treatment and diagnosis is limited (Utzinger et al. 2012). An overview of potential harmful hazards found in liquid waste is provided in Table 3.

Table 1. Examples of pathogens that can be excreted in faeces and transmitted via sanitation systems (Ottosson 2003; Keiser and Utzinger 2009; Becker et al. 2012).

| **Bacteria** |
| --- |
| *Aeromonas* spp*, Campylobacter jejuni, C. coli, Escherichia coli* (EIEC, EPEC, ETEC, EHEC), *Plesiomonas shigelloides, Salmonella typhi, S. paratyphi, Shigella* spp*., Vibrio cholera* O1 and O139*, Yersinia* spp*. , Enteric adenovirus* 40 and 41 |
| **Viruses** |
| *Astrovirus, Calicivirus (*Including *Norovirus), Coxsackievirus, Echovirus , Entervirus* types 68-71, Hepatitis A, Hepatitis E, *Poliovirus, Rotavirus* |
| **Parasitic protozoa** |
| *Giardia, Cyclospora, Cryptosporidium, Entamoeba* spp*.* |
| **Helminths** |
| *Ascaris lumbricoides, hookworms, Hymenolepsi nana, Strongyloides stercoralis, Toxocara canis, Trichuris trichiura, Taenia* spp. |
| **Trematodes** |
| *Clonorchis sinensis, Opisthorchis viverrini, O. felineus, Fasciola hepatica, F.* [gigantica](http://en.wikipedia.org/wiki/Fasciola_gigantica)*, Schistosoma* spp. |

Exposure to and resulting transmission of potentially harmful diseases can happen over various transmission routes at critical control points (CCPs) along the waste chain (Nguyen-Viet et al. 2009; Stenström et al. 2011) including (i) direct contact with contaminated food (e.g. food-borne trematodes (Keiser & Utzinger 2009)); (ii) accidental intake of waste or contaminated drinking water; (iii) inhalation of contaminated aerosols; (iv) indirect vector transmission (e.g. mosquitos and animal bite), among other pathways. The pathogenicity depends as well on environmental factors (persistence in the system over time), low infective dose (already a few organisms can result in an infection), the ability to induce human immunity, and the latency periods (infective first after a maturation period in the environment) (Stenström et al. 2011).

- 1. Control and mitigation measures

Control of health risks at CCPs can be achieved at every step along the waste chain from generation to disposal. Health risk is determined by the specific hazard prevalence in a given population. There is a need to find local solutions and adaptations of functional technological and non-technological mitigation measures (barrier function).

To reduce pathogens with technological barriers, the barrier function is usually expressed in log-terms and occurs through different adsorption or inactivation processes. For example, filtration and coagulation represents different adsorption processes, aerobic composting and anaerobic digestion are examples of microbiological inactivation processes, drying is the result of temperature and the regulation of pH, or the application of disinfection stands for physical and chemical processes which can reduce pathogen concentration (Beffa 2002; WHO 2006d; Stenström 2011).

Non-technological barriers are concerned with the practices related to the behaviour of local population regarding the handling and processing of waste and its products. It reflects the degree of exposure related to CCPs within the system and corresponding transmission routes. Hygienic behaviour varies according to the individuality, responsibility and practicality of the local population (Figure 1) (Carr and Strauss, 2001; Ekane, 2009).


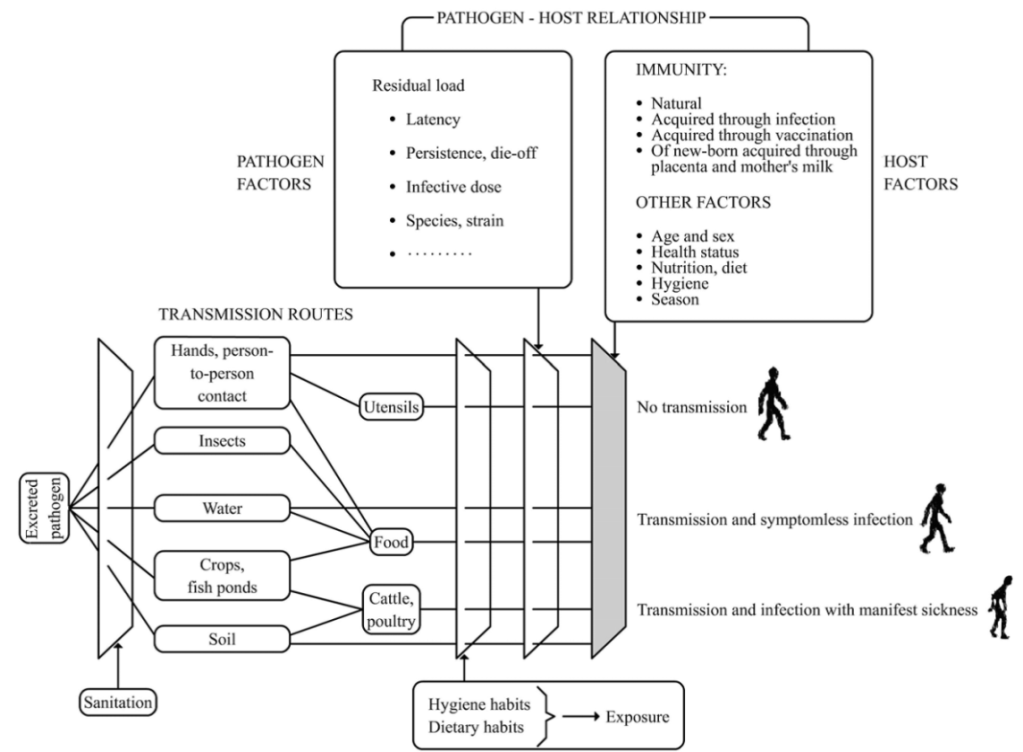


Figure 2. Examples of faecal oral pathogen transmission routs (Carr & Strauss, 2001)

A barrier function can be assessed by considering barrier efficiency (e.g. log reduction of pathogen achieved by a process), its robustness (e.g. by analysing the variation in reduction efficiency of pathogens and the technological malfunction) and the variability (its performance and compliance or non-compliance with certain practises). Introduced barrier functions should have the aim to reach a tolerable risk level associated with each identified health hazard. This risk level should be realistically implemented and enforced in a specific context and in line with existing local and/or international health-based targets or health standards (WHOa-d 2006).

- 1. Health risk assessment along the waste chain

To assess the health risks within a waste management system or sanitation system several approaches and tools can be used, includes classical epidemiological surveys, quantitative microbial risk assessment (QMRA), hazard analysis and critical control points (HACCP) or pathogen flow analysis (PFA), as summarised in Table 4.

Table 2. Description of tools and methods to assess the health risks of waste management and sanitation systems along CCPs (Cissé 1997; Cissé et al. 2002; Westrell et al. 2004; Nguyen-Viet et al. 2009; Surinkul et al. 2009)

| **Tools and methods** | **Description** |
| --- | --- |
| Epidemiology surveys | These surveys are based on a quantitative and qualitative risk assessment at population level to reveal how selected health parameters and risks factors distributed among different population groups. |
| Hazard analysis and critical control points  **(HACCP)** | Initially HACCP was developed for controlling food microbial hazards and is now increasingly utilised in the food safety control and in water treatment safety. It is following six principles to ensure the safety: (1.) conduct a hazard analysis; (2.) identify critical control points; (3.) establish critical limits for each critical control point; (4.) establish CCPs monitoring requirements; (5.) establish corrective actions; and (6.) establish procedures for ensuring the HACCP system is working as intended. |
| Quantitative microbial risk assessment  **(QMRA)** | Estimates the risk of infection in an exposed group, and can be extended to estimate the risk of disease. This allows the assessment of CCPs in food chains (production, transformation and consumption) and sanitation systems. This methodology is widely applied in risk assessment of drinking water and other practices, such as waste management. Recently, QMRA has been used to assess the risk of infection resulting in high risk of diseases for the population in contact with wastewater. |
| Pathogen flow analysis  **(PFA)** | The PFA focuses on most relevant pathways of pathogen transmission in the systems to quantify pathogen concentrations, pathogen flows and their respective reduction, inactivation or increase in different points of the environmental sanitation systems. The PFA approach will allow identifying the CCPs regarding pathogens to be tackled. |

There are also concepts which make use of these tools by integrating them into larger framework to approach and assess waste management from various sites. Hung et al. (2009) proposed a conceptual framework for improving health and environmental sanitation in urban and peri-urban areas, which combines a variety of tools to combine health, ecological, socioeconomic and cultural assessments. A project conducted by the Water and Sanitation Program (WSP) in cities in Indonesia proclaims a citywide sanitation strategy to plan process for sanitation sector development, which includes mapping of the existing condition using empirical data, which will serve as a foundation for devising the most effective strategy (WSP 2010). Further, a concept phrased Safety Sanitation Plan (SSP) is currently under development by the World Health Organization (WHO) and Swiss TPH in the framework of a new project called RRR. The SSP relies on the guidelines for the “safe use of wastewater, excreta and greywater” (WHO 2006a) and intends to promote a practical step-by-step way to analyse the sanitation system, reduce health risks and reach health-based targets from generation to disposal (Stenström et al. 2012).

1. Goals, hypothesis and objectives

The goal of this PhD study is the generation of evidence on the exposure risk along the wastewater and faecal sludge chains in the perspective of potential promotion of the safe recovery and reuse of wastewater and faecal sludge in the context of Kampala city. The approach aims to specifically determine and quantify the health risk among exposed groups (workers and farmers) and to mitigate these health risks among potential affected people (consumers of products grown with wastewater and dried sludge and the general population) in Kampala city. The study thus pursues the following three specific objectives:

1. To assess and map the health risks of specific groups exposed to WW and FS
2. To generate evidence on pathogenic contamination in WW and FS and establish a pathogen flow analysis along the two waste chains over time
3. To conduct quantitative microbial risk assessments to simulate exposure scenarios for different groups exposed to WW and FS and to validate them with the data obtained under objective 1

Deduced to the specific objectives we assume that increased exposure to wastewater and faecal sludge results in higher infection rates of parasitic diseases and other water-related diseases outcomes. Therefore we intent to test following two hypotheses:

1. The difference in odds ratio is 2.5 or higher between highly exposed groups (people working along the wastewater and faecal sludge chain) and the people without an exposure to the two waste chains (control groups).
2. The helminth infection intensity decreases with the distance to wastewater treatment plant in Bugolobi and the Nakivubo swamp.
3. Methodology
   1. Study site

Kampala is the capital and largest city of Uganda with a resident population of 1,597,500 people who live on an area of 197 km² (UBOS, 2010). This population nearly doubles during the day due to people who live in the neighbouring areas and travel to Kampala on a daily basis. The city has a [tropical climate](http://en.wikipedia.org/wiki/Tropical_wet_and_dry_climate) but because of the high altitude (1,190 m above sea level) the average temperature (16°C -28°C) is relatively cool compared to other lower altitude East African settings. The main rain season is between March and May and the short rain season is between November and December. Rainfall ranges between 500 mm and 2500 mm and the relative humidity is between 70% and 100% (Figure 1) (IWMI, 2012a).

The specific study site will be around the WW and FS treatment plant BSTDW and the Nakivubo swamp. It is located in an urban context of Kampala and surrounded by high density neighbourhoods in between Nakawa and Makindye divisions. The plant is operated by the NWSC and was constructed prior to the 1940s by the British and subsequently extended during the periods of 1956/1958. The WW entering the plant is treated via primary (grit and detritus removal and primary sedimentation), and secondary processes (effluent: trickling filter system secondary sedimentation; and WW sludge: anaerobic digestion ponds and drying beds). The secondary treated effluent is then led through a natural wetland (Nakivubo swamp) where it is tertiary treated before it is discharged into Lake Victoria (Figure 2). There are plans that the BSTDW is put out of service within the next 4-6 years, and replaced by a new plant. At the moment, NWSC and KCCA are conducting studies to determine the most appropriate treatment processes for this context.


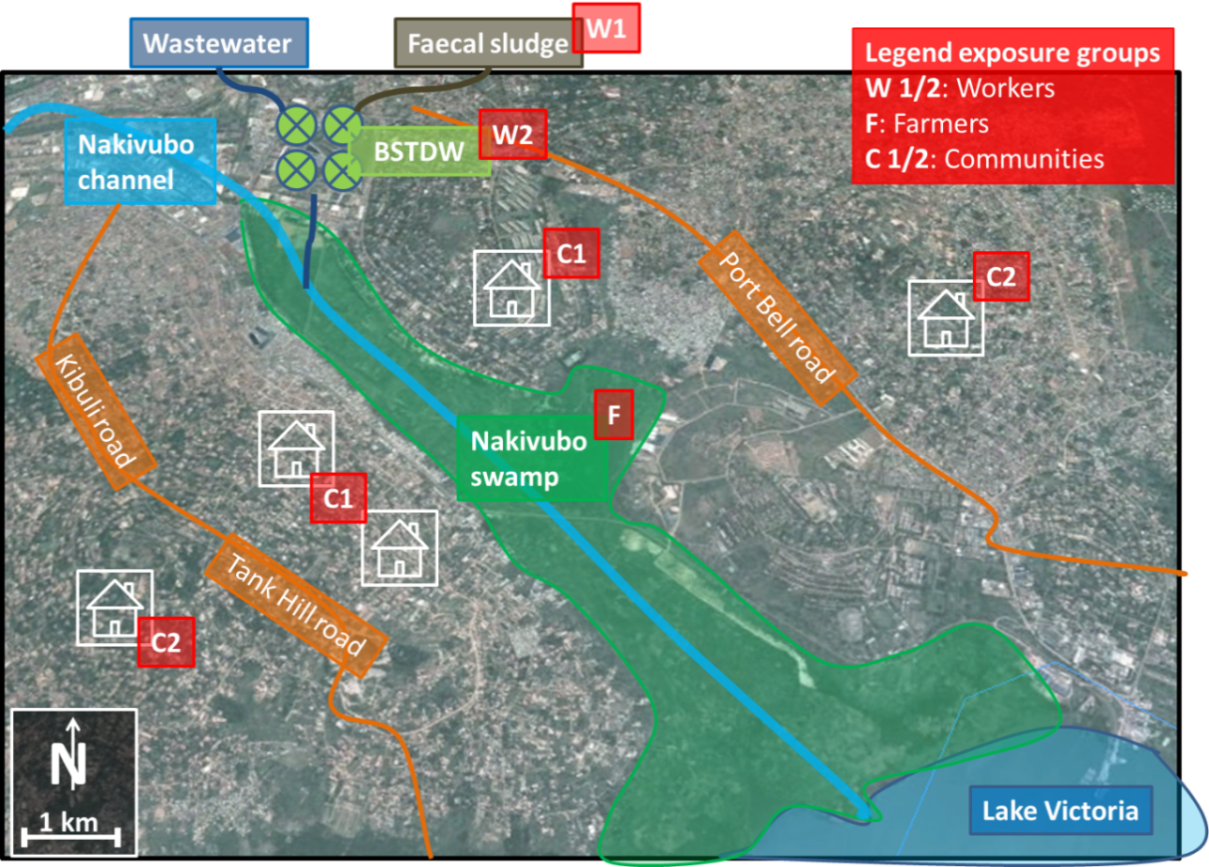


**Figure 3** Map of the area around the WW treatment plant ‘Bugolobi Sewerage Treatment and Disposal Works’ (BSTDW) and the Nakivubo swamp. Exposure groups are indicated with red squares. We assume that communities living approximately 2 km away from Nakivubo swamp and/or live ether east of the Port Bell road and west of Kibuli road and Tank Hill road are no longer exposed to wastewater and faecal sludge form BSTDE and Nakivubo swamp

- 1. Study designs
     1. Study design for objective 1

**Approach:** We will conduct a cross-sectional survey to assess and map the existing exposure risks due to WW and FS in directly exposed groups (worker and farmers) and partially and non-exposed groups (communities) with a specific focus on parasitic infections, skin diseases, eye diseases, and diarrhoeal episodes. The cross-sectional survey comprises of two components: (i) a questionnaire study to obtain health risk and health outcomes (e.g. diarrhoeal episodes and skin and eye disease) related to the exposure to WW and FS; (ii) and the collection of stool samples to determine the prevalence and the intensity of parasitic infections. We will analyse the stool samples with the Kato-Katz technique (1 stool sample subjected to duplicate Kato-Katz thick smears) for helminth infections and the formalin-ether concentration technique (FECT) for intestinal protozoa infections. A sub sample of Ascaris-positive specimens will be fixed in ethanol pending polymerase chain reaction (PCR) exanimation for species identification (*A.lumbricoides, A.suum*).

- - - 1. Study population

In the cross-sectional survey we will target in total five groups (three directly, one partially and one not exposed to WW and FS), namely: (i) workers operating the BSTDW (W1); (ii) workers collecting and transporting FS (W2); (iii) farmers working along the Nakivubo Channel and the wetland (F); (iv) community members who are exposed by WW and FS e.g. live in areas which are from time to time flooded but do no farming within the wetland (C1) and people therefore exposed to WW and/or FS; and (v) community members who live and work approximately 2 km away from the Nakivubo swamp, hence, we assume that these groups are not directly exposed to the WW and the FS (C2).

- - - 1. Sample size calculations

To calculate the sample size we relied on the hypothesis 1. We conducted a logistic regression analysis having four exposure levels W1/W2, F1, C1 and C2. The highest exposure level is assigned to W1 and W2 (due to their daily exposure to raw and untreated WW and FS), the second highest to F (due to accidentally intake of contaminated water and soil), the third highest to C1 (e.g. due to frequent flooding events) and the lowest to C2 (Blumenthal and Peasey 2002; WHO 2006a). The number of persons in category W1 and W2 is assumed to be limited to 150, the number of F1 to 250, the number of persons in class C1 to 200 while the number of persons in class C2 could be as high as necessary (Table 1).

**Table 3** Selection criteria and potential numbers of exposure groups

| **No.** | **Definition of exposure group** | **Estimated number of individuals** |
| --- | --- | --- |
| 1. **W1** | Worker who operate and work at Bugolobi Sewerage Treatment and Disposal works | 30-50 |
| 1. **W2** | Worker who collect and transport the faecal sludge | 100-150 |
| 1. **F** | Farmers who working within the Nakivubo swap and are exposed to WW and/or FS | 250-500 |
| 1. **C1** | Community in proximity of the wetland but are no farmers | 10,000-20,000 |
| 1. **C2** | Community who live and work approximately 2 km away from the Nakivubo swamp and live ether east of the Port Bell road and west of Kibuli road and Tank Hill road. | 10,000-20,000 |

We aim at a power of 95%, in order to make sure that a reduction in effective exposure variance by 35% still leaves 80% power. Effective exposure variance is the variance of the 4-level exposure variable after regression on potential confounding variables (e.g. socio-economic status). With a variance reduction by 35%, the remaining exposure variance is 65% of the original value, so that the standard deviation after reduction is about 80% of the original exposure standard deviation. This increases the standard error by a factor of 1.25. If we have 80% power with the adjusted standard error (SE) 1.25*SE we may detect an effect of ln(odds ratio (OR)) = (1.96+0.84)*1.25*SE = 3.5*SE = (1.96 + 1.54)*SE. And 1.54 corresponds to slightly less than 95% power with the original unadjusted SE. Clearly, the factor limiting power most strongly is the relatively small size of the highest exposure group (W1, W2).

**Table 4** Results of the logistic regression analysis considering four exposure variable (C1, C2, F1 and W1)

| Exposure Scenarios | Assumed prevalence rate for C2 | Assumed difference in Odds Ratio between W1/W2 and C2 | Assumed prevalence rates in  C1 / F / W1,W2 | Calculated sample size for each group  C1 / C2 / F1 / W1,2 |
| --- | --- | --- | --- | --- |
| **1** | 40% | 2 | 46 / 51 / 57 | 300 / 200 / 250 / 150 |
| **2** | 30% | 2 | 35 / 40 / 46 | 400 / 200 / 250 / 150 |
| **3** | 20% | 2.5 | 25 / 32 / 38 | 300 / 200 / 250 / 150 |

To anticipate for non-response or missing data, a margin of 25 participants for F, C1 and of 50 for C2 is added resulting in a final sample size of 1000 participants

In view of these considerations and calculations we will plan to conduct our study with the sample size calculated for the exposure scenario 3 (Table 2) and target the following number of people in each group: C1: 350, C2: 225, F1: 275 and W1 and W2 an excessive sample of 50 and 100, respectively and assume a prevalence rate of intestinal parasitic infections of 20% ( Kabatereine et al. 1997; Blumenthal and Peasey 2002;; WHO 2006b) in the lowest exposure group (C2) and an OR between the highest (W1, W2) and lowest exposure group (C2) of 2.5.

- - - 1. Sampling procedures

First, we will sensitise the worker (W1 and W2) study groups while meeting with representatives of each group (WW treatment plant operator, chairman of FS workers) and explain the aims and procedures of the study. Each of the representatives will be asked to prepare a list that contains the names, sex and age of their group members. We aim to select an excessive sample of the two groups approximately 50 for W1 and 100 for W2.

To select the community groups C1 and C2 we will rely on the population data collected in the census 2012 and characterise the low and middle income settlements restricted to an area around the Nakivubo swamp (population estimate and area characterization for C1 is given in Table 5 and Figure 4). We will focus at a total area of approximately 96 km^2^ and subdivide the area in 96 squares (each square 1km^2^) and exclude all areas which belong to ether high income, industrial or business areas. Further we will describe and map the characteristics (income level, numbers of household) of each square and assign it to ether C1, C2. To get the necessary data we will first consult the data from the national bureau of statistic to have a rough understanding of the social-economic pattern, second our team will go and map the households and estimate the number of people living in each square. Later, we will randomly select (with random numbers) 14 squares for C1 and 9 squares for C2. Within each square we will randomly select 25 households and contact the household members personally to ask if they are willing to participate in the study. If they agree we will make an appointment for an interview and will collect the stool sample the next morning (Figure 5).

Table 5 Population sizes of communities (C1) exposed to Nakivubo swamp

| Area | Zones | Social economic status | Population estimate |
| --- | --- | --- | --- |
| Bukasa parish | Keityabya/Yoka | Low | 5,000 |
|  | Soweto/Kanyogoga | Low | 2,500 |
|  | Namuwongo A | Low | 1,500 |
|  | Namowongo B | Low | 3,000 |
| Muyenga |  | High | 1,500 |
| Nakawa | Port Bell | Low | 3,000 |
|  | Kitintale | Middle | 2,000 |
|  | Bugolobi | Middle | 2,000 |
|  |  | Total | 20,500 |


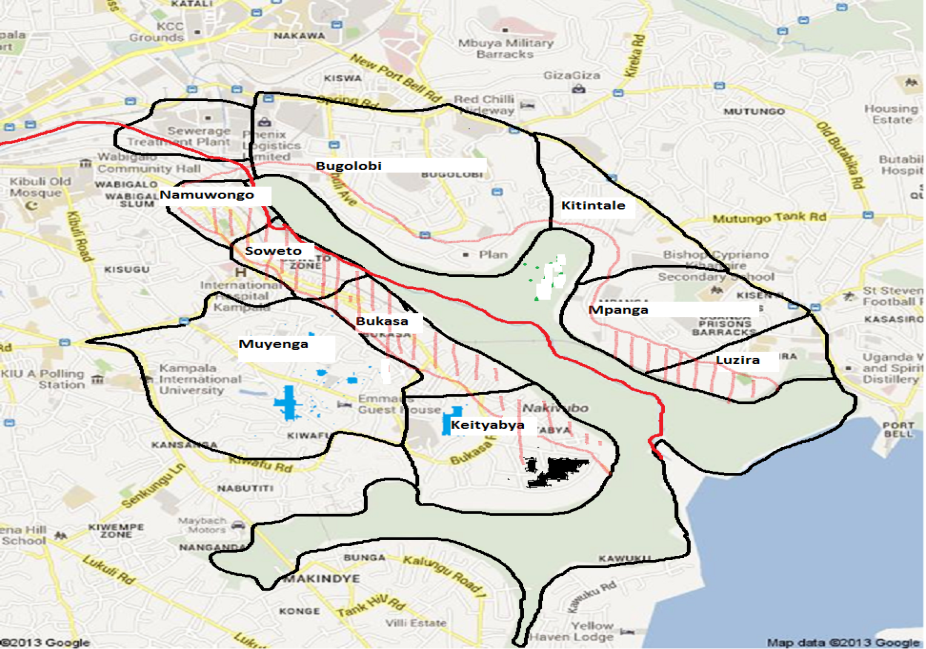


**Figure 4** Map showing communities adjacent to Nakivubo swamp

To select farmers (F1) we have to first describe and map the on-going farming activities within the Nakivubo swamp and channel. To estimate the number of farmers our research team will compile a list with the contact details (name, phone number and GPS coordinates where the framing activity takes place) of farmers who would be interested to participate in this study on two successive days, once in morning between 9-11 am and once in the afternoon 2-4 pm. Later we will randomly select 275 framers from this list and contact them to arrange an individual meeting to inform them about the study objective. If they agree to participate, we will make an appointment for interviewing and collect the stool sample the next morning (Figure 5).


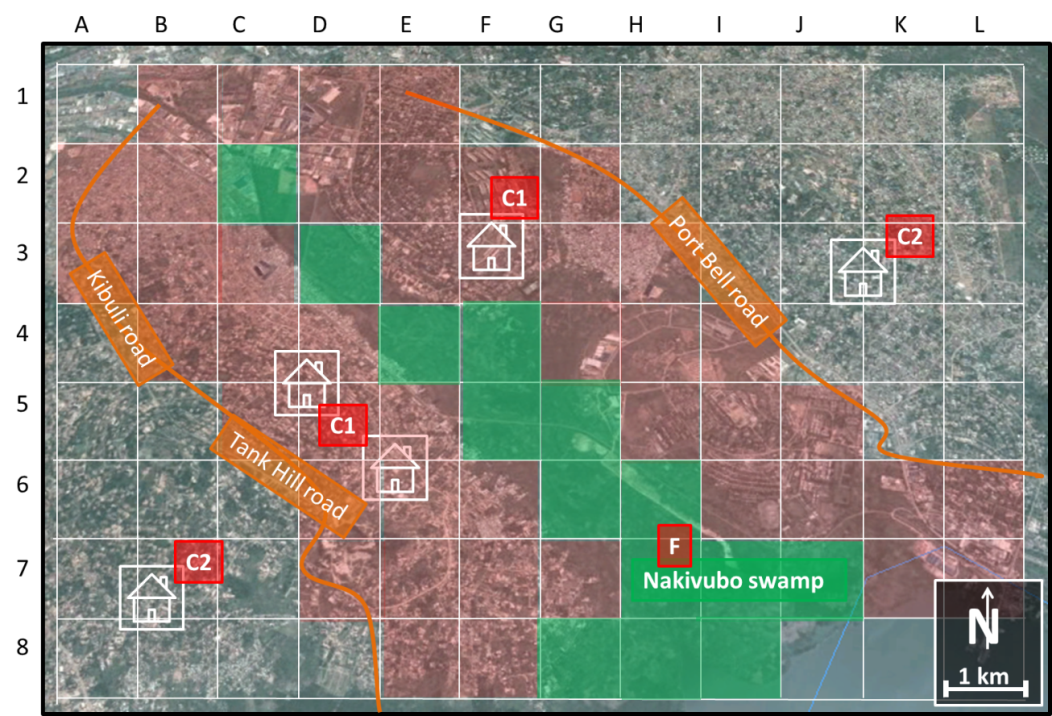


**Figure 5** Grid-system for the selection of community and farmer groups in the area around the Nakivubo swamp. Red squares indicate **(C1)** communities which are potentially exposed by WW and FS e.g. live in areas close to the Nakivubo swamp, transparent squares indicate **(C2)** communities which live approximately 2 km away from the Nakivubo swamp, hence, we assume that these groups are not directly exposed to the WW and the FS and green squares indicate **(F)** areas within Nakivubo swamp where farming takes place.

Each of the selected study participants will be informed on the study objectives, procedures, and potential risks and benefits. People will be invited to participate in the study. As re-emphasized under the chapter ‘Ethical considerations’, persons who are interested to participate in the study will have to sign an informed consent (ANNEX 10.2). We will only select persons aged 18 years and above.

Once the written informed consent has been obtained, each participant will get a unique ID (4 digits e.g. 0001; (i) 1-5, which indicates the exposure group (1 = W1; 2 = W2; 3 = F1, 4 = C1, and 5 = C2); (ii-iv) 1-350 which indicates the individual in the group).

Further, we will conduct with each participant a questionnaire interview (will take about 30 min) to assess e.g. their demographic, occupation, intensity and causality of exposure to WW and FS, personal protective equipment, hygienic behaviour and signs and systems of specific health outcomes (ANNEX 10.1). Later, each participant will be provided with a small plastic container which is labelled with their unique ID. Participants will be invited to return the container with a fresh morning stool sample the following day directly to the working place for W1 and W2 and for community member and farmer to an appointed health centre. The anticipated study period for W1 will be 2 days, for W2 5 days, for F 10 days, for C1 5 days and for C2 10 days.

Table 6 Sampling framework for the cross-sectional study

| Exposure group | Total participants | Allocated working days | Selected unit | Participants per unit |
| --- | --- | --- | --- | --- |
| **Worker 1** | 50 | 2 | Excessive sample | 50 workers |
| **Worker 2** | 100 | 10 | Excessive sample | 100 workers |
| **Farmers** | 275 | 10 | 275 randomly selected farmers from pre-compiled list |  |
| **Communities 1** | 225 | 5 | 9 squares (1km^2^) | 25 households (one member per household) |
| **Communities 2** | 350 | 10 | 14 squares (1km^2^) | 25 households (one member per household) |

- - - 1. Inclusion and exclusion criteria

Wastewater treatment plant workers, faecal sludge workers, farmer and community members will not be allowed to participate in the study if they do not fulfill any of the following exclusion criteria:

- too sick to attend school or participate in the study (e.g. severe diarrhoea, severe anaemia, high fever, etc.)
- absence of written informed consent
- person is younger than 18 years of age
  - - 1. Sample analysis

The collected stool samples will be transferred to the laboratory of the Vector Control Division of the MOH on the same day for examination of helminth eggs and intestinal protozoa cysts.

For each stool sample duplicate Kato-Katz thick smears will be prepared, using standard 41.7 mg template. The slides will be labelled with the participants unique ID plus ether ‘A’ (first Kato-Katz) or ‘B’ (second Kato-Katz) and allowed to dry for at least 30 minutes. Slides will be examined quantitatively under a light microscope by 1 laboratory technicians. For each slide the number of helminths (*A.lumbricoides*, hookworm, *T.trichiura* and *S.mansoni* and other helminth) will be counted and recorded separately.

Additionally, for each stool sample we will perform a FECT to detect protozoa infections. In short, approximately 1 g of the stool will be placed in a tube containing 10 ml of formalin and labelled again with the unique ID number of the participant plus a C. The sample will be mixed, centrifuged and the supernatant removed. 7 ml saline solution will be added, mixed and centrifuged. The sediment is the examined on presence of helminth eggs and intestinal protozoa cysts under a light microscope.

For quality control 10% of all the Kato-Katz slides will be re-examined by an external person (e.g. PhD student), the results of the *A.lumbricoides, T.trichiura* and *S.mansoni* egg counts compared and any discrepancies removed by re-examining the discordant slides.

A subset of the stool samples (100-150 samples) will be frozen (-20°C) and transferred to Swiss TPH in Switzerland for pending polymerase chain reaction (PCR) for species detection and/or meta-genomic analysis in order to provide quantitative insights into microbial population based on sequence data.

- - - 1. Study variables for the cross sectional survey
         1. *Dependent variables*

As dependent variables we will measure the prevalence of all detected parasitic infection (helminth and intestinal protozoa) and the intensity of helminth infections in the stool samples. For the questionnaire study we will ask the participants questions about symptoms and signs of certain disease outcomes to estimate the prevalence of fever and skin, eye, reparatory diseases and diarrhoea episodes within a two week recall period.

- - - - 1. *Independent variables*

As independent variables we ask the participants questions around their perceived exposure risk to WW and FS, their occupation, their working procedures, the personal protective equipment they use to reduce the exposure and their hygienic behaviour. To check for confounder we also ask the participants about demographic parameters (e.g. age, gender), behaviour, consumption of water and root and leaf crops and socio-economic status (ANNEX 10.1).

- - - 1. Data collection
         1. *Training of research assistants*

To accomplish the questionnaire survey, the stool sampling and we will train 4 local research assistants on the interview procedure and on the usage of the tablet computer.

To conduct the laboratory analysis we intend to collaborate with the already qualified staff of the lab of the MOH and the laboratory of the NWSC.

- - - - 1. *Tools: study questionnaire*

The questionnaire will be developed in English on paper (ANNEX 10.1) and later translated by the local partners from SoPH into the local language. Further, the questionnaire will be translated into the program code of an open-source software called Open Data Kit (ODK) (<http://opendatakit.org>), which enables us to conduct the questionnaire survey with tablet computer devices. Hence, after pre-testing we will adjust and refine the questions and the questionnaire tool to the local context.

The questionnaire interview will last for about 30 minutes and comprises of the following 10 chapters:

1. **Background information**: (i) to get information on the location of living and working, directly measured with the GPS tracking function of the table computer and later mapped with the ESRI *ArcGIS* 10 computer programme; (ii) to obtain information about potential confounding factors: gender, age and religion and ethical affiliation of the participant.
2. **Occupational back ground information:** to assess (i) information on the exposure due to occupation we ask questions to about their occupation and the exposure to Nakivubo swamp and/or channel (only for the groups C1 and C2); (ii) the intensity of occupational related exposure information around the employment, employer, function and responsibilities and working days/hours of the current occupation; and (iii) the potential positive effect on health due to received safety training and regular health check-ups.
3. **Socio-economic status:** to assess the socio economic status as potential confounding factor we will obtain information around the salary per month, amount of people living in the household and on property possession.
4. **Hygienic behaviour:** to assess personal hygienic behaviour as potential confounding factor we will ask questions around washing/showering and hand washing.
5. **Consumption of water and food:** to assess the consumption of contaminated food and water as potential confounding factor we will obtain information around: (i) consumption and treatment of drinking water from various sources; (ii) consumption and concerns of root and leaf crops; and (iii) if participant is undertaking any farming what kind of crops he/she is growing.
6. **Disease signs and symptoms:** to observe signs and symptoms of diseases participants experienced over the past 2 weeks we will ask questions: (i) about general diseases symptoms (headache, fever, abdominal pain, acute and chronic coughing, chest pain, loss of weight, nausea, vomiting), physical pains (back, joint and muscle pain) and injuries, (ii) we will ask more specific questions around water-washed infection to determine skin and eye diseases as defined in the ‘Oxford handbook of tropical medicine’ (Eddleston et al. 2012). For skin diseases we will also ask for the specific part of the location (e.g. arm and leg); and (iii) questions on diarrhoeal episodes (defined as three or more loose or liquid stools per day) experienced today, 48 hours ago, 7 days ago and 14 days ago. Additional questions will be asked in order to characterize the diarrhoea such as presence of blood and mucus.
7. **Risk perception:** to assess the awareness of the health risk due to the work and the exposure to wastewater and faecal sludge.
8. **Personal protective equipment (PPE) (only W1/W2 and F):** to assess the use, the compliance and the efficacy of specific PPE.
9. **Worker safety (only W1/W2 and F):** to assess the need for other PPE and measures/controls that should be in place for making the work safer.
10. **Stool collection:** At a this stage of our research we want to ask the participant if he/she is willing to provide a stool sample for assessing the burden of intestinal parasites (e.g. soil-transmitted helminth and intestinal protozoa). If yes, we would measure the weight and height to calculate the body mass index to provide the appropriate dose of treatment in case of positive laboratory analysis.
    - - - 1. *Pre-testing*

To pre-test the questionnaire interviews with the tablet computer and the collection and analysis of stool samples in Kampala, Uganda. Each assistant will interview 3 people who work in the office or lab of BSTDW. The next day we will collect stool samples from the interviewed people and analyse it at the lab of the MOH.

- - - - 1. *Field editing of data*

Data will be entered directly into the data entry mask of the ODK from the assistants during the interviews.

- - - - 1. *Missing data*

Participants are free to not answer any questions or provide a stool sample, in such a case the data entry field will be marked as missing. To anticipate for non-response or missing data, a margin of 25 participants for F, C1 and of 50 for C2 is added resulting in a final sample size of 1000 participants.

- - - 1. Data management and statistical analysis
         1. *Data management*

Every evening the data collected with the tablet computers will be synchronized with a secure server from Swiss TPH in Switzerland where only the main investigators have access to. A random sub sample (100-150 samples) of the collected stool samples will be completely anonymized and transferred to Switzerland for pending polymerase chain reaction (PCR) for species detection and/or meta-genomic analysis in order to provide quantitative insights into microbial population based on sequence data.

- - - - 1. *Statistical analysis*

First, data cleaning will be carried out using STATA version 12. In a next step, descriptive statistics will be used to calculate the distribution of the above mentioned dependent and independent variables within each exposure group.

A first analysis is to test hypothesis 1. Hence, a multilevel regression analyses, adjusting for confounding, will be used to measure the associations between dependent variables (disease outcomes) and independent representing the exposure to WW and FS and to calculate Odds ratios to measure the strength of the associations and to compare the exposure groups.

A second multilevel regression analysis aims to test the hypothesis 2, which expects a potential association between helminth egg counts and the distance to the water treatment plant and the Nakivubo swamp. We will define x as equal the distance from water treatment plant and Nakivubo swamp (1 = maximal distance and 0 = living or working directly at the plant or Nakivubo swamp). A true difference in average logarithmized egg counts of 0.8 individual standard deviations between subjects with x = 0 and subjects with x =1 would guarantee 95% power of finding a statistically significant association and would leave room for covariate adjustment. To verify the hypothesis we would assume that a difference of 0.8 standard deviations corresponds to a situation where the median egg count of the highest exposure group (with x=0) is at the 80^th^ percentile of the lowest exposure group (with x = 1).

- - - 1. Expected outcomes

With the cross-sectional study approach will create scientific evidence on the exposure risk to the WW and FS and are able to map the health risk along two waste chains in the Nakivubo area. Hence, we will test if the assumption made in hypothesis 1 and 2 are true to verify or falsify the hypothesis.

- - 1. Study design for objective 2

**Approach:** We will establish a PFA along the WW and FS chain around the BTWP and the Nakavibu swamp in Kampala. We will follow the pathogen concentration of faecal coliform, helminth egg and intestinal protozoa cysts over time.

- - - 1. Data collection

To establish a PFA we intent to use as a backbone of the model secondary data (e.g. obtained during the routine quality data collected by the BTWP (Table 3)) and will conduct a trend analysis for the pathogens and contaminations of interest (faecal coliforms, pH and other relevant parameters which can be obtained) over time. Additionally, we will analyse the WW and FS over a period of 2 months for helminth eggs and intestinal protozoa cysts.

**Table 7** Quality testing of WW at various sources within and around the Bugolobi sewerage, treatment and disposal works

| **Reported Data: 09.01.2013** | **Standard** | **Raw sewage** | **Final effluent** | **Nakivubo channel** | **Nakivubo wetland** |
| --- | --- | --- | --- | --- | --- |
| Ammonia –N (mg/l) | **10** | 43 | 12 | 8 | 10 |
| BOD_5_ (mg/l) | **50** | 465 | 122 | 134 | 288 |
| COD (mg/l) | **100** | 889 | 239 | 258 | 266 |
| Conductivity (nS/cm) | **1’500** | 1’049 | 1’097 | 998 | 1’025 |
| Faecal coliform (CFU/100 ml) | **10’000** | 52’000’000 | 68’000 | 134’000 | 142’000 |
| pH | **6.0-8.0** | 8 | 8 | 8 | 8 |
| Ortho Phosphate (mg/L) | **5** | 13 | 7 | 9 | 10 |
| Total Phosphate (mg/L) | **10** | 13 | 7 | 10 | 10 |
| Total Alkalinity (mg/l as CaCO_3_) | **700** | 480 | 460 | 400 | 420 |
| Total Suspended Solids (mg/l) | **100** | 788 | 74 | 395 | 450 |

We will sample over a period of two month 1 l of WW twice a week and three times per day (once between 9-10 am, 2-3 pm and 5-6 pm)) to take into account the daily variance. The WW and FS samples will be taken form five sources, namely: (i) raw sewage; (ii) final effluent; (iii) raw effluent of the FS; (iv) water of the Nakivubo channel; and (v) water within the Nakivubo swamp (Figure 3). In addition we also want to analyse the dried sludge in the drying beds. Therefore, we will take samples from five points in five drying beds twice a week over a period of 2 months (Figure 4).

All the samples will be collected and prepared (sedimentation and centrifugation) by lab personal of the BSTDW and handed over to the laboratory of the vector control division of the MOH for further preparation and analysis of the pathogens as described below.


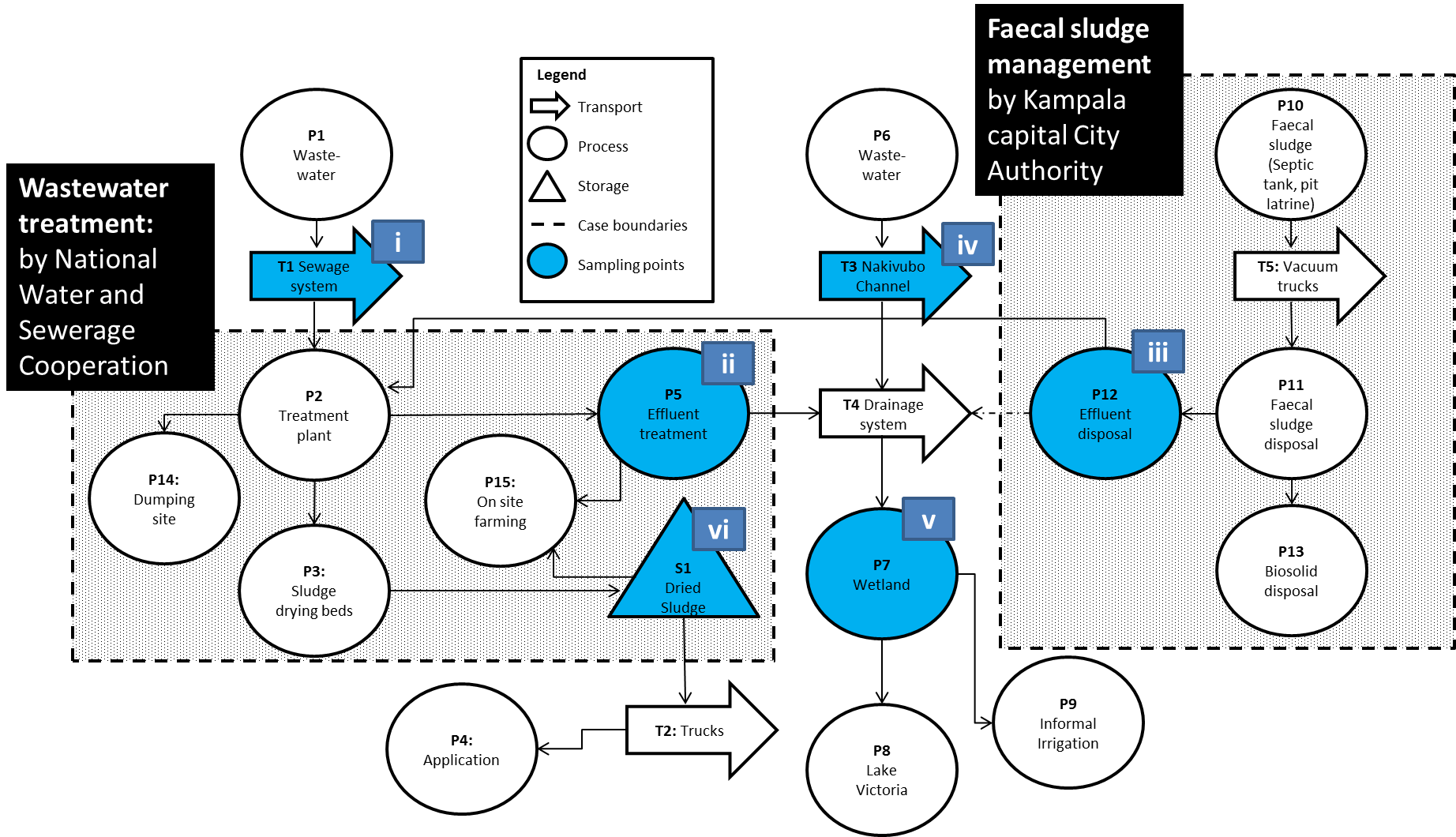


**Figure 6.** Simplified process flow diagram of the study site around National Water and Sewerage Corporation in Bugolobi. Blue colour indicates the sample points for the testing for helminth eggs and intestinal protozoa cysts. (i) raw sewage; (ii) final effluent; (iii) raw effluent of the faecal sludge; and (vi) Nakivubo channel and (v) in the Nakivubo swamp and (vi) dried sludge.

- - - 1. Laboratory analysis of wastewater and faecal sludge

To analyse the various WW samples and the FS effluent we will use the modified Bailenger method (Ayres et al. 1996) which is recommended by WHO to analyse WW. With this method we can successfully recover a wide range of helminth eggs including *A.lumbricoides, T.trichiura*, *Capillaria* spp., *Enterobius vermicularis*, *Toxocara* spp., *Taenia* spp., *Hymenolepis* spp., and hookworm eggs. The procedure involves sedimentation of the sample for about 1 hour, centrifugation of the sediment and extraction with ether. Later it is mixed with zinc sulphate solution and applied on a McMaster slide for examination of helminth eggs under a microscope.

For the analysis of the dried sludge we will use the same Kato-Katz technique and FECT technique previously described for the examination of the stool samples for helminth eggs or for intestinal protozoa respectively.

A random sub sample (100-150 samples) of the collected wastewater and faecal sludge samples will be transferred to Switzerland for pending polymerase chain reaction (PCR) for species detection and/or meta-genomic analysis in order to provide quantitative insights into microbial population based on sequence data. The pathogen sequence data found in the wastewater and faecal sludge are then further compared with the pathogen sequence data found in the stool samples of the participants of the cross-sectional survey.

- - - 1. Expected outcomes

The outcomes of the PFA will indicate specific hazards at critical control points along the WW and FS chains over time. This helps us to determine a correlation between the pathogen concentrations and the disease outcomes obtained under objective 1 in exposure groups along the WW and FS chain.

- - 1. Study design for objective 3

Approach: We will use the data on faecal coliform, helminth egg (e.g. *A.lumbricoides* egg) and intestinal protozoa cyst (e.g. *Cryptosporidium parvum* cyst) concentrations determined for specific critical control points in the PFA and interlink them with a QMRA.

- - - 1. Model concept

Haas et al. (1999) described QMRA as an iterative approach in the following four steps to estimate health risks: (i) hazard identification (identify relevant pathogens, in our case faecal coliforms, *A.lumbricoides*, *C.parvum*); (ii) dose-response assessment (Relationship between doses and negative health effect on exposed population, data for the selected pathogen can be accessed in the literature); (iii) exposure assessment (estimate frequency, amount and duration of exposure for relevant transmission routes, will be assessed in the interviews with exposed groups); (iv) risk characterization (estimate public health risk, and evaluate uncertainty and sensitivity).

We will develop different exposure scenarios (e.g. involuntary soil or wastewater ingestion) (WHO 2006b; Mara 2007; Mara 2010) to estimate the theoretical *A.lumbricoides* egg and *Cryptosporidium parvum* infection risk and diarrhoeal risks to for different exposed groups. Therefore we will run series risk simulation using a QMRA-Monte Carlo computer program (Karavarsamis and Hamilton 2010) for calculating annual risk (program can be downloaded from www.personal.leeds.ac.uk). Finally, these simulated exposure risks will then be compared and validated with the parasitic infection data obtain under objective 1.

- - - 1. Expected outcomes

With these results we will be able to make simulation-based recommendations of the most appropriate combination of health protection measures to achieve context specific HBT for different types of reuse practises, for example, for unrestricted irrigation (crop consumers e.g. for lettuce and onion farming) and restricted irrigation (farmers and workers e.g. working in highly mechanized or labour intensive agriculture). These results can also benefit the sanitation sector in general, and the planning of new WW and FS treatment plants in Kampala city.

1. Ethical considerations
   1. Ethical Committee Review

The study proposal will be reviewed by the institutional research commission of the Swiss TPH, the ‘Ethikkommission beider Basel’ (EKBB) and the Makerere University School of Public Health: Higher Degrees Research and Ethics Committee and the Uganda National Council of Science and Technology (UNCST) in Kampala, Uganda.

- 1. Confidentiality

For the examination of human and biological matter, standardized quality controlled methods will be applied. All study participants will be given detailed information about the purpose of the study, the extent of their involvement and their right to be treated free of charge if found with any parasitic infection will be warranted. Written informed consent will be obtained from all study participants. It will be pointed out that participation is voluntary and that the individuals may withdraw from the study at any time without further obligation. At the end of the study the participants will be informed about the result of their examination and those found infected with parasites will be treated according to the national guidelines. Currently, the Uganda authorities recommend albendazole (400mg dose) for the treatment of soil-transmitted helminths and praziquantel (40mg/kg) for the treatment of schistosomiasis.

The collected data will be stored at server of Swiss TPH and are encrypted with Secure Sockets Layer (SSL). Participants will be informed that a subset of the stool samples (100-150 samples) will be transferred to Swiss TPH in Switzerland pending polymerase chain reaction (PCR) for species detection and/or metagenomic analysis in order to provide quantitative insights into microbial population based on sequence data.

The results of the research study will be published, but subjects’ names or identities will not be revealed. Records will remain confidential. To maintain confidentiality, the PI will keep records in locked cabinets and the results of tests will be coded to prevent association with participant’s names. Data entered into the ODK data entry mask will be accessible only by authorized personnel directly involved with the study and will be encoded. Subject-specific information may be provided to other appropriate medical personnel only with the subject’s permission.

After the study has been completed all stool samples will be destroyed and research data and related material will be kept in accordance to the Medical Research Council (MRC) ethics series for a minimum of 10 years to enable understanding of what was done, how and why, which allow the work to be assessed retrospectively and repeated if necessary.

- 1. Duties of the investigators

Investigators will ensure that the study will be conducted according to the study protocol, with good epidemiological practice and the applicable regulatory requirements. Any protocol amendments will be submitted for review to the ethics committees for each study partner. As participant safety is a primary concern, any unforeseen event posing a risk to the participants will be communicated to the ethical committees. The investigators have sufficient time to properly conduct and complete the study within the agreed timeframe and have available an adequate number of qualified staff and adequate facilities for conducting the research properly and safely. Informed consent will be obtained from each participant and documented, and the version approved by the ethics committee will be adhered to. The investigators will ensure the accuracy and completeness of the data reported in the study, and will timely submit the required progress and final reports.

1. Collaboration and support

The PhD thesis is embedded in the larger RR&R project which is financed by the Swiss Agency for Development and Cooperation (SDC) and carried out within the frame of an existing research partnership between: (i) SOPH; (ii) MOH; (iii) NWSC; and (iv) the Department of Civil & Environmental Engineering of Makarere University in Kampala, Uganda and internationally: (i) the Swiss TPH (Basel, Switzerland); (ii) IWMI (Colombo, Sri Lanka); (iii) WHO (Geneva, Switzerland); (iv) the International Centre for Water Management Services (CEWAS) (Willisau, Switzerland); and (v) SANDEC (Dübendorf, Switzerland).

1. Time table

The following activities are planned within 3 months between August and October 2013.

| **Months** | **Mai-July** | **August** | | | | **September** | | | | **October** | | | |
| --- | --- | --- | --- | --- | --- | --- | --- | --- | --- | --- | --- | --- | --- |
| **Weeks** | **18-31** | **32** | **33** | **34** | **35** | **36** | **37** | **38** | **39** | **40** | **41** | **42** | **43** |
| Health risk assessment (HRA) SOPH and Swiss TPH |  |  |  |  |  |  |  |  |  |  |  |  |  |
| Follow up visit HRA  (community group discussion) |  |  |  |  |  |  |  |  |  |  |  |  |  |
| Preparation of field and laboratory work  **(3 days)** |  |  |  |  |  |  |  |  |  |  |  |  |  |
| Training workshop with field staff and lab staff **(1 days)** |  |  |  |  |  |  |  |  |  |  |  |  |  |
| Information and awareness campaign of study groups W2, F, C1, C2 **(2 days)** |  |  |  |  |  |  |  |  |  |  |  |  |  |
| Pilot testing of questionnaire and stool sample collection (**1 days)** and discussing problems and issues (**1 days)** |  |  |  |  |  |  |  |  |  |  |  |  |  |
| Interview and stool collection and analysis of **(W1) (2 days)** |  |  |  |  |  |  |  |  |  |  |  |  |  |
| Discussing issues problems **(1 day)** |  |  |  |  |  |  |  |  |  |  |  |  |  |
| Organizing data collection for W2 **(1 days)** |  |  |  |  |  |  |  |  |  |  |  |  |  |
| Interview and stool collection and analysis of **(W2)** |  |  |  |  |  |  |  |  |  |  |  |  |  |
| Organizing data collection for F, C1 and C2  **(5 days)** |  |  |  |  |  |  |  |  |  |  |  |  |  |
| Interview and stool collection and analysis of farmers **(F) (10 days)** |  |  |  |  |  |  |  |  |  |  |  |  |  |
| Interview and stool collection and analysis of control groups **(C1) (5 days)** |  |  |  |  |  |  |  |  |  |  |  |  |  |
| Interview and stool collection and analysis of control groups **(C2) (10 days)** |  |  |  |  |  |  |  |  |  |  |  |  |  |
| Starting WW and FS analysis **(1 day)** |  |  |  |  |  |  |  |  |  |  |  |  |  |
| Lab analysis for WW and FS by NWSC  **(over 2 month each week 2*16 samples)** |  |  |  |  |  |  |  |  |  |  |  |  |  |
| Buffer time |  |  |  |  |  |  |  |  |  |  |  |  |  |

Estimated budget

| **Estimated inventory needed for PhD study** | **Number** | **Unit** | **No. units** | **Total No.** | **Amount per unit**  **CHF** | **Total CHF** |
| --- | --- | --- | --- | --- | --- | --- |
| **PhD personal costs** | | | | | | |
| Flight | 1 | Flight | 1 | 1 | 1'200 | 1,200 |
| Accommodation | 1 | Month | 3 | 3 | 600 | 1,800 |
| Other expenditure (internet, printing) | 1 | Month | 3 | 3 | 100 | 300 |
| **SOPH** | | | | | | |
| **Already covered under TOR for Phase 1** | | | | | | |
| Assistance with the planning of potential epidemiological studies and/or exposure (e.g. ethical clearance procedures, coordination between partners (SoPH, MoH and NWSC) and arrangements for of sampling and data collection) | | | | | | |
| Letter of agreement for phase 1 (includes 2 BSc students + 1 senior person) | 6 | Month | 1 | 6 |  | 10,670 |
| Phase 2 | | | | | | |
| BSc student Field staff (sample collection and interviews) | 2 | Month | 3 | 6 | 400 | 2'400 |
| Transport | 8 | Week | 1 | 8 | 50 | 400 |
| Ethical clearance (Uganda) | 1 | Clearance | 1 | 1 | 700 | 700 |
| **MOH** | | | | | | |
| Kato-Katz 2x1  (staff + preparation + analysis) | 1,000 | Slides | 2 | 2,000 | 1 | 2'000 |
| Formalin-ether concentration technique  (staff + preparation + analysis) | 1,000 | Slides | 1 | 1,000 | 1 | 1'000 |
| Sludge analysis: Kato-Katz 2x1  (preparation + analysis) | 25 | Slides | 16 | 400 | 1 | 400 |
| Lab equipment (tubes, breaker glass, etc.) |  |  |  |  |  | 500 |
| **NWSC** | | | | | | |
| Wastewater and dried sludge (collection +  pre-preparation) | 18 | Samples | 16 | 288 | 2 | 576 |
| Lab equipment (tubes, breaker glass, etc.) |  |  |  |  |  | 200 |
| **Organized by Swiss TPH / Supplies** | | | | | | |
| Treatment: Albendazole | 500 | Tablet | 1 | 500 | 1 | 500 |
| Treatment: Praziquantel | 500 | Tablet | 1 | 500 | 1 | 500 |
| Local allowances for chairman/operators | 5 | Group | 1 | 5 | 50 | 250 |
| Stool collection tubes | 1,000 | Tube | 1 | 1,000 | 0.5 | 500 |
| Tablet computers | 5 | Tablet | 1 | 350 |  | 1,650 |
| Ethical clearance EKBB |  |  |  |  |  | 500 |
| Risk capital |  |  |  |  |  | 2,000 |
| **Total** |  |  |  |  |  | **28,096** |

1. References

Ayres, R. M., Mara, D. D., & Rachel, M. (1996). *Analysis of Wastewater for Use in Agriculture - A Laboratory Manual of Parasitological and Bacteriological Techniques* (pp. 1–35). Geneva, Switzerland.

Barker, C., Prain, G., Warnaars, M., Warnaars, X., Wing, L., & Wolf, F. (2007). *Impacts of urban agriculture: Highlights of Urban Harvest research and development, 2003-2006* (pp. 1–63). Peru: International Potato Center (CIP).

Becker, S. L., Vogt, J., Knopp, S., Panning4, M., Warhurst, D. C., Polman, K., Marti, H., et al. (2012). Persistent digestive disorders in the tropics: causative infectious pathogens and reference diagnostic tests. Basel, Switzerland: Department of Epidemiology and Public Health, Swiss Tropical and Public Health Institute.

Beffa, T. (2002). *The composting biotechnology: a microbial aerobic slid substrate fermentation complex process*. *happyswiss.com* (pp. 1–37). Bevaix: Compag Technology International. Retrieved from http://www.happyswiss.com/compag/pdf/THE COMPOSTING BIOTECHNOLOGY COMPLETE.pdf

Beller Consult Mott MacDonald and M&E Associates. (2004). *Sanitation Strategy & Master Plan for Kampala City: Volume 1 “Executive Summary”* (Vol. 1, pp. 1–40). Kampala, Uganda.

Blumenthal, U., & Peasey, A. (2002). *Critical review of epidemiological evidence of the health effects of wastewater and excreta use in agriculture*. *… . who. int/water_sanitation_ health/wastewater/ …* (pp. 1–42). London, England. Retrieved from http://www.who.int/entity/water_sanitation_health/wastewater/whocriticalrev.pdf

Brooker, S., Kabatereine, N. B., Smith, J. L., Mupfasoni, D., Mwanje, M. T., Ndayishimiye, O., Lwambo, N. J., et al. (2009). An updated atlas of human helminth infections: the example of East Africa. *International journal of health geographics*, *8*, 42. doi:10.1186/1476-072X-8-42

Bünger, J., Antlauf-Lammers, M., Schulz, T. G., Westphal, G. A., Müller, M. M., Ruhnau, P., & Hallier, E. (2000). Health complaints and immunological markers of exposure to bioaerosols among biowaste collectors and compost workers. *Occupational and environmental medicine*, *57*(7), 458–64. Retrieved from http://www.pubmedcentral.nih.gov/articlerender.fcgi?artid=1739988&tool=pmcentrez&rendertype=abstract

Carr, R., & Strauss, M. (2001). Excreta-related infections and the role of sanitation in the control of transmission. In L. Fewtrell & J. Bartram (Eds.), *Water Quality: Standards and Health* (pp. 89–113). London: World Health Organization. Retrieved from http://books.google.com/books?hl=en&lr=&id=d0i6rc0NO5kC&oi=fnd&pg=PA89&dq=Excreta-related+infections+and+the+role+of+sanitation+in+the+control+of+transmission&ots=7IafOTis14&sig=uEU6g1C8IUpN0PzENarP-bOP958

Cissé, G, & Tanner, M. (2001). Utilisation des eaux usées ou polluées en agriculture urbaine dans le contexte sahélien : risques sanitaires, aspects socio-économiques et contraintes pour un développement durable. In K. Wyss, D. Yemadji, G. Cissé, & M. Tanner (Eds.), *Gestion par leurs occupants d’environnements urbains défavorisés au Sahel* (pp. 31–49). Centre suisse de recherches scientifiques en Côte d’Ivoire.

Cissé, Guéladio. (1997). *Impact sanitaire de l’utilisation d'eaux polluées en agriculture urbaine. Cas du maraîchage à Ouagadougou (Burkina Faso)*. Thèse de doctorat, es science technique, École Polytechnique Fédérale de Lausanne.

Cissé, Guéladio, Koné, B., Bâ, H., Mbaye, I., Koba, K., Utzinger, J., & Tanner, M. (2011). Ecohealth and Climate Change: Adaptation to Flooding Events in Riverside Secondary Cities, West Africa. In K. Otto-Zimmermann (Ed.), *Resilient Cities: Cities and Adaptation to Climate Change Proceedings of the Global Forum 2010* (pp. 57–67). Local Sustainability 1. Retrieved from http://www.springerlink.com/index/N621023242043653.pdf

Cissé, Guéladio, Mathieu, K., Boureïma, O., & Tanner, M. (2002). Développement du maraîchage autour des eaux de barrage à Ouagadougou : quels sont les risques sanitaires à prendre en compte ? *Cahiers Agricultures*, *Valume 11*(31-8).

Cointreau, S. (1998). *Occupational and environmental health issues of solid waste management: Special emphasis on middle- and lower-income countries*. *Estados Unidos de América* (pp. 1–48). Washington, D.C. Retrieved from http://scholar.google.com/scholar?hl=en&btnG=Search&q=intitle:Occupational+and+Environmental+Health+Issues+of+Solid+Waste+Management#1

Do, T. T., Bui, T. T. H., Mølbak, K., Phung, D. C., & Dalsgaard, A. (2007). Epidemiology and aetiology of diarrhoeal diseases in adults engaged in wastewater-fed agriculture and aquaculture in Hanoi, Vietnam. *Tropical medicine & international health : TM & IH*, *12 Suppl 2*(december), 23–33. doi:10.1111/j.1365-3156.2007.01938.x

Drechsel, P., Scott, A. C., Raschid-Sally, L., Redwood, M., & Bahri, A. (2010). *Wastewater Irrigation and Health: Assessing and mitigationg risk in low-income countries* (pp. 1–404). London: International Water Management Institute.

Eddleston, M., Davidson, R., Brent, A., & Wilkinson, R. (2012). *Oxford handbook of tropical medicine* (Third Edit., pp. 1–843).

Ekane, N. (2009). *Sanitation Policies and Regulatory Frameworks for Reuse of Nutrients in Wastewater , Human Excreta and Greywater Table of Contents* (pp. 1–40). Bromma, Sweden.

Ferrer, A., Nguyen-Viet, H., & Zinsstag, J. (2012). Quantification of diarrhea risk related to wastewater contact in Thailand. *EcoHealth*, *9*(1), 49–59. doi:10.1007/s10393-012-0746-x

Fichtner Water & Transportation, & M&E Associates. (2008). *Kampala Sanitation Program / UGANDA* (Vol. 1, pp. 1–274). Kampala, Uganda.

Hoornweg, D. (1999). *What a waste: Solid waste management in Asia*. *Industry and Environment* (pp. 1–26). Washington, D.C. Retrieved from http://www.csa.com/partners/viewrecord.php?requester=gs&collection=ENV&recid=4826615

Hoornweg, D., & Bhada-Tata, P. (2012). *What a Waste: A Global Review of Solid Waste Management* (pp. 1–100). Washington. Retrieved from http://books.google.com/books?hl=en&lr=&id=qIqLmIWO7lAC&oi=fnd&pg=PA17&dq=What+a+waste&ots=O_3gpdPfA-&sig=BR5ADUveIBurokbixL_7vc-_3fA

Howard, G., Pedley, S., & Tibatemwa, S. (2006). Quantitative microbial risk assessment to estimate health risks attributable to water supply : Can the technique be applied in developing countries with limited data ? *Journal Of Water And Health*, *4*(2001), 49–65. doi:10.2166/wh.2005.058

IWMI. (2009). *Strategic research portfolio: “Resource Recovery and Reuse”* (pp. 113–127). Colombo, Sri Lanka.

John Sandford-Smith. (1990). *Eye Diseases in Hot Climates* (2nd ed.). Wright.

Kabatereine, N. B., Kemijumbi, J., Kazibwe, F., & Onapa, A. W. (1997). Human intestinal parasites in primary school children in Kampala, Uganda. *East African medical journal*, *74*(5), 311–4. Retrieved from http://www.ncbi.nlm.nih.gov/pubmed/9337010

Karavarsamis, N., & Hamilton, A. J. (2010). Estimators of annual probability of infection for quantitative microbial risk assessment. *Journal of water and health*, *8*(2), 365–73. doi:10.2166/wh.2010.045

Kayima, J., Kyakula, M., Komakech, W., & Echimu, S. P. (2008). A study of the degree of Pollution in Nakivubo Channel, Kampala, Uganda. *Journal of Applied Sciences and Environmental Management*, *12*(2), 93–98. Retrieved from http://www.ajol.info/index.php/jasem/article/view/55540

KCCA. (2004). *Review of Solid Waste Management Strategy for Kampala* (pp. 1–123).

Keiser, J., & Utzinger, J. (2005). Emerging foodborne trematodiasis. *Emerging infectious diseases*, *11*(10), 1507–14. doi:10.3201/eid1110.050614

Keiser, J., & Utzinger, J. (2009). Food-borne trematodiases. *Clinical microbiology reviews*, *22*(3), 466–83. doi:10.1128/CMR.00012-09

Kulabako, R. N., Nalubega, M., Wozei, E., & Thunvik, R. (2010). Environmental health practices, constraints and possible interventions in peri-urban settlements in developing countries--a review of Kampala, Uganda. *International journal of environmental health research*, *20*(4), 231–57. doi:10.1080/09603120903545745

Mara, D. D., Sleigh, P. a., Blumenthal, U. J., & Carr, R. M. (2007). Health risks in wastewater irrigation: Comparing estimates from quantitative microbial risk analyses and epidemiological studies. *Journal of Water and Health*, *5*(1), 39. doi:10.2166/wh.2006.055

Mara, D., & Sleigh, A. (2010). Estimation of norovirus and Ascaris infection risks to urban farmers in developing countries using wastewater for crop irrigation. *Journal of water and health*, *8*. Retrieved from http://eprints.whiterose.ac.uk/43433

Medlicott, K. O., Stenström, T.-A., Winkler, M., & Cissé, G. (2012). Draft: Sanitation Safety plans: A manual for stepwise assessment of and integrated health risk assessment management. Geneva: WHO.

Nguyen, T. L. (2005). *Solid waste management in Hanoi city* (pp. 1–6). Hanoi, Vietnam.

Nguyen-Viet, H., Zinsstag, J., Schertenleib, R., Zurbrügg, C., Obrist, B., Montangero, A., Surkinkul, N., et al. (2009). Improving environmental sanitation, health, and well-being: a conceptual framework for integral interventions. *EcoHealth*, *6*(2), 180–91. doi:10.1007/s10393-009-0249-6

Niwagaba, C. B. (2009). *Treatment Technologies for Human Faeces and Urine*. University of Uppsala.

OECD. (2010). OECD iLibrary: Statistics / OECD Factbook / 2010 / Municipal waste. Retrieved December 27, 2012, from http://www.oecd-ilibrary.org/sites/factbook-2010-en/08/02/02/index.html?contentType=&itemId=/content/chapter/factbook-2010-63-en&containerItemId=/content/serial/18147364&accessItemIds=&mimeType=text/html

Ottosson, J. (2003). *Hygiene aspects of greywater and greywater reuse hygiene aspects of greywater and greywater reuse* (pp. 1–50). Stockholme, Sweden.

RR&R project team. (2012). *Kampala: RR&R consultant report* (pp. 1–3). Colombo, Sri Lanka.

Rushton, L. (2003). Health hazards and waste management. *British Medical Bulletin*, *68*(1), 16. doi:10.1093/bmb/ldg034

Schur, N., Hürlimann, E., Stensgaard, A.-S., Chimfwembe, K., Mushinge, G., Simoonga, C., Kabatereine, N. B., et al. (2011). Spatially explicit Schistosoma infection risk in eastern Africa using Bayesian geostatistical modelling. *Acta tropica*, 1–13. doi:10.1016/j.actatropica.2011.10.006

Seidu, R., Heistad, A., Amoah, P., Drechsel, P., Jenssen, P. D., & Stenström, T.-A. (2008). Quantification of the health risk associated with wastewater reuse in Accra, Ghana: a contribution toward local guidelines. *Journal of water and health*, *6*(4), 461–71. doi:10.2166/wh.2008.118

Stenström, T. A., Seidu, R., Nelson, E., & Christian, Z. (2011). *Microbial Exposure and Health Assessments in Sanitation Technologies and Systems* (pp. 1–165). Stockholme, Sweden: Stockholm Environment Institute, EcoSan Res Series. Retrieved from http://www.sei-international.org/publications?pid=1991

Strauss, M. (2000). *Human waste (excreta and wastewater) reuse*. *Contribution to: ETC/SIDA Bibliography on Urban …* (pp. 1–31). Duebendorf, Switzerland. Retrieved from http://scholar.google.com/scholar?hl=en&btnG=Search&q=intitle:Human+Waste+(Excreta+and+Wastewater)+Reuse#0

Surinkul, N. (2009). *Integrated pathogen flow analysis (PFA) and quantitative mircobial risk assessment (QMRA) for health and environmental sanitiation planning*. Asian Institute of Technology, School of Environment, Resource and Development Thailand.

Tilley, E., Lüthi, C., Morel, A., Zurbrügg, C., & Schertenleib, R. (2008). *Compendium of Sanitation Systems and Technologies* (pp. 1–158). Dübendorf, Switzerland: Swiss Federal Institute of Aquatic Science and Technology. Retrieved from http://www.watersanitationhygiene.org/References/EH_KEY_REFERENCES/SANITATION/Latrine Design and Construction/Compendium of Sanitation Systems and Technologies (EAWAG).pdf

United Nations. (2012). *World Urbanization Prospects The 2011 Revision*. *Population*. New York.

Utzinger, J., Becker, S. L., Knopp, S., Blum, J., Neumayr, A. L., Keiser, J., & Hatz, C. F. (2012). Neglected tropical diseases: diagnosis, clinical management, treatment and control. *Swiss medical weekly*, *142*(w13727), 1–24. doi:10.4414/smw.2012.13727

Water and Sanitation Program. (2008). *MARKET ANALYSIS OF ON-SITE SANITATION & CESSPOOL EMPTYING* (pp. 1–49). Kampala, Uganda.

Water and Sanitation Program. (2010). *Marching together with a citywide sanitation stratey* (pp. 1–58). Technical Team for Sanitation Development (Tim Teknis Pembangunan Sanitasi - TTPS) & the Indonesia Sanitation Sector Development Program (ISSDP).

Westrell, T., Schönning, C., Stenström, T. A., & Ashbolt, N. J. (2004). QMRA (quantitative microbial risk assessment) and HACCP (hazard analysis and critical control points) for management of pathogens in wastewater and sewage sludge treatment and reuse. *Water science and technology : a journal of the International Association on Water Pollution Research*, *50*(2), 23–30. Retrieved from http://www.ncbi.nlm.nih.gov/pubmed/15344769

WHO. (2006a). *WHO | Guidelines for the safe use of wastewater, excreta and greywater. Volume 2: Wastewater use in agriculture* (pp. 1–222). Geneva, Switzerland: World Health Organization. Retrieved from http://www.who.int/water_sanitation_health/wastewater/gsuweg2/en/index.html

WHO. (2006b). *WHO | Guidelines for the safe use of wastwater, excreta and greywater. Volume 3: Wastewater and excreta use in aquaculture* (pp. 1–162). Geneva, Switzerland: World Health Organization. Retrieved from http://www.who.int/water_sanitation_health/wastewater/gsuweg3/en/index.html

WHO. (2006c). *WHO | Guidelines for the safe use of wastewater, excreta and greywater. Volume 1: Policy and regulatory aspects* (pp. 1–114). Geneva, Switzerland: World Health Organization. Retrieved from http://www.who.int/water_sanitation_health/wastewater/gsuweg1/en/index.html

WHO. (2006d). *WHO | Guidelines for the safe use of wastwater, excreta and greywater. Volume 4: Excreta and greywater use in agriculture* (pp. 1–204). Geneva, Switzerland: World Health Organization. Retrieved from http://www.who.int/water_sanitation_health/wastewater/gsuweg4/en/index.html

WHO | Water sanitation and health. (n.d.).*WHO*.

1. Annex
   1. Questionnaire

Provided as a separate document (Study Questionnaire / Version 1, 16.05.2015)

- 1. Patient information and Consent Form

Provided as a separate document (Patient information and Consent Form / Version 1, 16.05.2015)
